# Pre‐Diagnostic Features of Multiple Sclerosis in a Diverse UK Cohort: A Nested Case–Control Study

**DOI:** 10.1002/acn3.70175

**Published:** 2025-09-24

**Authors:** Pooja Tank, Benjamin M. Jacobs, Jonathan Bestwick, Ruth Dobson

**Affiliations:** ^1^ Wolfson Institute of Population Health Queen Mary University of London London United Kingdom; ^2^ Department of Neurology Royal London Hospital, Barts Health NHS Trust London United Kingdom

**Keywords:** deprivation, diversity, ethnicity, gender, multiple sclerosis, prodrome

## Abstract

**Background:**

Many patients with Multiple Sclerosis (MS) experience nonspecific symptoms prior to diagnosis. This period—the 'MS prodrome'—has been described in socio‐economically homogeneous cohorts to date. It remains unclear to what extent events prior to an MS diagnosis differ according to social determinants of health.

**Methods:**

We conducted a retrospective, longitudinal, population‐based nested case–control study using data from Clinical Practice Research Datalink (CPRD) Aurum. Associations between pre‐diagnostic symptoms and MS risk were evaluated using multivariable logistic regression models in MS cases and matched controls. To determine whether associations differed by potential health determinants, we tested for statistical interactions and used stratified models.

**Results:**

The study population consisted of 15,029 patients with MS (median index age 44.6, 80.3% female) and 81,027 age‐matched controls. In the 5 years preceding diagnosis, MS cases were more likely than controls to have coded autonomic (OR 1.87 [1.80–1.94]), cognitive (OR 2.57 [2.06–3.20]), neurological (OR 7.91 [7.58–8.26]), pain (OR 2.21 [2.12–2.29]) and psychiatric (OR 1.75 [1.69–1.82]) symptoms. The direction of effect was consistent across gender, ethnicity, deprivation and location (urban vs. rural) strata, with over‐representation of neurological symptoms in males and those living in urban areas. No statistically significant interaction between factors was found.

**Conclusion:**

We report a consistent relationship between the occurrence of prodromal symptoms and MS risk across a diverse UK cohort. No associations were specific to a particular ethnic, gender or socio‐economic group. These findings strengthen the concept of pre‐diagnostic symptoms reflecting a potential opportunity to identify those at the earliest stages of MS.

## 1 Introduction

Multiple sclerosis (MS) is a chronic, disabling neurodegenerative disease and a leading cause of neurological dysfunction and disability in young adults [1]. MS is a global disease and can occur in people of any ethnicity [2]. Approximately 2.2–2.3 million people worldwide live with MS, of whom around 150,000 live in the United Kingdom (UK) [3].

Previous studies have demonstrated that people with MS often experience nonspecific symptoms for years prior to the sentinel clinical event leading to an MS diagnosis [4]. This phenomenon has been termed the ‘MS prodrome’ and has been observed in several different electronic healthcare record (EHR) datasets [5, 6, 7, 8, 9, 10]. Eliciting core features during this period may be beneficial in three respects: 1) to aid earlier diagnosis of MS (enabling earlier treatment which may improve long‐term outcomes); 2) to help study the pathogenesis of disease by identifying people at the earliest stages of disease initiation, before the onset of overt neuroinflammation; and 3) to identify individuals at high risk of progression to clinically definite MS for enrolment in trials of preventive interventions.

However, for the MS prodrome to be a scientifically useful and equitable concept, it needs to be generalisable across populations. Events captured as part of this prodrome may, for some, reflect pre‐diagnostic manifestations of MS which have not been recognised, including by healthcare providers. Little is known about whether or how events during this period differ depending on ethnicity and socio‐economic status, both of which are important social determinants of health that could plausibly influence interactions with healthcare provider(s) [11]. If the MS prodrome is to have a role in the diagnosis of MS, it is essential we understand the heterogeneity of this phase of the disease across the entirety of the population.

We used data from the Clinical Practice Research Datalink (CPRD) Aurum to explore this pre‐diagnostic phase of MS in a large, ethnically diverse and socio‐economically diverse UK population. CPRD is a longitudinal population‐based study with anonymised, linked electronic healthcare records covering ~20% of the UK population, which has been shown to be representative of the wider population [12, 13, 14]. A key advantage of CPRD is that it combines primary and secondary care records, in addition to census‐based metrics such as area‐level deprivation. This provides a holistic picture of the interplay between demographic factors and health‐related outcomes in a representative UK population. In addition, the universal access to healthcare in the UK means that, in principle, these data are more robust to confounding by socio‐economic deprivation than data from insurance‐based cohorts.

We set out to describe features occurring prior to an MS diagnosis in a UK cohort, and to determine whether socio‐economic deprivation or ethnicity was associated with the heterogeneity of these features and/or the heterogeneity of the temporal sequence of events. Our primary study aims were to understand whether, given differences in healthcare access and MS outcomes associated with demographic social determinants of health [15], recording of prediagnostic symptoms varied according to reported deprivation, gender or ethnicity within the UK. Our secondary aims were to extend this analysis to other social determinants of health captured within CPRD, namely gender and urban/rural location.

## 2 Methods

### 2.1 Study Design

We conducted a retrospective, population‐based nested case–control study using longitudinal data from the Clinical Practice Research Datalink (CPRD). Included participants were registered to general practices in England and had an index date of MS diagnosis/dummy diagnosis between 01 January 1990 and 01 March 2022 recorded within CPRD.

### 2.2 Data Source

Patients were identified from CPRD (previously General Practice Research Database). CPRD is a nationally validated and generally representative database containing retrospective and prospective electronic healthcare records of general practices (GP) across the UK (England, Scotland, Wales and Northern Ireland) [12]. The database was initiated in 1987 and is managed by the Medicines and Healthcare products Regulatory Agency (MHRA) and the National Institute for Health Research (NIHR) [16].

For this study, CPRD Aurum (EMIS software, covering ~20% of current UK population) was used [16]. This contains routinely collected de‐identified patient information including demographic, clinical and prescription data. These data sources include rich information on medical diagnoses, symptoms and medications issued via primary care. The population within CPRD has been shown to be reasonably generalisable to the UK population in terms of region, age and sex [12, 13, 14], and is reasonably accurate across a wide range of diagnoses [17]. Data validity in terms of patient‐line‐level data is assessed prior to data export by the data controller; given the lifelong national coverage of the NHS, data completeness is high in terms of recorded medical diagnoses, particularly where linked secondary care datasets are used [18].

Linked datasets were used to supplement primary care data. NHS number provides a single identifier that moves with individuals during life, enabling linkage across area level (deprivation and urban/rural location) and secondary care data (Hospital Episode Statistics; HES). HES data are a national collection of records from all NHS‐funded hospital admissions in England. CPRD‐HES data date back to 1997 for inpatient admission and 2003 for outpatient encounters. Diagnosis and clinical characteristics in HES are recorded using the International Classification of Diseases version 10 (ICD‐10) coding schema.

We acquired patient‐level and practice‐level index of multiple deprivation (IMD) 2019 quintiles from CPRD. The IMD is an area‐level measure derived from a combination of variables (income, employment, education and skills, health, housing, crime, access to services and living environment) measured at the lower layer super output area (LSOA) level. IMD was calculated as quintiles ranging from 1 (least deprived) to 5 (most deprived) according to both individual patient and general practice. Patient‐level urban/rural classification was produced by the Office for National Statistics using the 2011 census based on the patient's postcode.

Data were extracted from the May 2022 build containing anonymised data of over 41 million patients from 1491 UK practices. Only patients with acceptable ‘research quality’ data were included in this study (≥ 93% of the total population). This study was restricted to patients in England due to HES and patient‐level deprivation data availability (297 patients from Northern Ireland removed).

### 2.3 Study Participants

The study population was derived from 41,092,910 participants present in CPRD Aurum in May 2022 who had a recorded sex and at least 1 day of GP follow‐up between 01 January 1990 and 01 March 2022.

Cases meeting the following criteria were selected for inclusion: at least 2 MS‐related diagnoses within the study period, with ≥ 1 MS (high confidence) 'Medcode' diagnostic code in primary care and ≥ 1 of either MS diagnosis in primary care or HES (ICD‐10 code: G35) or a specialist neurology referral. Since an MS diagnosis may not reflect MS onset, a specialist neurology referral was used as a proxy for MS onset (index date); where this was not available, the earliest record of an MS or related diagnosis in primary or secondary care was used as the index date (Figure Figure S1). Full details of the ‘Medcode’ diagnostic codes used are given in Table S1. Cases were required to have a recorded IMD, and be aged ≥ 18 years.

Initially, 10 controls who did not have any MS diagnosis were randomly matched to each case by year of birth as part of dataset generation; as CPRD permissions generally exclude access to the entire dataset matching was performed prior to exclusion of controls without a minimum dataset available. Controls with a HES MS diagnosis, ≤ 5 years of retrospective data prior to the index date, no recorded IMD, or no corresponding matched case were then excluded. Controls were assigned the same index date as their matched case.

**FIGURE 1:**
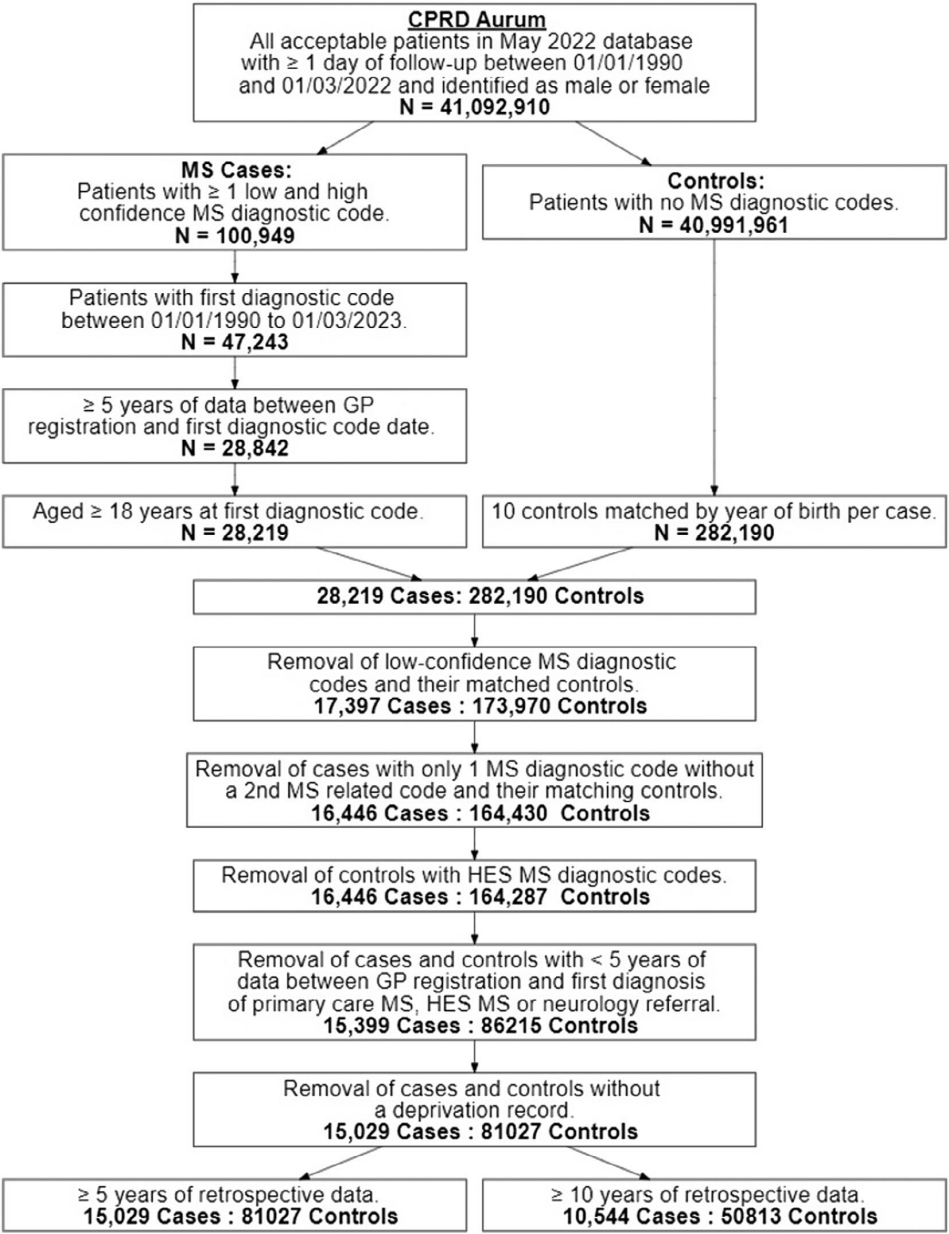
Study flow chart.

All cases and controls were required to have ≥ 5 years of retrospective data from CPRD registration to index date, ensuring a minimum of 5 years of complete data. In total, 15,029 cases and 81,027 controls (5.4 controls per case) with ≥ 5 years of retrospective data and 10,806 cases and 54,420 controls (5.0 controls per case) with ≥ 10 years of retrospective data were included in this study (Figure 1).

### 2.4 Time Intervals

In line with previous studies across neurological diseases, time intervals explored in this study were prespecified as all time, 0–2, 2–5 and 5–10 years prior to the index date [19]. We used data from patients with ≥ 5 years of retrospective data to examine all time, 0–2 and 2–5‐year time intervals and only patients with ≥ 10 years of retrospective data to examine the 5–10‐year time interval.

### 2.5 Variables

Clinical characteristics and patient information were extracted either from predefined data fields (e.g., sex) or from 'Medcodes'—a coding system used in CPRD that links to various health concepts, diagnoses and problem codes.

#### 2.5.1 Pre‐Diagnostic Symptoms and Categories

Potential prodromal symptoms of MS have been previously described and identified [5, 6, 7, 8, 9, 10]. These were extracted and categorised based on prior research for the purposes of this study. Symptoms were categorised into 5 groups: Neurological, Autonomic, Cognitive, Psychiatric and Pain (see Table S2 for full code lists and categorisation). Patients who had at least 1 record of a particular symptom in a specified time interval to the prior index date were considered symptomatic. Symptoms were identified within the time period of 0–2, 2–5, 5–10 years and any time to the index date. Binary variables were created where 0 = no and 1 = yes. Those without a recorded symptom were classified as asymptomatic rather than missing.

#### 2.5.2 Demographic Variables

Demographic variables including recorded sex (male/female), age at index date, IMD, location (Urban/Rural) and ethnicity were extracted for each patient.

Ethnicity was derived from both primary and secondary care [13]. As ethnicity was more frequently recorded in secondary care, HES was used as the main source to classify patients. We used 5 ethnic groups as per UK Census categories: White (British, Irish, other white), Black (Black African, Black Caribbean, other Black), South Asian (Bangladeshi, Pakistani, Indian, other South Asian), Other/Mixed (Chinese, Mixed any other ethnicity) and Unknown. Since ethnicity is a self‐identified variable, this may change; however, most patients in HES selected the same ethnic group for each admission. Where more than one category was selected by an individual, the most frequently recorded ethnicity was assigned to that patient (excluding unknowns). For those where this was not recorded in HES, primary care data were used in the same manner to classify the remaining patients.

### 2.6 Missing Data

People with missing IMD (*N* = 5558, 5%) were excluded from the analysis as all had missing urban/rural classification and 95% missing ethnicity. Those with missing ethnicity only (*N* = 25,531, 32%) were assigned as ‘Unknown’ (Table S3a). To determine whether the cohort with > 10 years of retrospective data might represent a demographically nonrandom sample of the population, we compared the characteristics between those with and those without 10 years of retrospective data. Demographic and symptom distributions between the two groups were similar in terms of ethnicity, recorded sex, IMD, location (urban vs. rural), grouped symptoms, MS status and age at index date (Table S3b).

### 2.7 Statistical Analysis

Demographic characteristics were summarised for cases and controls for the two sample populations (≥ 5 and ≥ 10 years of retrospective data). Continuous data are presented in terms of the mean (SD) for normally distributed variables or median [IQR] where the data deviated from normality. Categorical data are presented as counts and percentages.

To determine the strength of association between each candidate prodromal symptom, identified a priori as above, and subsequent MS, we used multivariable unconditional logistic regression models with MS disease status as the outcome. Models were adjusted for age, gender, ethnicity, urban/rural location and patient‐level IMD.

We used the models described below to ensure that the choice of confounding covariates did not alter the result. To quantify whether the association differed by demographic background (ethnicity, patient‐level IMD, sex and urban/rural location), we included an interaction term (between the demographic variable and the tested symptom) and tested the null hypothesis that this term was a null (i.e., Interaction Odds Ratio = 1). Specifically, we used the following model formulations:

MS Status ~ Age + Sex + Symptom × Demographic Variable (Unadjusted Model)

MS status ~ Age + Sex + Ethnicity + IMD (patient level) + urban/rural Location + Symptom × demographic variable (adjusted model).

In order to increase power, deprivation status was summarised into the following categories: IMD 1–2, IMD 3, IMD 4–5 (less deprived/average/more deprived; grouped due to similar odds for MS and to improve power). Likelihood‐ratio tests of models including interaction terms versus models without interactions were used to evaluate heterogeneity by demographic background. Analysis was first conducted for symptom categories followed by each individual symptom within the category.

To explore whether the temporal sequence of the MS prodrome we considered, discrete time windows in the years leading up to an MS diagnosis, we repeated the above analyses using data exclusively within 0–2 years of index date, 2–5 years and 5–10 years.

#### 2.7.1 Sensitivity Analyses

A sensitivity analysis was performed to account for changes in the MS diagnostic criteria over time. The sample population was stratified by index date into three distinct time periods reflecting shifts in diagnostic criteria: Jan 1990–Dec 2000 (Poser's criteria) [20], Jan 2001–Dec 2010 (McDonald 2000 and 2005 revision) [21, 22] and Jan 2011–Mar 2022 (McDonald 2010 and 2017 revision) [23, 24].

All analysis was conducted using R software version 4.1.1 [25]. A *p*‐value < 0.05 was considered statistically significant. For multiple comparisons, *p*‐values were corrected using Bonferroni correction where appropriate. Data are presented as odds ratios (ORs) with 95% confidence intervals (CIs). This research used Queen Mary's Apocrita HPC facility, supported by QMUL Research‐IT [26].

## 3 Results

### 3.1 Study Characteristics

The study population consisted of 15,029 patients with MS and 81,027 age‐matched controls. The median age at index date was similar (44.64 ± 12.27 years for cases and 44.75 ± 11.59 for controls). Patients with MS were predominantly female (Cases: 70.3% vs. Controls: 50.2%) and White (Cases: 93.4% vs. Controls: 87.9% of those with recorded ethnicity). People of Black (OR: 0.61 [0.54–0.68]), South Asian (OR: 0.50 [0.45–0.56]), and Mixed/Other (OR: 0.49 [0.43–0.56]) ethnicities were less likely to be diagnosed with MS compared to those of White ethnicity. Cases and controls were similar in terms of age at GP registration, follow‐up data prior to index date, and urban/rural location (Table 1). Those living in more deprived areas were less likely to be diagnosed with MS (IMD 4 OR: 0.89 [0.84–0.94] and IMD 5 OR: 0.82 [0.77–0.87]) compared to the least deprived area (IMD 1). People living in rural areas were more likely to be diagnosed with MS (OR: 1.09 [1.04–1.15]) in than urban areas. Details of the study population are given in Table 1.

**TABLE 1:** Demographic and clinical characteristics of included patients with at least 5 years’ retrospective data availability according to MS status and odds ratios for the risk of MS from multivariable logistic regression.

	≥ 5 years of retrospective data		Multivariable Analysis (> 5 years of retrospective data)		
	Case *N* = 15,029	Control *N* = 81,027	OR (CL)	*p*	Bonferroni corrected *p*
Age at Registration (mean (SD))	26.9 (14.7)	29.0 (14.1)			
Age at Index date (mean (SD))	44.6 (12.3)	44.6 (11.6)			
Time prior Index date (median [IQR])	14.7 [9.1–23.2]	12.4 [8.1–19.5]			
Gender^a^ (*N* [%])					
Female	10,572 (70.3)	40,651 (50.2)	ref	ref	ref
Male	4457 (29.7)	40,376 (49.8)	0.45 [0.43–0.46]	< 0.0001*	< 0.0001*
Ethnicity^a^ (*N* [%])					
White	13,636 (90.7)	53,110 (65.5)	ref	ref	ref
Black	338 (2.2)	2264 (2.8)	0.61 [0.54–0.68]	< 0.0001*	< 0.0001*
Asian	349 (2.3)	2886 (3.6)	0.50 [0.45–0.56]	< 0.0001*	< 0.0001*
Mixed/Other	270 (1.8)	2135 (2.6)	0.49 [0.43–0.56]	< 0.0001*	< 0.0001*
Unknown	436 (2.9)	20,632 (25.5)	0.09 [0.08–0.09]	< 0.0001*	< 0.0001*
IMD (Patient level)^a^ (*N* [%])					
1	3486 (23.2)	17,432 (21.5)	ref	ref	ref
2	3454 (23.0)	16,813 (20.7)	1.01 [0.96–1.07]	0.7058	1
3	3017 (20.1)	16,033 (19.8)	0.95 [0.90–1.00]	0.0671	0.2684
4	2792 (18.6)	16,350 (20.2)	0.89 [0.84–0.94]	< 0.0001*	0.0002*
5	2280 (15.2)	14,399 (17.8)	0.82 [0.77–0.87]	< 0.0001*	< 0.0001*
IMD (Practice level)^b^ (*N* [%])					
1	2699 (18.0)	14,155 (17.5)	ref	ref	ref
2	2704 (18.0)	13,803 (17.0)	1.02 [0.96–1.08]	0.5061	1
3	3307 (22.0)	17,252 (21.3)	1.03 [0.97–1.09]	0.3852	1
4	3153 (21.0)	18,300 (22.6)	0.93 [0.88–0.99]	0.0221*	0.0882
5	3166 (21.1)	17,517 (21.6)	0.96 [0.91–1.02]	0.1743	0.6971
Location^a^ (*N* [%])					
Urban	12,263 (81.6)	68,408 (84.4)	ref	ref	ref
Rural	2766 (18.4)	12,619 (15.6)	1.09 [1.04–1.15]	0.0005*	0.0005*
Potential prodromal symptom					
Autonomic symptoms^c^ (*N* [%])	5751 (38.3)	16,672 (20.6)	1.87 [1.80–1.94]	< 0.0001*	< 0.0001*
Cognitive symptoms^c^ (*N* [%])	130 (0.9)	244 (0.3)	2.57 [2.06–3.20]	< 0.0001*	< 0.0001*
Neurological symptoms^c^ (*N* [%])	6664 (44.3)	6096 (7.5)	7.91 [7.58–8.26]	< 0.0001*	< 0.0001*
Pain symptoms^c^ (*N* [%])	6515 (43.3)	17,102 (21.1)	2.21 [2.12–2.29]	< 0.0001*	< 0.0001*
Psychiatric symptoms^c^ (*N* [%])	5176 (34.4)	14,617 (18.0)	1.75 [1.69–1.82]	< 0.0001*	< 0.0001*

*Note:* Continuous variables are presented as mean ± SD for normally distributed variables and as median (interquartile range) for skewed variables. Categorical data was presented as counts and percentages (%). Multivariable unconditional logistic regression was used to assess the association between the risk of MS and each individual variable gender, ethnicity, IMD (practice or patient level), location and symptoms. Models were generated from patients with at least 5 years of retrospective data. Odds Ratio for MS, 95% CI, and two‐sided *p*‐value are reported.

Abbreviations: CI, confidence interval; IMD, index level of multiple deprivation. IQR, interquartile range; OR, odds ratio; SD, standard deviation.

^a^ Model: MS status ~ Age + Gender + Ethnicity + Location + IMD (patient level). ORs interpreted as the odds of developing MS compared to the reference group.

^b^ Model: MS status ~ Age + Gender + Ethnicity + Location + IMD (practice level). ORs interpreted as the odds of developing MS compared to the reference group.

^c^ Model: MS status ~ Age + Gender + Ethnicity + Location + IMD (patient level) + Symptom. ORs interpreted as the odds of developing subsequent MS with the selected symptom compared to the absence of that symptom.

* Significantly different from the reference group after controlling for all other demographic variables (gender, ethnicity, deprivation and Location).

### 3.2 Pre‐Diagnostic MS Symptoms

**FIGURE 2:**
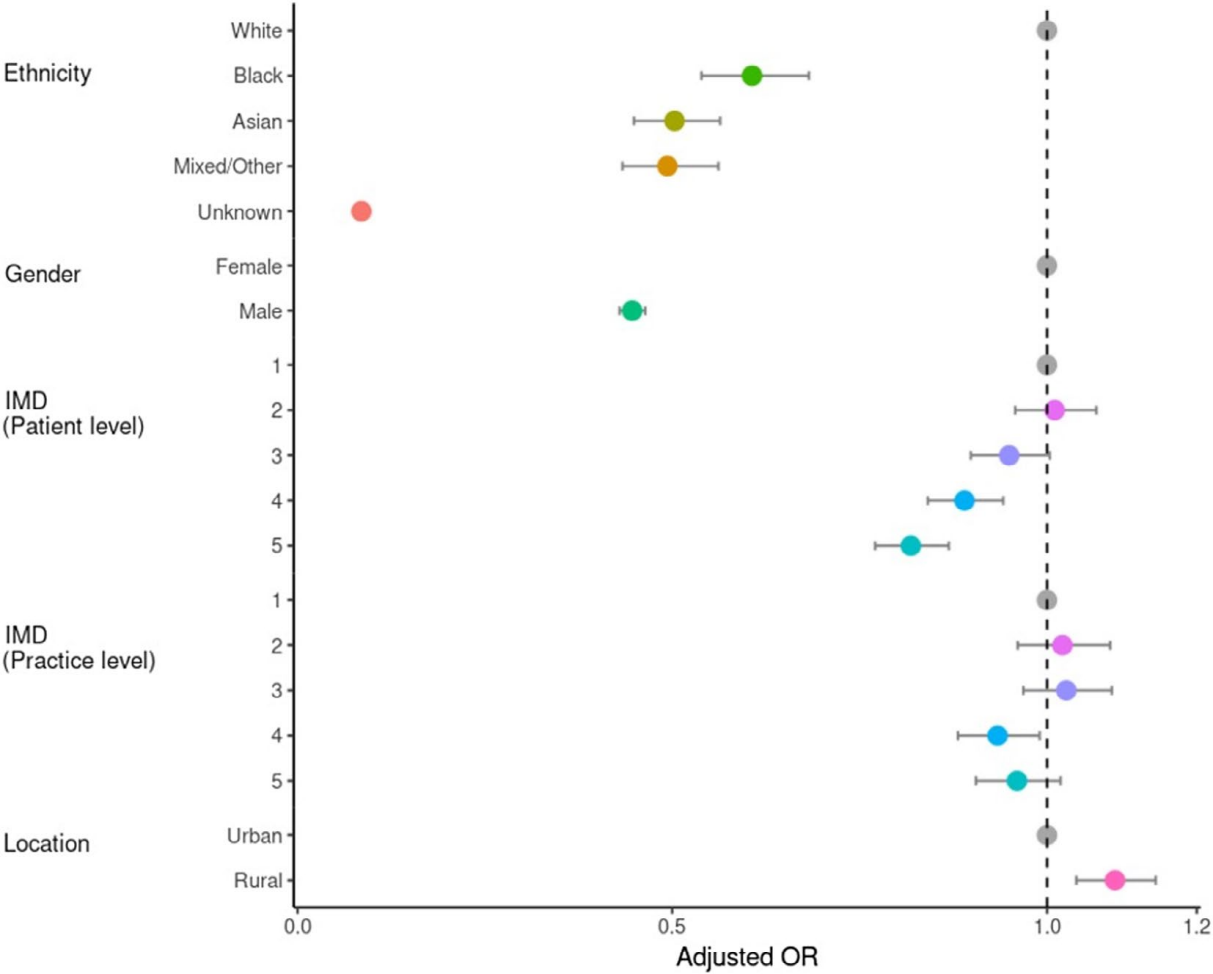
Forest plot of the risk of MS by demographic background. OR and CL displayed are detailed in Table 1, and reference level depicted in grey.

Autonomic (Cases: 38.3% vs. Controls: 20.6%), cognitive (Cases: 0.9% vs. Controls: 0.3%), neurological (Cases: 44.3% vs. Controls: 7.5%), pain (Cases: 43.3% vs. Controls: 21.1%) and psychiatric (Cases: 34.4% vs. Controls: 18.0%) symptoms occurred more frequently in the 5 years prior to the index date in cases than in controls. A similar pattern was observed in those with ≥ 10 years of retrospective data. Unsurprisingly, neurological symptoms showed the strongest association with subsequent MS (OR: 7.91 [7.58–8.26]) followed by cognitive symptoms (OR: 2.57 [2.06–3.20]), pain (OR: 2.21 [2.12–2.29]), autonomic symptoms (OR: 1.87 [1.80–1.94]) and psychiatric symptoms (OR: 1.75 [1.69–1.82]) **(**Figure 2 **)**.

### 3.3 Heterogeneity in the MS Prodrome

To explore whether the MS prodrome differed according to demographics, we explored statistical interactions between selected characteristics and each prodromal symptom (Tables 2, 3, 4, 5, and S4). Importantly, the direction of effect for all prodromal symptoms was consistent across sex, ethnicity, deprivation and location (urban vs. rural).

**TABLE 2:** Results from a univariate logistic regression model examining the interaction between pre‐diagnostic symptoms and gender on the risk of MS.

		All pre‐diagnostic years				0–2 years prior diagnosis				2–5 years prior diagnosis				5–10 years prior diagnosis			
		Case: Control	Unadjusted OR (95% CI)	*Pp*‐int	Bonferroni corrected *P*‐int	Case: Control	Unadjusted OR (95% CI)	*p*‐int	Bonferroni corrected *P*‐int	Case: Control	Unadjusted OR (95% CI)	*p*‐int	Bonferroni corrected *P*‐int	Case: Control	Unadjusted OR (95% CI)	*p*‐int	Bonferroni corrected *P*‐int
**Symptoms**	**Gender**																
Autonomic symptoms	Female	4194: 9429	2.21 (2.11–2.31)	0.0019*	0.0387*	1791: 3008	2.61 (2.45–2.79)	0.0004*	0.0242*	1780: 3650	2.07 (1.95–2.21)	0.0725	~1	1812: 3990	2.18 (2.03–2.35)	0.7540	~1
	Male	1557: 7243	2.51 (2.35–2.69)			718: 2366	3.19 (2.91–3.50)			642: 2802	2.30 (2.09–2.52)			589: 2972	2.23 (1.99–2.49)		
Cognitive symptoms	Female	89: 121	2.83 (2.15–3.73)	0.7740	~1	48: 40	4.61 (3.03–7.01)	0.1731	~1	19: 34	2.13 (1.21–3.73)	0.7386	~1	17: 30	2.39 (1.14–5.01)	0.8077	~1
	Male	41: 123	3.02 (2.12–4.31)			26: 32	7.34 (4.37–12.33)			7: 35	1.80 (0.80–4.06)			6: 38	2.05 (0.77–5.51)		
Neurological symptoms	Female	4690: 3528	8.52 (8.09–8.97)	< 0.0001*	< 0.0001*	3323: 961	18.94 (17.55–20.43)	< 0.0001*	< 0.0001*	1235: 1190	4.41 (4.06–4.79)	0.0004*	0.0249*	1043: 1261	3.84 (3.46–4.25)	0.0004*	0.0266*
	Male	1974: 2568	12.01 (11.18–12.90)			1425: 688	27.17 (24.63–29.98)			472: 826	5.72 (5.09–6.44)			390: 823	5.31 (4.58–6.16)		
Pain symptoms	Female	4754: 9564	2.66 (2.54–2.78)	0.0857	~1	2266: 2743	3.78 (3.55–4.01)	0.0063*	0.3786	2108: 3533	2.62 (2.47–2.78)	0.3638	~1	2073: 4232	2.46 (2.30–2.64)	0.4784	~1
	Male	1761: 7538	2.85 (2.67–3.04)			792: 1897	4.39 (4.02–4.80)			683: 2492	2.75 (2.51–3.01)			675: 2994	2.58 (2.32–2.87)		
Psychiatric symptoms	Female	3998: 9005	2.14 (2.04–2.24)	0.3504	~1	1730: 2356	3.18 (2.98–3.40)	0.0249*	~1	1807: 3042	2.55 (2.39–2.71)	0.3987	~1	1768: 3913	2.25 (2.09–2.42)	0.4201	~1
	Male	1178: 5612	2.23 (2.07–2.40)			489: 1307	3.68 (3.30–4.11)			476: 1718	2.69 (2.42–2.99)			459: 2248	2.39 (2.10–2.73)		

*Note:* Data were modelled for symptoms present at 0–2, 2–5, 5–10, and all pre‐diagnostic years adjusting for matching variable age and gender. Model: MS status ~ Age + Gender + Symptom x demographic variable (unadjusted model). Odd ratios (OR) and 95% confidence intervals (CI) are reported. ORs interpreted as the odds of developing subsequent MS with the given demographic background and symptom compared to the absence of that symptom at x years prior diagnosis. *p*‐values and Bonferroni corrected *p*‐values are reported for symptom*demographic variable interaction *p*‐values.

* *P*‐values considered statistically significant. – Counts too low to measure OR.

The magnitude of the association between neurological symptoms at any time and subsequent MS appeared to be greater for males than females (OR: M: 12.01[11.18–12.90] vs. F: 8.52[8.09–8.97]). This persisted across all time intervals (Table 2). The relationship between autonomic symptoms and subsequent MS was more pronounced among males than females (OR: M: 2.51 [2.35–2.69] vs. F: 2.21 [2.11–2.31]). There was no evidence of an interaction between sex and pain or psychiatric symptoms, with both sexes having similar odds for subsequent MS.

The association between psychiatric symptoms and subsequent MS was stronger in Black, South Asian, and Mixed/Other patients vs. White patients at any time (Bonferroni corrected interaction *p*‐value: < 0.0001), from 0 to 2 and from 2 to 5 years prior to diagnosis (Table 3). Neurological symptoms in Black and Mixed/Other patients had a more marked association with subsequent MS than in White patients (OR: Black: 10.48 [8.07–13.62], Mixed/Other: 13.82 [10.34–18.48] vs. White: 7.59 [7.25–7.94]) at any time prior to diagnosis. The association between autonomic symptoms and pain symptoms, and subsequent MS was also higher in Black patients and Mixed/Other patients vs. White patients (Table 3 **)**.

**TABLE 3:** Results from a univariate logistic regression model examining the interaction between pre‐diagnostic symptoms and ethnicity on the risk of MS.

Symptoms	Ethnicity	All pre‐diagnostic years				0–2 years prior diagnosis				2–5 years prior diagnosis				5–10 years prior diagnosis			
		Case: Control	Unadjusted OR (95% CI)	*p*‐int	Bonferroni corrected *P*‐int	Case: Control	Unadjusted OR (95% CI)	*p*‐int	Bonferroni corrected *P*‐int	Case: Control	Unadjusted OR (95% CI)	*p*‐int	Bonferroni corrected *P*‐int	Case: Control	Unadjusted OR (95% CI)	*p*‐int	Bonferroni corrected *p*‐int
Autonomic symptoms	White	5233: 13395	1.81 (1.74–1.88)	< 0.0001*	0.0002*	2291: 4584	2.18 (2.06–2.30)	< 0.0001*	< 0.0001*	2214: 5384	1.70 (1.61–1.80)	0.0005*	0.0289*	2181: 5566	1.73 (1.62–1.85)	0.0112*	0.6733
	Black	172: 628	2.56 (2.02–3.23)			68: 196	2.53 (1.86–3.44)			74: 243	2.24 (1.67–3.01)			85: 323	2.49 (1.73–3.58)		
	Asian	154: 735	2.23 (1.78–2.81)			74: 229	3.12 (2.33–4.19)			58: 308	1.64 (1.20–2.23)			59: 347	1.32 (0.89–1.94)		
	Mixed/Other	119: 443	2.92 (2.24–3.80)			42: 117	3.13 (2.14–4.60)			49: 164	2.56 (1.80–3.64)			53: 207	3.03 (1.96–4.68)		
	Unknown	73: 1471	2.61 (2.01–3.37)			34: 248	7.66 (5.26–11.15)			27: 353	3.84 (2.56–5.75)			23: 519	2.44 (1.46–4.10)		
Cognitive symptoms	White	114: 195	2.40 (1.90–3.05)	0.1768	~1	62: 59	4.35 (3.02–6.27)	0.1066	~1	25: 54	1.85 (1.15–3)	0.5326	~1	22: 53	2.03 (1.09–3.79)	0.5916	~1
	Black	7: 7	5.80 (2.00–16.81)			4: 3	8.00 (1.76–36.39)			1: 2	2.57 (0.23–28.42)			1: 1	3.87 (0.24–62.09)		
	Asian	4: 16	1.98 (0.65–6.03)			3: 2	12.92 (2.07–80.65)			0: 5	—			0: 7	—		
	Mixed/Other	2: 10	1.72 (0.37–8.05)			2: 4	3.35 (0.60–18.65)			0: 3	—			0: 4	—		
	Unknown	3: 16	9.93 (2.85–34.62)			3: 4	37.08 (8.06–170.48)			0: 5	—			0: 3	—		
Neurological symptoms	White	6079: 5051	7.59 (7.25–7.94)	< 0.0001*	< 0.0001*	4324: 1371	17.30 (16.20–18.48)	< 0.0001*	< 0.0001*	1551: 1666	3.88 (3.61–4.18)	0.0109*	0.6548	1323: 1693	3.42 (3.12–3.74)	0.1318	~1
	Black	172: 196	10.48 (8.07–13.62)			121: 56	20.64 (14.53–29.31)			50: 67	5.23 (3.54–7.74)			42: 77	3.94 (2.28–6.81)		
	Asian	175: 296	8.55 (6.69–10.92)			130: 107	15.13 (11.26–20.32)			51: 133	3.39 (2.40–4.81)			27: 107	2.15 (1.24–3.72)		
	Mixed/Other	146: 167	13.82 (10.34–18.48)			105: 37	36.61 (24.24–55.31)			39: 71	4.82 (3.16–7.33)			29: 70	3.91 (2.25–6.78)		
	Unknown	92: 386	14.92 (11.58–19.23)			68: 78	51.23 (36.19–72.52)			16: 79	10.21 (5.88–17.72)			12: 137	6.78 (3.55–12.92)		
Pain symptoms	White	5927: 13618	2.14 (2.06–2.23)	< 0.0001*	0.0007*	2776: 3889	3.09 (2.93–3.26)	< 0.0001*	< 0.0001*	2525: 4948	2.09 (1.99–2.21)	< 0.0001*	0.0037*	2476: 5719	1.93 (1.82–2.06)	0.0227*	~1
	Black	180: 614	2.96 (2.34–3.75)			91: 166	4.42 (3.31–5.92)			81: 249	2.48 (1.86–3.30)			80: 301	2.19 (1.50–3.20)		
	Asian	189: 835	2.71 (2.16–3.40)			98: 280	3.38 (2.59–4.42)			99: 365	2.58 (1.99–3.35)			96: 400	1.94 (1.39–2.71)		
	Mixed/Other	138: 480	3.43 (2.64–4.46)			56: 113	4.43 (3.11–6.31)			53: 168	2.70 (1.92–3.81)			71: 232	3.29 (2.22–4.89)		
	Unknown	81: 1555	2.77 (2.17–3.55)			37: 192	9.63 (6.66–13.93)			33: 295	5.45 (3.74–7.93)			25: 574	3.38 (2.11–5.41)		
Psychiatric symptoms	White	4778: 12036	1.68 (1.61–1.75)	< 0.0001*	< 0.0001*	2050: 3249	2.47 (2.33–2.62)	< 0.0001*	< 0.0001*	2099: 4096	1.97 (1.87–2.09)	< 0.0001*	0.0002*	2051: 5133	1.74 (1.62–1.86)	0.0094*	0.5636
	Black	125: 406	2.51 (1.96–3.21)			52: 85	4.25 (2.94–6.16)			62: 132	3.50 (2.51–4.87)			61: 177	2.57 (1.68–3.92)		
	Asian	115: 426	2.61 (2.03–3.34)			47: 99	4.11 (2.83–5.96)			55: 145	3.16 (2.26–4.42)			51: 184	2.34 (1.51–3.65)		
	Mixed/Other	94: 354	2.49 (1.88–3.28)			43: 73	5.21 (3.47–7.83)			42: 108	3.28 (2.23–4.83)			47: 164	3.55 (2.23–5.65)		
	Unknown	64: 1395	2.26 (1.72–2.96)			27: 157	8.34 (5.46–12.74)			25: 279	4.04 (2.65–6.16)			17: 503	2.31 (1.28–4.18)		

* *p*‐values considered statistically significant. – Counts too low to measure OR.

There was no evidence of an interaction between deprivation and rural–urban location and any of the selected symptoms at any time prior to diagnosis **(**Tables 4, 5 **)**.

**TABLE 4:** Results from a univariate logistic regression model examining the interaction between pre‐diagnostic symptoms and deprivation (as measured by person‐level IMD) on the risk of MS.

Symptoms	All pre‐diagnostic years					0–2 years prior diagnosis				2–5 years prior diagnosis				5–10 years prior diagnosis					
	IMD	Case: Control	Unadjusted OR (95% CI)	*p*‐int	Bonferroni corrected *P*‐int	Case: Control	Unadjusted OR (95% CI)	*P*‐int	Bonferroni corrected *P*‐int	Case: Control	Unadjusted OR (95% CI)	*p*‐int	Bonferroni corrected *P*‐int	Case: Control	Unadjusted OR (95% CI)	*p*‐int	Bonferroni corrected *P*‐int	*P*‐int	Bonferroni corrected *P*‐int
Autonomic symptoms	IMD 1–2	2614: 7046	2.26 (2.14–2.39)	0.5133	~1	1123: 2306	2.66 (2.45–2.87)	0.0457*	~1	1098: 2772	2.06 (1.91–2.23)	0.4021	~1	1080: 2878	2.08 (1.90–2.28)	0.1311	~1		
	IMD 3	1164: 3257	2.40 (2.20–2.61)			500: 1096	2.68 (2.38–3.01)			497: 1264	2.24 (2.00–2.51)			490: 1354	2.46 (2.15–2.82)				
	IMD 4–5	1973: 6369	2.32 (2.18–2.48)			886: 1972	3.05 (2.80–3.33)			827: 2416	2.20 (2.01–2.39)			831: 2730	2.23 (2.01–2.47)				
Cognitive symptoms	IMD 1–2	46: 91	2.69 (1.87–3.87)	0.7799	~1	28: 30	5.12 (3.02–8.68)	0.5075	~1	6: 31	0.97 (0.40–2.35)	0.0083*	0.4981	11: 26	2.67 (1.07–6.66)	0.7711	~1		
	IMD 3	28: 48	2.94 (1.82–4.73)			21: 13	8.02 (3.96–16.25)			2: 12	0.85 (0.19–3.85)			3: 9	3.14 (0.68–14.40)				
	IMD 4–5	56: 105	3.21 (2.30–4.47)			25: 29	4.90 (2.84–8.46)			18: 26	4.15 (2.25–7.68)			9: 33	1.83 (0.75–4.46)				
Neurological symptoms	IMD 1–2	3072: 2602	9.44 (8.86–10.06)	0.7402	~1	2154: 666	22.37 (20.38–24.57)	0.7392	~1	790: 841	4.91 (4.43–5.44)	0.6662	~1	666: 882	4.25 (3.74–4.83)	0.9966	~1		
	IMD 3	1347: 1202	9.86 (8.98–10.84)			964: 329	22.02 (19.23–25.21)			340: 419	4.52 (3.89–5.26)			280: 407	4.29 (3.54–5.19)				
	IMD 4–5	2245: 2292	9.64 (8.98–10.35)			1630: 654	21.21 (19.21–23.42)			577: 756	4.86 (4.33–5.45)			487: 795	4.25 (3.69–4.91)				
Pain symptoms	IMD 1–2	2933: 7195	2.63 (2.49–2.78)	0.1584	~1	1324: 1996	3.61 (3.35–3.90)	0.0037*	0.2212	1250: 2412	2.73 (2.53–2.94)	0.7548	~1	1186: 2938	2.31 (2.12–2.53)	0.0475*	~1		
	IMD 3	1353: 3405	2.88 (2.65–3.13)			635: 858	4.43 (3.95–4.96)			560: 1204	2.65 (2.37–2.96)			570: 1439	2.77 (2.43–3.14)				
	IMD 4–5	2229: 6502	2.77 (2.60–2.94)			1099: 1786	4.20 (3.86–4.56)			981: 2409	2.62 (2.41–2.84)			992: 2849	2.61 (2.37–2.87)				
Psychiatric symptoms	IMD 1–2	2241: 5798	2.09 (1.98–2.22)	0.0808	~1	933: 1469	3.08 (2.82–3.36)	0.0442*	~1	948: 1886	2.41 (2.21–2.62)	0.0477*	~1	928: 2354	2.12 (1.91–2.35)	0.0760	~1		
	IMD 3	1056: 2766	2.36 (2.16–2.57)			444: 716	3.35 (2.95–3.81)			468: 887	2.82 (2.50–3.18)			465: 1163	2.57 (2.22–2.97)				
	IMD 4–5	1879: 6053	2.21 (2.07–2.36)			842: 1478	3.62 (3.30–3.97)			867: 1987	2.73 (2.50–2.98)			834: 2644	2.38 (2.14–2.64)				

**TABLE 5:** Results from a univariate logistic regression model examining the interaction between pre‐diagnostic symptoms and rural/urban location on the risk of MS.

Symptoms	Location	All pre‐diagnostic years				0–2 years prior diagnosis				2–5 years prior diagnosis				5–10 years prior diagnosis			
		Case: Control	Unadjusted OR (95% CI)	*p*‐int	Bonferroni corrected *p*‐int	Case: Control	Unadjusted OR (95% CI)	*p*‐int	Bonferroni corrected *p*‐int	Case: Control	Unadjusted OR (95% CI)	*p*‐int	Bonferroni corrected *p*‐int	Case: Control	Unadjusted OR (95% CI)	*p*‐int	Bonferroni corrected *p*‐int
Autonomic symptoms	Rural	1031: 2809	2.02 (1.84–2.20)	0.0014*	0.0273*	424: 956	2.18 (1.92–2.47)	< 0.0001*	0.0009*	445: 1122	1.93 (1.71–2.17)	0.0589	~1	427: 1103	2.08 (1.80–2.40)	0.4055	~1
	Urban	4720: 13863	2.37 (2.27–2.47)			2085: 4418	2.94 (2.78–3.12)			1977: 5330	2.19 (2.07–2.32)			1974: 5859	2.22 (2.08–2.38)		
Cognitive symptoms	Rural	15: 35	2.03 (1.09–3.76)	0.1997	~1	6: 15	1.67 (0.64–4.36)	0.0048*	0.2899	1: 12	0.42 (0.05–3.28)	0.0489*	~1	4: 8	3.22 (0.75–13.85)	0.6197	~1
	Urban	115: 209	3.10 (2.45–3.91)			68: 57	6.79 (4.73–9.74)			25: 57	2.39 (1.48–3.86)			19: 60	2.14 (1.13–4.07)		
Neurological symptoms	Rural	1215: 1070	8.23 (7.45–9.09)	0.0010*	0.0197*	877: 276	20.43 (17.66–23.64)	0.3479	~1	301: 355	4.04 (3.44–4.76)	0.0221*	~1	261: 340	3.95 (3.22–4.85)	0.4336	~1
	Urban	5449: 5026	9.90 (9.45–10.37)			3871: 1373	22.07 (20.64–23.59)			1406: 1661	4.98 (4.62–5.37)			1172: 1744	4.33 (3.94–4.75)		
Pain symptoms	Rural	1146: 2801	2.35 (2.15–2.57)	0.0003*	0.0067*	519: 753	3.43 (3.04–3.88)	0.0116*	0.6986	476: 970	2.33 (2.07–2.63)	0.0191*	~1	482: 1137	2.14 (1.85–2.46)	0.0154*	0.9254
	Urban	5369: 14301	2.80 (2.69–2.92)			2539: 3887	4.08 (3.86–4.31)			2315: 5055	2.73 (2.59–2.88)			2266: 6089	2.58 (2.42–2.75)		
Psychiatric symptoms	Rural	917: 2381	1.92 (1.75–2.10)	0.0041*	0.0830	378: 636	2.67 (2.33–3.06)	0.0007*	0.0560	367: 740	2.22 (1.94–2.54)	0.0142*	0.8526	403: 940	2.22 (1.90–2.59)	0.6921	~1
	Urban	4259: 12236	2.22 (2.13–2.32)			1841: 3027	3.46 (3.25–3.68)			1916: 4020	2.67 (2.51–2.83)			1824: 5221	2.30 (2.14–2.47)		

### 3.4 Sensitivity Analysis

Sensitivity analyses did not materially change any of the findings described above (Tables S5 and S6, Figures S2–S4).

## 4 Discussion

Previous descriptions of a Multiple Sclerosis prodrome—the constellation of nonspecific symptoms occurring in the years preceding an MS diagnosis—have focused on populations of White ethnicity. Here, we show that this phenomenon is generalisable to people from diverse ethnic and socio‐economic backgrounds living in the UK. We show that patients with MS tend to experience a range of symptoms in the run‐up to their diagnosis at rates higher than healthy controls, regardless of their demographic background.

Importantly, we find evidence of prolonged pre‐diagnostic neurological features more clearly pointing towards MS in particular demographic groups. The apparent enhancement of the relationship between neurological and autonomic prodromal symptoms and subsequent MS in males, those from Black, Asian and mixed ethnic backgrounds and those living in urban populations is notable. All of these groups have lower prior odds of MS, meaning that an equivalent absolute risk increase associated with a specific prodromal feature would manifest as an increased odds ratio (i.e., a risk increase of 0.3% equates to a relative risk of 2 for a population with a background risk of 0.3%, but a relative risk of 3 for a population with a lower background risk of 0.15%). Another interpretation is that these groups are diagnosed later in disease or present more frequently prior to onward referral. In all cases, the absolute risk increase is moderate, and the direction of influence remains constant. Granular population‐level data would enable accurate estimation of absolute effects, but these data were not available as part of this nested case–control study.

The disconnect in deprivation‐level measurements, where a significant relationship between IMD and MS exists when personal‐level (i.e., home address) IMD is used, but with no significant relationship seen when IMD at general practice level (i.e., address of general practice), is worthy of discussion. This finding highlights the importance of personal characteristics in terms of ability to navigate healthcare, which in turn affects an individual's ability to ‘obtain’ an MS diagnosis, a finding that has been replicated when examining the role of social determinants of health in outcomes subsequent to an MS diagnosis [15]. Our results indicate that this is more important than local and environmental factors, which are reflected in practice‐level IMD, again supported by recent literature in post‐diagnostic cohorts [15]. Whilst it is known that IMD is a relatively crude measure of overall social determinants of health and healthcare access, despite apparent acceptability to patients and healthcare providers, more accurate measures are poorly recorded in routinely collected healthcare datasets [27].

Recent algorithmic work has highlighted the importance of accurate case finding within routinely collected health datasets [28]. Our consideration of diagnostic codes across primary and secondary care data, requiring a ‘definite’ MS code along with supportive evidence, follows a similar approach to this gold standard. However, we cannot assume that all cases and controls were correctly identified as such, a potential weakness of our work. Further, due to national commissioning and centralised prescription of MS disease modifying therapies, and the relatively poor data depth within HES outpatient data, we were unable to access any data regarding diagnostic processes and potential delays, DMT access, and disability outcomes.

It is important to note that data missingness related to demographic factors may have influenced these findings. Both recorded sex and deprivation status had almost complete ascertainment, whereas ethnicity was subject to substantial missingness. The unequal distribution of missing ethnicity data (2.9% in MS and 25.5% in controls) is important to note, and likely reflects the differential opportunities for recording of ethnicity during healthcare contacts; with increasing healthcare contacts, particularly across secondary care, the chance of ethnicity being recorded increases. Comparison with national census data suggests that most of those with missing ethnicity are likely to be of South Asian or White ethnic backgrounds [29]. A further limitation of this study is the necessarily arbitrary nature of the ethnicity labels used. Whilst these limitations may have influenced some of our findings, the consistent direction of effect across ethnic and socio‐economic backgrounds is notable. In addition, whilst we use broad ethnicity categories to demonstrate that the MS prodrome applies across diverse populations, it is important to emphasise that these categories are not biological constructs nor do they refer to homogenous groups. Despite these limitations, our results carry implications for efforts aiming to detect MS at the earliest possible disease stage.

We relied on symptom reporting according to primary care coding. However, it is important to note that inaccuracies can occur in symptom reporting in primary care. Under‐reporting of symptoms can occur where symptoms may not be mentioned or asked for during consultations, such as unproblematic or unrelated symptoms. Furthermore, there is potential for bias in recording across different ethnicities and sexes due to differing socio‐linguistic contexts. Some symptoms may be more frequently asked than others, and this may vary across demographic groups, either according to measured or unmeasured factors, leading to misclassification bias. Our work, furthermore, could not account for variables that may represent possible confounders, such as physical activity or education.

Despite these limitations, our study provides for the first time a rigorous evaluation of the consistency of symptoms recorded prior to an MS diagnosis. The significant differences in association require further evaluation in order to understand whether these are related to baseline prevalence or a true difference in size of effect. However, our work shows that any future efforts to identify people at the earliest stages of MS using a prodromal framework are robust to selected social determinants of health including sex, ethnicity and level of deprivation.

## Author Contributions

**R.D**. conceived the idea of this study. **P.T**. and **B.M.J**. designed statistical analysis with input from **J.B**. **P.T.** performed all analysis, with code subsequently validated by **B.M.J**., **P.T**. drafted the initial manuscript with input from **R.D**. All authors then provided important intellectual input into the final manuscript.

## Disclosure

R.D. has received honoraria for speaking and/or travelling from Biogen, Merck, Teva, Roche, Janssen and Sanofi. She served on advisory boards for Roche, Biogen, Janssen and Merck and study steering committees for Roche. All honoraria are paid into an institutional account and used to support research and training. She has received grant support from Biogen, Merck and Celgene. P.T., J.B. and B.J. report no disclosures.

## Ethics Statement

Data used in this study were obtained from CPRD and analysed under approved protocol 21_000677. Ethical approval was provided by the CPRD Independent Scientific Advisory Committee.

## Conflicts of Interest

The authors declare no conflicts of interest.

## Supporting information


**Figure S1:** Frequency of multiple sclerosis diagnosis per patient (A) and combination of neurology referral and multiple sclerosis diagnosis per patient (B).
**Figure S2:** Comparing the interaction between ethnicity and pre‐diagnostic symptoms by periods of different MS diagnosis criteria (unadjusted model).
**Figure S3:** Comparing the interaction between gender and pre‐diagnostic symptoms by MS diagnosis criteria (unadjusted model).
**Figure S4:** Prodromal symptoms according to codes used to define MS.

**Table S1:** Read codes used to define multiple sclerosis and ethnicity.

**Table S2:** Codes used to define and categorise prodromal symptoms.

**Table S3:** (a) Numbers of MS cases and controls by detailed ethnicity label; (b) Demographic and clinical characteristics of patients by MS status according to duration of retrospective data availability.

**Table S4:** Missing data table, characteristics for those who have at least 10 years vs. those who only have 5 years prior index date.
**Table S5:** Results from a multivariable logistic regression (adjusted) model examining the interaction between pre‐diagnostic symptoms and demographic subgroups on the risk.
**Table S6:** Comparing the interaction between demographic backgrounds and pre‐diagnostic symptoms by periods of different MS diagnosis criteria.
**Table S7:** Prodromal symptoms according to codes used to define MS.

## Data Availability

CPRD data are available via application to CPRD's Research Data Governance (RDG) Process (see https://www.cprd.com/research‐applications).
