# Supplementary material for: Pre‐Diagnostic Features of Multiple Sclerosis in a Diverse UK Cohort: A Nested Case–Control Study
Source: Ann Clin Transl Neurol. 2025 Sep 24;13(1):71–84. doi: 10.1002/acn3.70175 (PMC12790163; doi:10.1002/acn3.70175)

**Supplementary figure 1:** Frequency of multiple sclerosis diagnosis per patient (**A**) and combination of neurology referral and multiple sclerosis diagnosis per patient (**B**)


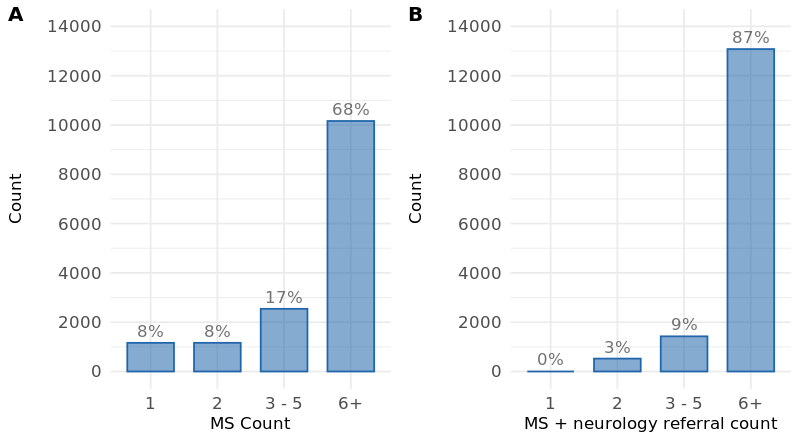


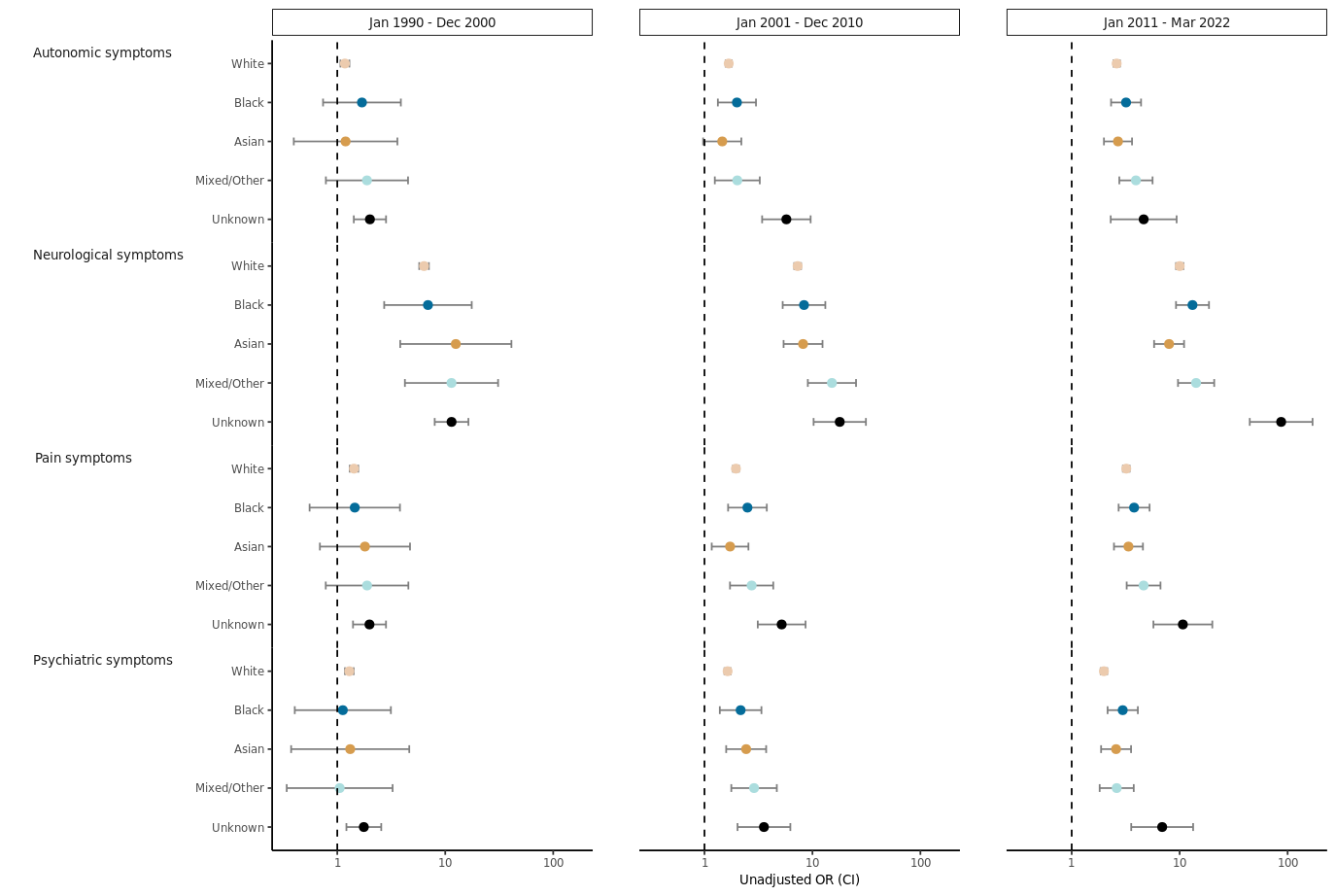
**Supplementary figure 2:** Comparing the interaction between Ethnicity and pre-diagnostic symptoms by periods of different MS diagnosis criteria (unadjusted model).

**Supplementary figure 3:** Comparing the interaction between gender and pre-diagnostic symptoms by periods of different MS diagnosis criteria (unadjusted model).


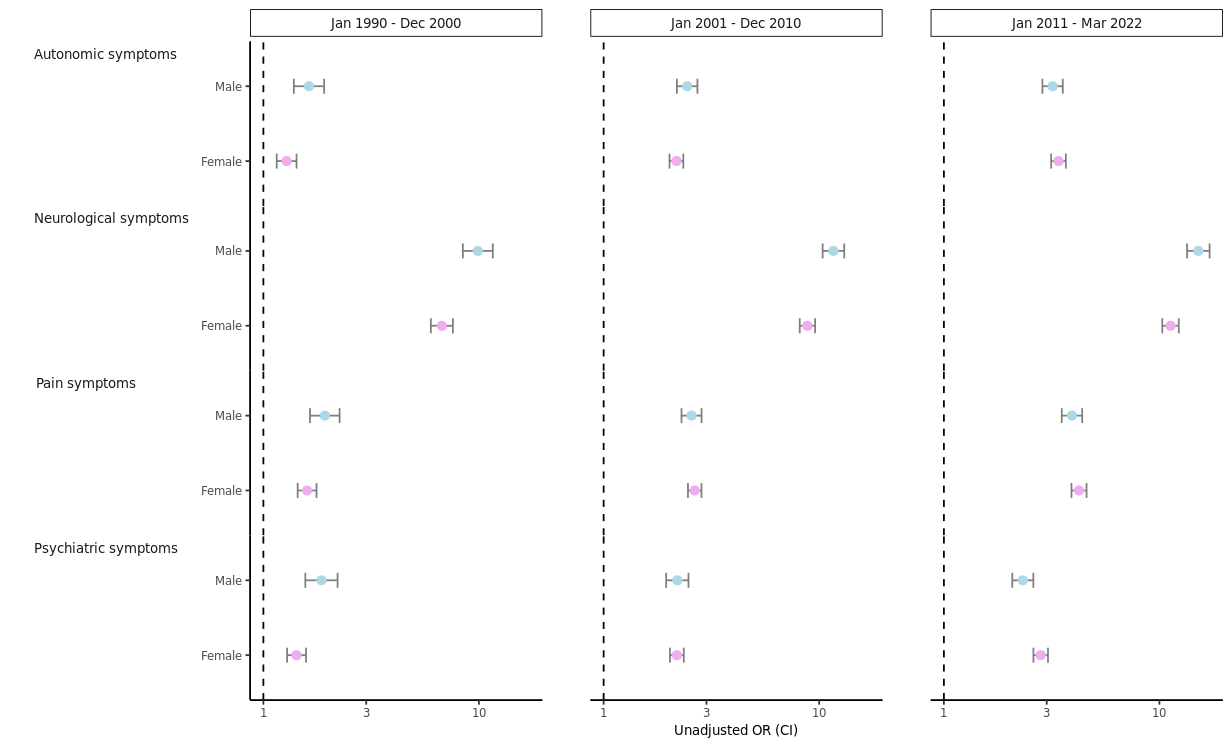


**Supplementary figure 4:** Prodromal symptoms according to codes used to define MS


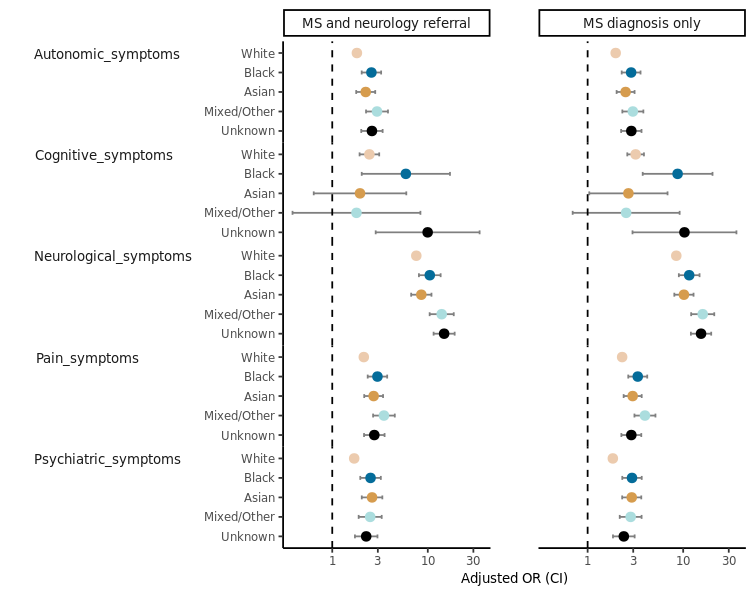

Supplement: Supplementary file 1 — Figure S1: Frequency of multiple sclerosis diagnosis per patient (A) and combination of neurology referral and multiple sclerosis diagnosis per patient (B). Figure S2: Comparing the interaction between ethnicity and pre‐diagnostic symptoms by periods of different MS diagnosis criteria (unadjusted model). Figure S3: Comparing the interaction between gender and pre‐diagnostic symptoms by MS diagnosis criteria (unadjusted model). Figure S4: Prodromal symptoms according to codes used to define MS. [file ACN3-13-71-s005.docx]
