# Supplementary material for: Pre‐Diagnostic Features of Multiple Sclerosis in a Diverse UK Cohort: A Nested Case–Control Study
Source: Ann Clin Transl Neurol. 2025 Sep 24;13(1):71–84. doi: 10.1002/acn3.70175 (PMC12790163; doi:10.1002/acn3.70175)
Supplement: Supplementary file 2 — Table S1: Read codes used to define multiple sclerosis and ethnicity. [file ACN3-13-71-s004.docx]

Supplementary table 1: Read codes used to define multiple sclerosis and ethnicity

| **1a: Ethnicity recording in HES APC, HES OP, and HES A&E.** Individual ethnicities are specified by the patient and grouped accordingly into the 5 categories. | |
| --- | --- |
| Grouped Ethnicity | HES Ethnicity categories |
| White | 0 = White |
|  | A = British (White) |
|  | B = Irish (White) |
|  | C = Any other White background |
| Black | 1 = Black – Caribbean |
|  | 2 = Black – African |
|  | 3 = Black – Other |
|  | M = Caribbean (Black or Black British) |
|  | N = African (Black or Black British) |
|  | P = Any other Black background |
| Asian | 4 = Indian |
|  | 5 = Pakistani |
|  | 6 = Bangladeshi |
|  | H = Indian (Asian or Asian British) |
|  | J = Pakistani (Asian or Asian British) |
|  | K = Bangladeshi (Asian or Asian British) |
|  | L = Any other Asian background |
| Mixed/Other | 7 = Chinese |
|  | 8 = Any other ethnic group |
|  | D = White and Black Caribbean (Mixed) |
|  | E = White and Black African (Mixed) |
|  | F = White and Asian (Mixed) |
|  | G = Any other Mixed background |
|  | R = Chinese (other ethnic group) |
|  | S = Any other ethnic group |
| Unknown | X/Z/99 = Not stated |

| **1b: Ethnicity Recording in CPRD Aurum.** | | | | |
| --- | --- | --- | --- | --- |
| 5 Ethnic Categories | Ethnic sub categories | readcode | medcode | term decription |
| White | British | 9S1..00 | 285925010 | White |
|  |  | 9S10.00 | 459726019 | White British |
|  |  | 9S14.00 | 1780408016 | Other white British ethnic group |
|  |  | 9t00.00 | 1968051000006116 | White:Eng/Welsh/Scot/NI/Brit - England and Wales 2011 census |
|  |  | 9i0..00 | 158341000000117 | British or mixed British - ethnic category 2001 census |
|  |  | 9i00.00 | 1063981000000117 | White British - ethnic category 2001 census |
|  | Irish | 9SA9.00 | 285978014 | Irish (NMO) |
|  |  | 9S11.00 | 459727011 | White Irish |
|  |  | 9SI..00 | 459786016 | Irish traveller |
|  |  | 9t01.00 | 2484671000000118 | White: Irish - England and Wales ethnic category 2011 census |
|  |  | 9t22.00 | 2487361000000112 | White: Irish - Scotland ethnic category 2011 census |
|  |  | 9i1..00 | 141301000000110 | Irish - ethnic category 2001 census |
|  |  | 9i10.00 | 1064041000000111 | White Irish - ethnic category 2001 census |
|  | Other White | 9T8..00 | 133078012 | Portuguese |
|  |  | 9T11.00 | 286007012 | New Zealand European |
|  |  | 9T11.11 | 286008019 | Pakeha |
|  |  | 9T12.00 | 286009010 | Other European in New Zealand |
|  |  | 9S12.00 | 459728018 | White - ethnic group |
|  |  | 9S13.00 | 1780407014 | White Scottish |
|  |  | 9T2..00 | 850671000006119 | Gypsies |
|  |  | 9T4..00 | 1158211000000111 | Romanian |
|  |  | 9T5..00 | 1158301000000115 | Bulgarian |
|  |  | 9T6..00 | 1160331000000119 | Czech |
|  |  | 9T7..00 | 1551471000000116 | Slovak |
|  |  | 9t02.00 | 1968071000006114 | White: Gypsy/Irish Traveller - Eng+Wales eth cat 2011 census |
|  |  | 9t03.00 | 1968081000006112 | White: other White backgrd- Eng+Wales ethnic cat 2011 census |
|  |  | 9t11.00 | 1968251000006113 | Irish Traveller - Northern Ireland ethnic category 2011 census |
|  |  | 9t23.00 | 1968441000006112 | White: Gypsy or Irish Traveller - Scotland ethnic category 2011 census |
|  |  | 9t25.00 | 1968461000006111 | White: other White ethnic grp- Scotland ethnic cat 2011 cens |
|  |  | 9t10.00 | 2486161000000112 | White - Northern Ireland ethnic category 2011 census |
|  |  | 9t20.00 | 2487281000000112 | White: Scottish - Scotland ethnic category 2011 census |
|  |  | 9t21.00 | 2487321000000116 | White: other British - Scotland ethnic category 2011 census |
|  |  | 9t24.00 | 2487481000000113 | White: Polish - Scotland ethnic category 2011 census |
|  |  | 9TC4 | 2615361000000115 | Slovak Roma |
|  |  | 9TC1 | 2615461000000111 | Czech Roma |
|  |  | 9TC5 | 2615531000000117 | Hungarian Roma |
|  |  | 9TC2 | 2615571000000115 | Polish Roma |
|  |  | 9TC3 | 2615611000000112 | Romanian Roma |
|  |  | 9TC0 | 2615691000000115 | Bulgarian Roma |
|  |  | 9TC | 2645811000000115 | Roma ethnic group |
|  |  | 9i2C.00 | 138171000000114 | Irish Traveller - ethnic category 2001 census |
|  |  | 9i2D.00 | 138181000000111 | Traveller - ethnic category 2001 census |
|  |  | 9i2E.00 | 138191000000113 | Gypsy/Romany - ethnic category 2001 census |
|  |  | 9i2F.00 | 138201000000110 | Polish - ethnic category 2001 census |
|  |  | 9i2K.00 | 138231000000116 | Albanian - ethnic category 2001 census |
|  |  | 9i2..00 | 141311000000112 | Other White background - ethnic category 2001 census |
|  |  | 9i21.00 | 141431000000111 | Scottish - ethnic category 2001 census |
|  |  | 9i22.00 | 141441000000119 | Welsh - ethnic category 2001 census |
|  |  | 9i24.00 | 141451000000116 | Northern Irish - ethnic category 2001 census |
|  |  | 9i23.00 | 141461000000118 | Cornish - ethnic category 2001 census |
|  |  | 9i26.00 | 141661000000115 | Cypriot (part not stated) - ethnic category 2001 census |
|  |  | 9i25.00 | 142691000000116 | Ulster Scots - ethnic category 2001 census |
|  |  | 9i27.00 | 142701000000116 | Greek - ethnic category 2001 census |
|  |  | 9i28.00 | 142711000000119 | Greek Cypriot - ethnic category 2001 census |
|  |  | 9i2A.00 | 142721000000113 | Turkish Cypriot - ethnic category 2001 census |
|  |  | 9i2J.00 | 142741000000118 | Kosovan - ethnic category 2001 census |
|  |  | 9i2L.00 | 142751000000115 | Bosnian - ethnic category 2001 census |
|  |  | 9i2M.00 | 142761000000117 | Croatian - ethnic category 2001 census |
|  |  | 9i2Q.00 | 142781000000114 | Mixed Irish and other White - ethnic category 2001 census |
|  |  | 9i2S.00 | 142791000000111 | Other mixed White - ethnic category 2001 census |
|  |  | 9i29.00 | 156921000000110 | Turkish - ethnic category 2001 census |
|  |  | 9i20.00 | 157281000000117 | English - ethnic category 2001 census |
|  |  | 9i2N.00 | 157991000000110 | Serbian - ethnic category 2001 census |
|  |  | 9i2B.00 | 158481000000115 | Italian - ethnic category 2001 census |
|  |  | 9i2G.00 | 937301000006110 | Baltic Estonian/Latvian/Lithuanian - ethn categ 2001 census |
|  |  | 9i2H.00 | 937311000006113 | Commonwealth (Russian) Indep States - ethn categ 2001 census |
|  |  | 9i2P.00 | 937371000006116 | Other republics former Yugoslavia - ethnic categ 2001 census |
|  |  | 9i2R.00 | 937391000006115 | Oth White European/European unsp/Mixed European 2001 census |
|  |  | 9i2T.00 | 937411000006115 | Other White or White unspecified - ethnic category 2001 census |
| Black | Caribbean | 9S2..00 | 514611000006111 | Black Caribbean |
|  |  | 9t2E.00 | 1968551000006110 | Carib/Black: Caribbean/Carib Scot/Carib Brit- Scotland 2011 |
|  |  | 9iB..00 | 154401000000118 | Caribbean - ethnic category 2001 census |
|  | African | 9S3..00 | 30683015 | Black African |
|  |  | 9SA5.00 | 285971015 | Other African countries (NMO) |
|  |  | 9t2C.00 | 1968531000006115 | African: African/African Scot/African Brit - Scotland 2011 |
|  |  | 9t2D.00 | 1968541000006113 | African: any other African - Scotland ethnic cat 2011 census |
|  |  | 9iC..00 | 141391000000115 | African - ethnic category 2001 census |
|  | Other Black | 9S42.11 | 285930014 | Black Caribbean |
|  |  | 9S4..00 | 285931013 | Black, other, non-mixed origin |
|  |  | 9S41.00 | 285932018 | Black British |
|  |  | 9S44.00 | 285943014 | Black - other African country |
|  |  | 9S46.00 | 285948017 | Black Indian sub-continent |
|  |  | 9S47.00 | 285949013 | Black - other Asian |
|  |  | 9S48.00 | 285950013 | Black Black - other |
|  |  | 9S5..00 | 285951012 | Black - other, mixed |
|  |  | 9S42.00 | 405064011 | Black Caribbean/W.I./Guyana |
|  |  | 9S43.00 | 405065012 | Black N African/Arab/Iranian |
|  |  | 9SA3.00 | 405067016 | Caribbean I./W.I./Guyana (NMO) |
|  |  | 9S43.11 | 411573013 | Black North African |
|  |  | 9S43.12 | 411574019 | Black Arab |
|  |  | 9S43.13 | 411575018 | Black Iranian |
|  |  | 9S45.11 | 411576017 | Black East African Asian |
|  |  | 9S45.12 | 411577014 | Black Indo-Caribbean |
|  |  | 9SA3.11 | 411578016 | Caribbean Island (NMO) |
|  |  | 9SA3.12 | 411579012 | West Indian (NMO) |
|  |  | 9SA3.13 | 411580010 | Guyana (NMO) |
|  |  | 9S42.12 | 453109012 | Black West Indian |
|  |  | 9S42.13 | 453110019 | Black Guyana |
|  |  | 9SG..00 | 459782019 | Other black ethnic group |
|  |  | 9S45.00 | 514651000006112 | Black East African Asian/Indo-Caribbean |
|  |  | 9t0D.00 | 1968181000006113 | Black/African/Carib/Black Brit: African- Eng+Wales 2011 cens |
|  |  | 9t0E.00 | 1968191000006111 | Black/African/Caribbn/Black Brit: Caribbean - Eng+Wales 2011 |
|  |  | 9t0F.00 | 1968201000006114 | Black/Afr/Carib/Black Brit: other Black- Eng+Wales 2011 cens |
|  |  | 9t1B.00 | 1968351000006119 | Black/Afri/Carib/Black Brit: African- NI eth cat 2011 census |
|  |  | 9t1C.00 | 1968361000006117 | Black/Afri/Carib/Black Brit: Caribbean- NI eth cat 2011 cens |
|  |  | 9t1D.00 | 1968371000006112 | Black/Afri/Carib/Black Brit: other - NI eth cat 2011 census |
|  |  | 9t2F.00 | 1968561000006112 | Carib/Black: Black/Black Scot/Black Brit- Scotland 2011 cens |
|  |  | 9t2G.00 | 1968571000006117 | Carib/Black: any other Black/Caribbean grp - Scotland 2011 |
|  |  | 9iD0.00 | 141591000000113 | Somali - ethnic category 2001 census |
|  |  | 9iD1.00 | 141601000000119 | Nigerian - ethnic category 2001 census |
|  |  | 9iD2.00 | 157311000000119 | Black British - ethnic category 2001 census |
|  |  | 9iD..00 | 158351000000119 | Other Black background - ethnic category 2001 census |
|  |  | 9iD3.00 | 158371000000111 | Mixed Black - ethnic category 2001 census |
|  |  | 9iD4.00 | 937731000006115 | Other Black or Black unspecified - ethnic category 2001 census |
| Asian | Indian | 9S6..00 | 285954016 | Indian |
|  |  | 9T1D.00 | 781081000006113 | Indian |
|  |  | 9t08.00 | 1968131000006112 | Asian/Asian Brit: Indian - Eng+Wales ethnic cat 2011 census |
|  |  | 9t16.00 | 1968301000006118 | Asian or Asian British: Indian - NI ethnic cat 2011 census |
|  |  | 9t28.00 | 1968491000006115 | Asian: Indian, Indian Scot/Indian Brit- Scotland 2011 census |
|  |  | 9i7..00 | 157271000000119 | Indian or British Indian - ethnic category 2001 census |
|  | Pakistani | 9S7..00 | 285955015 | Pakistani |
|  |  | 9t09.00 | 1968141000006119 | Asian/Asian British:Pakistani- Eng+Wales eth cat 2011 census |
|  |  | 9t17.00 | 1968311000006115 | Asian/Asian British: Pakistani - NI ethnic cat 2011 census |
|  |  | 9t27.00 | 1968481000006118 | Asian: Pakistani/Pakistani Scot/Pakistani Brit- Scot 2011 |
|  |  | 9i8..00 | 141361000000114 | Pakistani or British Pakistani - ethnic category 2001 census |
|  | Bangladeshi | 9S8..00 | 285956019 | Bangladeshi |
|  |  | 9t0A.00 | 1968151000006117 | Asian/Asian Brit: Bangladeshi- Eng+Wales eth cat 2011 census |
|  |  | 9t18.00 | 1968321000006111 | Asian/Asian British: Bangladeshi - NI ethnic cat 2011 census |
|  |  | 9t29.00 | 1968501000006111 | Bangladeshi, Bangladeshi Scot or Bangladeshi Brit- Scot 2011 |
|  |  | 9i9..00 | 937541000006115 | Bangladeshi or British Bangladeshi - ethn categ 2001 census |
|  | Other Asian | 9SA7.00 | 285976013 | Indian sub-continent (NMO) |
|  |  | 9SA8.00 | 285977016 | Other Asian (NMO) |
|  |  | 9T1B.00 | 286018012 | South East Asian |
|  |  | 9T1E.00 | 286020010 | Asian - ethnic group |
|  |  | 9SA6.00 | 405069018 | E Afric Asian/Indo-Carib (NMO) |
|  |  | 9SA6.11 | 411583012 | East African Asian (NMO) |
|  |  | 9SA6.12 | 411584018 | Indo-Caribbean (NMO) |
|  |  | 9SH..00 | 459784018 | Other Asian ethnic group |
|  |  | 9T9..00 | 1572831000000110 | Nepali |
|  |  | 9t0C.00 | 1968171000006110 | Asian/Asian Brit: other Asian- Eng+Wales eth cat 2011 census |
|  |  | 9t1A.00 | 1968341000006116 | Asian/Asian British: other Asian - NI ethnic cat 2011 census |
|  |  | 9t2B.00 | 1968521000006118 | Asian: other Asian group - Scotland ethnic cat 2011 census |
|  |  | 9iA4.00 | 136081000000111 | Sri Lankan - ethnic category 2001 census |
|  |  | 9iA..00 | 141381000000117 | Other Asian background - ethnic category 2001 census |
|  |  | 9iA9.00 | 141511000000116 | Mixed Asian - ethnic category 2001 census |
|  |  | 9iA1.00 | 141521000000110 | Punjabi - ethnic category 2001 census |
|  |  | 9iA2.00 | 141531000000112 | Kashmiri - ethnic category 2001 census |
|  |  | 9iA3.00 | 141541000000115 | East African Asian - ethnic category 2001 census |
|  |  | 9iA5.00 | 141551000000117 | Tamil - ethnic category 2001 census |
|  |  | 9iA8.00 | 141561000000119 | British Asian - ethnic category 2001 census |
|  |  | 9iA7.00 | 141571000000114 | Caribbean Asian - ethnic category 2001 census |
|  |  | 9iA6.00 | 157301000000116 | Sinhalese - ethnic category 2001 census |
|  |  | 9iAA.00 | 937651000006117 | Other Asian or Asian unspecified - ethnic category 2001 census |
| Mixed / Other | Mixed - White and Black | 9SB6.00 | 460154012 | Black African and White |
|  |  | 9t05.00 | 1968101000006116 | Mixed: White+Black African - Eng+Wales eth cat 2011 census |
|  |  | 9t13.00 | 1968271000006115 | Mixed: White and Black African - NI ethnic cat 2011 census |
|  |  | 9i4..00 | 141331000000116 | White and Black African - ethnic category 2001 census |
|  |  | 9SB5.00 | 460153018 | Black Caribbean and White |
|  |  | 9t04.00 | 1968091000006110 | Mixed: White+Black Caribbean - Eng+Wales eth cat 2011 census |
|  |  | 9t12.00 | 1968261000006110 | Mixed: White and Black Caribbean - NI ethnic cat 2011 census |
|  |  | 9i3..00 | 141321000000118 | White and Black Caribbean - ethnic category 2001 census |
|  | Mixed - White and Asian | 9SB2.00 | 285991010 | Other ethnic, Asian/White orig |
|  |  | 9t06.00 | 1968111000006118 | Mixed: White+Asian - Eng+Wales ethnic category 2011 census |
|  |  | 9t14.00 | 1968281000006117 | Mixed: White and Asian - NI ethnic category 2011 census |
|  |  | 9i5..00 | 141341000000113 | White and Asian - ethnic category 2001 census |
|  | Mixed Other | 9S51.00 | 285952017 | Other Black - Black/White orig |
|  |  | 9S52.00 | 285953010 | Other Black - Black/Asian orig |
|  |  | 9SB..00 | 285989019 | Other ethnic, mixed origin |
|  |  | 9SB1.00 | 285990011 | Other ethnic, Black/White orig |
|  |  | 9SB3.00 | 285992015 | Other ethnic, mixed white orig |
|  |  | 9SB4.00 | 285993013 | Other ethnic, other mixed orig |
|  |  | 9t07.00 | 1968121000006114 | Mixed: other Mixed/multiple backgrd - Eng+Wales 2011 census |
|  |  | 9t15.00 | 1968291000006119 | Mixed: other Mixed/multiple ethnic backgrd - NI 2011 census |
|  |  | 9t26.00 | 1968471000006116 | Mixed/multiple ethnic grps: any- Scot ethnic cat 2011 census |
|  |  | 9i6..00 | 141351000000111 | Other Mixed background - ethnic category 2001 census |
|  |  | 9i60.00 | 141471000000113 | Black and Asian - ethnic category 2001 census |
|  |  | 9i61.00 | 141481000000110 | Black and Chinese - ethnic category 2001 census |
|  |  | 9i63.00 | 141491000000112 | Chinese and White - ethnic category 2001 census |
|  |  | 9i62.00 | 157291000000115 | Black and White - ethnic category 2001 census |
|  |  | 9i64.00 | 158361000000116 | Asian and Chinese - ethnic category 2001 census |
|  |  | 9i65.00 | 937511000006119 | Other Mixed or Mixed unspecified - ethnic category 2001 census |
|  | Chinese | 9S9..00 | 56590016 | Chinese |
|  |  | 9T1C.00 | 550541000006110 | Chinese |
|  |  | 9t0B.00 | 1968161000006115 | Asian/Asian Brit: Chinese - Eng+Wales ethnic cat 2011 census |
|  |  | 9t2A.00 | 1968511000006114 | Asian: Chinese - Scotland ethnic category 2011 census |
|  |  | 9iE..00 | 141401000000117 | Chinese - ethnic category 2001 census |
|  | Other ethnic group | 9SA..00 | 285958018 | Other ethnic non-mixed (NMO) |
|  |  | 9SA1.00 | 285959014 | Brit. ethnic minor. spec.(NMO) |
|  |  | 9SA2.00 | 285960016 | Brit. ethnic minor. unsp (NMO) |
|  |  | 9SAC.00 | 285987017 | Other European (NMO) |
|  |  | 9SAD.00 | 285988010 | Other ethnic NEC (NMO) |
|  |  | 9T1..00 | 286006015 | New Zealand ethnic groups |
|  |  | 9T13.00 | 286012013 | New Zealand Maori |
|  |  | 9T15.00 | 286013015 | Cook Island Maori |
|  |  | 9T17.00 | 286014014 | Niuean |
|  |  | 9T18.00 | 286015010 | Tokelauan |
|  |  | 9T1A.00 | 286017019 | Other Pacific ethnic group |
|  |  | 9T1Y.00 | 286021014 | Other New Zealand ethnic group |
|  |  | 9SA4.00 | 405068014 | N African Arab/Iranian (NMO) |
|  |  | 9SAA.00 | 405070017 | Greek/Greek Cypriot (NMO) |
|  |  | 9SAB.00 | 405071018 | Turkish/Turkish Cypriot (NMO) |
|  |  | 9SA4.11 | 411581014 | North African Arab (NMO) |
|  |  | 9SA4.12 | 411582019 | Iranian (NMO) |
|  |  | 9SAA.11 | 411594011 | Greek (NMO) |
|  |  | 9SAA.12 | 411595012 | Greek Cypriot (NMO) |
|  |  | 9SAB.11 | 411596013 | Turkish (NMO) |
|  |  | 9SAB.12 | 411597016 | Turkish Cypriot (NMO) |
|  |  | 9SC..00 | 456650013 | Vietnamese |
|  |  | 9SJ..00 | 459785017 | Ethnic group |
|  |  | 9T19.00 | 501416013 | Fijian |
|  |  | 9T16.00 | 504723011 | Tongan |
|  |  | 9T14.00 | 507015012 | Samoan |
|  |  | 9T3..00 | 523591000000116 | Yemeni |
|  |  | 9t0G.00 | 1968211000006112 | Other ethnic group: Arab - Eng+Wales ethnic cat 2011 census |
|  |  | 9t0H.00 | 1968221000006116 | Other ethnic: any other grp - Eng+Wales eth cat 2011 census |
|  |  | 9t19.00 | 1968331000006114 | Asian/Asian British: Chinese - NI ethnic cat 2011 census |
|  |  | 9t1E.00 | 1968381000006110 | Other ethnic group: Arab - NI ethnic category 2011 census |
|  |  | 9t1F.00 | 1968391000006113 | Other ethnic group: any other grp- NI ethnic cat 2011 census |
|  |  | 9t2H.00 | 1968581000006119 | Other ethnic grp: Arab/Arab Scot/Arab British- Scotland 2011 |
|  |  | 9t2J.00 | 1968591000006116 | Other ethnic grp: any other ethnic grp- Scotland 2011 census |
|  |  | 9iF6.00 | 138241000000113 | Jewish - ethnic category 2001 census |
|  |  | 9iF9.00 | 138251000000111 | Arab - ethnic category 2001 census |
|  |  | 9iFD.00 | 138261000000114 | Iranian - ethnic category 2001 census |
|  |  | 9iFH.00 | 138271000000119 | South and Central American - ethnic category 2001 census |
|  |  | 9iF7.00 | 138281000000117 | Muslim - ethnic category 2001 census |
|  |  | 9iF..00 | 141411000000115 | Other - ethnic category 2001 census |
|  |  | 9iF0.00 | 141621000000111 | Vietnamese - ethnic category 2001 census |
|  |  | 9iF1.00 | 141631000000113 | Japanese - ethnic category 2001 census |
|  |  | 9iF2.00 | 141641000000116 | Filipino - ethnic category 2001 census |
|  |  | 9iF3.00 | 141651000000118 | Malaysian - ethnic category 2001 census |
|  |  | 9iFA.00 | 142811000000112 | North African - ethnic category 2001 census |
|  |  | 9iFC.00 | 142831000000116 | Israeli - ethnic category 2001 census |
|  |  | 9iFE.00 | 142841000000113 | Kurdish - ethnic category 2001 census |
|  |  | 9iFF.00 | 142851000000111 | Moroccan - ethnic category 2001 census |
|  |  | 9iFG.00 | 142861000000114 | Latin American - ethnic category 2001 census |
|  |  | 9iF4.00 | 142881000000117 | Buddhist - ethnic category 2001 census |
|  |  | 9iF8.00 | 142891000000115 | Sikh - ethnic category 2001 census |
|  |  | 9iFK.00 | 142901000000119 | Any other group - ethnic category 2001 census |
|  |  | 9iF5.00 | 157351000000115 | Hindu - ethnic category 2001 census |
|  |  | 9iFB.00 | 937871000006114 | Mid East (excl Israeli, Iranian & Arab) - eth cat 2001 cens |
|  |  | 9iFJ.00 | 937941000006111 | Mauritian/Seychellois/Maldivian/St Helena eth cat 2001census |

1c: Multiple sclerosis medcode lists in CPRD Aurum

| Confidence | Term | MedCodeId |
| --- | --- | --- |
| Definite | Multiple sclerosis NOS | 297181019 |
| Definite | Secondary progressive multiple sclerosis | 2674605012 |
| Definite | Relapsing remitting multiple sclerosis | 1682241000006111 |
| Definite | Primary progressive multiple sclerosis | 2692565012 |
| Definite | Malignant multiple sclerosis | 641301000000114 |
| Definite | Benign multiple sclerosis | 641211000000118 |
| Definite | Exacerbation of multiple sclerosis | 297180018 |
| Definite | Generalised multiple sclerosis | 297179016 |
| Definite | Multiple sclerosis of the spinal cord | 297177019 |
| Definite | Multiple sclerosis of the brain stem | 695191000006119 |
| Definite | Multiple sclerosis | 41398015 |
| Definite | Multiple sclerosis monitoring third letter | 1675051000000110 |
| Definite | Multiple sclerosis monitoring telephone invitation | 1674971000000118 |
| Definite | Multiple sclerosis monitoring second letter | 1674661000000118 |
| Definite | Multiple sclerosis monitoring first letter | 1674381000000115 |
| Definite | Multiple sclerosis monitoring administration | 1674121000000119 |
| Definite | Specialised services for patients with multiple sclerosis enhanced services administration | 979461000006111 |
| Definite | Multiple sclerosis review declined | 1148911000000119 |
| Definite | Referral to community multiple sclerosis team | 1757431000000113 |
| Definite | Multiple sclerosis care plan agreed | 300141000000119 |
| Definite | Management of multiple sclerosis in palliative phase | 699911000000117 |
| Definite | Management of multiple sclerosis in progressive disability phase | 1679791000006113 |
| Definite | Management of multiple sclerosis in stable disability phase | 699791000000111 |
| Definite | Management of multiple sclerosis in early disease phase | 699731000000110 |
| Definite | Management of multiple sclerosis in onset phase | 699671000000115 |
| Definite | Multiple sclerosis multidisciplinary review | 315671000000110 |
| Definite | Multiple sclerosis review | 299241000000110 |
| Definite | [RFC] Multiple sclerosis | 908811000006112 |
| Definite | [RFC] Multiple sclerosis | 905781000006118 |
| Definite | Referral by Multiple Sclerosis nurse specialist | 1863341000006118 |
| Definite | Multiple sclerosis – personal health plan | 983261000006119 |
| Definite | Multiple sclerosis questionnaire completed | 1855101000006113 |
| Definite | Multiple sclerosis monitoring telephone invite | 1747861000006111 |
| Definite | Multiple sclerosis monitoring third letter | 1747851000006114 |
| Definite | Multiple sclerosis monitoring first letter | 1747831000006119 |
| Definite | Multiple sclerosis monitoring administration | 1747821000006117 |
| Definite | Multiple sclerosis – relapsing remitting | 983311000006114 |
| Definite | Multiple sclerosis – secondary progressive | 983301000006111 |
| Definite | Multiple sclerosis – primary progressive | 983291000006110 |
| Definite | Remittent-progressive multiple sclerosis | 5006921000006118 |
| Definite | Progressive relapsing multiple sclerosis | 7861151000006116 |
| Definite | Multiple sclerosis clinic | 7567771000006118 |
| Definite | Multiple sclerosis (MS) primary progressive | 7092351000006112 |
| Definite | Multiple sclerosis (MS) relapsing remitting | 7058051000006116 |
| Definite | Multiple sclerosis (MS) secondary progressive | 7045281000006112 |
| Definite | Multiple sclerosis (MS) chronic/progressive | 5006911000006114 |
| Definite | Multiple sclerosis of the brainstem | 4769281000006110 |
| Definite | MS – Multiple sclerosis | 2894411000006110 |
| Definite | Kurtzke multiple sclerosis rating scale | 5552111000006118 |
| Definite | Dementia associated with multiple sclerosis | 7510741000006111 |
| Definite | Chronic progressive multiple sclerosis | 5006901000006111 |
| Definite | Acute relapsing multiple sclerosis | 5006891000006112 |
| Possible | Varicella transverse myelitis | 344996014 |
| Possible | Transverse myelitis | 28148010 |
| Possible | Neuromyelitis optica | 41961013 |
| Possible | Devic’s disease | 41962018 |
| Possible | Acute disseminated demyelination, unspecified | 456401000006118 |
| Possible | Other specified central nervous system demyelinating disease | 297190014 |
| Possible | Marchiafava-Bignami disease | 1480894012 |
| Possible | Central pontine myelinolysis | 12276017 |
| Possible | Central demyelination of corpus callosum | 1491762016 |
| Possible | Subacute necrotising myelitis | 345221014 |
| Possible | Concentric sclerosis | 345223012 |
| Possible | Vanishing white matter disease | 1648061000000115 |
| Possible | Other specified central nervous system demyelination NOS | 297195016 |
| Possible | Demyelinating disease of central nervous system | 297196015 |
| Possible | Benign Demyelinating Disease | 883011000006117 |
| Possible | Guillain-Barre syndrome | 68321016 |
| Possible | Acute infective polyneuritis | 207847010 |
| Possible | Acute infective polyneuritis NOS | 297539017 |
| Possible | Miller-Fisher syndrome | 478396017 |
| Possible | Postinfectious polyneuritis | 211331000006117 |
| Probable | Clinically isolated syndrome | 2883049010 |
| Probable | Suspected multiple sclerosis | 2262931000000117 |
| Probable | Suspected multiple sclerosis | 1858501000006114 |
| Probable | Possible MS | 854741000006113 |
| Probable | Optic neuritis | 110903014 |
| Probable | Optic neuritis NOS | 298753017 |
| Probable | Unspecified optic neuritis | 298751015 |
| Probable | Transverse myelitis | 91711000006118 |
| Probable | Other central nervous system demyelinating diseases | 297182014 |
| Probable | Schilder’s disease | 82759010 |
| Probable | Balo’s concentric sclerosis | 2475876012 |
| Probable | EDSS – Expanded disability status scale | 5552141000006119 |

1d: neurology referral Medcode IDs.

| Term | MedCodeId |
| --- | --- |
| Neurological referral | 283605011 |
| Neurology referral | 904911000006110 |
| Referral to neurologist | 451827017 |
| Private referral to neurologist | 284115010 |
| Referral to neurology service | 4724261000006113 |
| Referral to paediatric neurologist | 449233010 |
| Referral to neurology special interest general practitioner | 408441000000116 |
| Neurology emergency hospital admission | 283538014 |
| Non-urgent neurology admission | 283578019 |
| Paediatric neurology referral | 904951000006111 |
| Referred to Neurology Service | 1775481000006110 |
| Neurology self-referral | 283853012 |
