# Supplementary material for: Pre‐Diagnostic Features of Multiple Sclerosis in a Diverse UK Cohort: A Nested Case–Control Study
Source: Ann Clin Transl Neurol. 2025 Sep 24;13(1):71–84. doi: 10.1002/acn3.70175 (PMC12790163; doi:10.1002/acn3.70175)
Supplement: Supplementary file 3 — Table S2: Codes used to define and categorise prodromal symptoms. [file ACN3-13-71-s002.docx]

Supplementary table 2: Codes used to define and categorise prodromal symptoms

| 2a: Main symptom categories and the corresponding subcategories. | | |
| --- | --- | --- |
| Grouped symptoms | Individual symptoms | |
| Autonomic symptoms | Abnormal lacrimation | Hypertension |
|  | Abnormal sweating | Intestinal symptoms |
|  | Anorectal dysfunction | Palpitations |
|  | Anosmia | Rhinorrhoea |
|  | Breathlessness | Sexual dysfunction Female |
|  | Changes in colour | Sexual dysfunction Male |
|  | Cold extremities | Syncope |
|  | Gastric Symptoms | Taste loss |
|  | Heart conduction | Urinary symptoms |
|  | Hiccoughs |  |
| Cognitive symptoms | Abnormal behaviour | Memory problems |
|  | Cognitive impairment |  |
| Neurological symptoms | Balance problem | Muscle weakness |
|  | Bells facial palsy | Sensory symptoms |
|  | Extraocular muscle problem (EOM) | Trigeminal damage |
|  | Impaired vision | Vertigos |
|  | Involuntary movements |  |
| Pain symptoms | Back neck pain | Headache |
|  | Eye pain | Limb pain |
| Psychiatric symptoms | Anxiety | Personality disorders |
|  | Depression | Psychoses |
|  | Eating disorders | Psycogenic disorders |
|  | Gender identity disorder (GID) | Self-harm |
|  | Insomnia | Stress events |
|  | Manic | Tics |

| 2b: Diagnostic readcodes and medcodes for autonomic symptoms. | | | | |
| --- | --- | --- | --- | --- |
| Symptom type | Subtype | ReadCode | MedCode | Term |
| Abnormal lacrimation |  | 1B88.00 | 253092014 | Dry eyes |
|  |  | 1B87.13 | 397988011 | Watery eyes |
|  |  | 1B87.14 | 397990012 | Tearing eye |
|  |  | 1B87.00 | 397991011 | Watery eye |
|  |  | 1B87.11 | 407073019 | C/O - excess tears |
|  |  | 1B87.12 | 407074013 | C/O - watering eyes |
|  |  | ^ESCTCO553286 | 5532861000006113 | Complaining of excess tears |
| Abnormal sweating |  | 1662.12 | 71717017 | Night sweats |
|  |  | 166Z.00 | 252244012 | Sweating symptom NOS |
|  |  | 1662 | 496382011 | Excessive sweating |
|  |  | 1662.11 | 830651000006111 | Hyperhidrosis symptom |
|  |  | 1663 | 1173551000000119 | Sweating asymmetry |
|  |  | EMISNQC/5 | 1727481000006118 | C/O sweating asymmetry |
|  |  | ^ESCTSW335298 | 3352981000006117 | Sweating profusely |
| Anorectal dysfunction |  | 19D2.00 | 252655015 | Tenesmus present |
|  |  | 19DZ.00 | 252656019 | Tenesmus NOS |
|  |  | 3931 | 256943014 | Bowels: occasional accident |
|  |  | 19E2.00 | 397915018 | Soiling - encopresis |
|  |  | 19E2.12 | 411264016 | Soiling symptom |
|  |  | 19E2.11 | 419025011 | Encopresis symptom |
|  |  | 19EF.00 | 501970016 | Urgent desire for stool |
|  |  | 3930 | 1233452016 | Bowels: incontinent |
|  |  | 19F..12 | 1786047017 | Loose stool |
|  |  | 19E3.00 | 781761000006117 | Incontinent of faeces |
|  |  | 19E3.11 | 781771000006112 | Incontinent of faeces symptom |
| Anosmia |  | 2BP3.00 | 255453013 | O/E - anosmia |
|  |  | 1B45.11 | 407070016 | C/O - anosmia |
|  |  | 1B45.12 | 2579255011 | C/O - loss of smell sense |
|  |  | 1B45.00 | 485201000006117 | Loss of sense of smell |
| Breathlessness |  | 1737 | 93172014 | Wheezing |
|  |  | 1732 | 252385016 | Breathless - moderate exertion |
|  |  | 1733 | 252386015 | Breathless - mild exertion |
|  |  | 1734 | 252387012 | Breathless - at rest |
|  |  | 1738 | 344917018 | Difficulty breathing |
|  |  | 173..00 | 397888010 | Breathlessness |
|  |  | 1739 | 397890011 | Shortness of breath |
|  |  | 1737.11 | 407082013 | Wheezing symptom |
|  |  | 173..13 | 140461000006116 | Dyspnoea |
|  |  | 1735 | 525691000006114 | Breathlessness lying flat |
|  |  | 173..11 | 525741000006111 | Breathlessness symptom |
|  |  | 173..12 | 633381000006111 | Dyspnoea - symptom |
|  |  | EMISCDY2 | 982351000006118 | Dyspnoea |
|  |  | 1737000 | 2241471000000111 | Constant wheezing |
| Changes in colour |  | 1673.11 | 370536016 | Blue - symptom |
|  |  | 1674.11 | 397875012 | Pale - symptom |
|  |  | 1672 | 405081019 | Flushes/goes red |
|  |  | 1673 | 405082014 | Goes blue |
|  |  | 1674 | 443390015 | Pale colour |
|  |  | 1672.11 | 762961000006111 | Flushes - symptom |
|  |  | 1672.12 | 804991000006112 | Goes red - symptom |
| Cold extremities |  | 186..00 | 252499018 | C/O cold extremities |
|  |  | 186..11 | 252500010 | C/O cold peripheries |
|  |  | 1860 | 252501014 | C/O cold hands |
|  |  | 1861 | 252502019 | C/O cold feet |
|  |  | 186z.00 | 252503012 | C/O cold extremities NOS |
|  |  | ^ESCTCO274327 | 2743271000006111 | Cold extremities |
|  |  | ^ESCTCO454574 | 4545741000006116 | Complaining of cold extremities |
|  |  | ^ESCTCO454576 | 4545761000006117 | Complaining of cold hands |
|  |  | ^ESCTCO454578 | 4545781000006110 | Complaining of cold feet |
|  |  | ^ESCTCO552640 | 5526401000006110 | Cold hands |
|  |  | ^ESCTCO552641 | 5526411000006113 | Cold feet |
| Gastric Symptoms |  | 199..13 | 62358011 | Rumination |
|  |  | 1982 | 252600018 | Nausea present |
|  |  | 1984 | 252602014 | Upset stomach |
|  |  | 1984.11 | 252603016 | Upset tummy |
|  |  | 198Z.00 | 252604010 | Nausea NOS |
|  |  | 199Z.00 | 252622013 | Vomiting NOS |
|  |  | 199..14 | 372248013 | Vomiting symptom |
|  |  | 199..11 | 407086011 | C/O - vomiting |
|  |  | 1983 | 2478737017 | Morning nausea |
|  |  | 198..00 | 2643930014 | Nausea |
|  |  | 1992 | 2643931013 | Vomiting |
|  |  | 199..00 | 63631000006119 | Finding of vomiting |
|  |  | 1992.11 | 100841000006111 | Throwing up |
|  |  | 1992.12 | 512041000006111 | Bilious attack |
|  |  | 198..11 | 531181000006118 | C/O - nausea |
|  |  | 198..12 | 681151000006114 | Nausea symptoms |
| Heart conduction |  | G562.00 | 9313016 | Left bundle branch hemiblock |
|  |  | G573100 | 9988012 | Atrial flutter |
|  |  | G57y700 | 19216010 | Sinus tachycardia |
|  |  | G572000 | 20729015 | Essential paroxysmal tachycardia |
|  |  | G567000 | 28446019 | Accelerated atrioventricular conduction |
|  |  | G567.00 | 30181017 | Anomalous atrioventricular excitation |
|  |  | G571.11 | 42864016 | Ventricular tachycardia |
|  |  | G560.00 | 46670010 | Complete atrioventricular block |
|  |  | G560.11 | 46671014 | Third degree atrioventricular block |
|  |  | G561200 | 47191011 | Mobitz type II atrioventricular block |
|  |  | G576011 | 49718019 | Extrasystoles |
|  |  | G576.00 | 55752016 | Ectopic beats |
|  |  | G562000 | 62986012 | Left anterior fascicular block |
|  |  | G57y000 | 74409013 | Persistent sinus bradycardia |
|  |  | G57y100 | 81702012 | Severe sinus bradycardia |
|  |  | G573000 | 82343012 | Atrial fibrillation |
|  |  | G564.00 | 98239015 | Right bundle branch block |
|  |  | G562100 | 103118017 | Left posterior fascicular block |
|  |  | G562.11 | 105498014 | Left bundle branch block |
|  |  | G566000 | 109275012 | Sinoatrial block |
|  |  | G571.00 | 110733013 | Paroxysmal ventricular tachycardia |
|  |  | G570.00 | 111643014 | Paroxysmal supraventricular tachycardia |
|  |  | G57y600 | 119266015 | Nodal rhythm disorder |
|  |  | G577.00 | 119267012 | Sinus arrhythmia |
|  |  | G574000 | 119481012 | Ventricular fibrillation |
|  |  | G565500 | 122915018 | Bifascicular block |
|  |  | G565400 | 142607010 | Trifascicular block |
|  |  | G574100 | 178509016 | Ventricular flutter |
|  |  | 2421 | 254021018 | O/E - pulse rate very slow |
|  |  | 2422 | 254022013 | O/E - pulse rate - bradycardia |
|  |  | 2423 | 254023015 | O/E - pulse borderline slow |
|  |  | 2425 | 254025010 | O/E - pulse borderline fast |
|  |  | 2426 | 254026011 | O/E - pulse rate tachycardia |
|  |  | 2427 | 254027019 | O/E - pulse rate very fast |
|  |  | 2432 | 254039010 | On examination - pulse irregularly irregular |
|  |  | 2433 | 254040012 | O/E -pulse regularly irregular |
|  |  | G561111 | 256541019 | Prolonged PR interval |
|  |  | G561.00 | 300067019 | Partial atrioventricular block |
|  |  | G561000 | 300068012 | Atrioventricular block unspecified |
|  |  | G561400 | 300073018 | Second degree atrioventricular block |
|  |  | G561z00 | 300074012 | Atrioventricular block |
|  |  | G562z00 | 300078010 | Left bundle branch hemiblock NOS |
|  |  | G563.00 | 300079019 | Left main stem bundle branch block |
|  |  | G565.00 | 300080016 | Bundle branch block |
|  |  | G565000 | 300081017 | Bundle branch block unspecified |
|  |  | G565300 | 300084013 | Bilateral bundle branch block |
|  |  | G565z00 | 300086010 | Other bundle branch block NOS |
|  |  | G566.00 | 300087018 | Other heart block |
|  |  | G566100 | 300088011 | Interventricular block NOS |
|  |  | G566z00 | 300090012 | Other heart block NOS |
|  |  | G567300 | 300094015 | Ventricular pre-excitation |
|  |  | G567z00 | 300095019 | Anomalous atrioventricular excitation NOS |
|  |  | G56yz00 | 300099013 | Other conduction disorders NOS |
|  |  | G56z.00 | 300100017 | Conduction disorders unspecified |
|  |  | G56zz00 | 300103015 | Conduction disorder of the heart |
|  |  | G570000 | 300106011 | Paroxysmal atrial tachycardia |
|  |  | G570100 | 300110014 | Paroxysmal atrioventricular tachycardia |
|  |  | G570200 | 300111013 | Paroxysmal junctional tachycardia |
|  |  | G570300 | 300114017 | Paroxysmal nodal tachycardia |
|  |  | G570z00 | 300119010 | Paroxysmal supraventricular tachycardia NOS |
|  |  | G572.00 | 300126010 | Paroxysmal tachycardia |
|  |  | G572z00 | 300129015 | Paroxysmal tachycardia NOS |
|  |  | G573.00 | 300130013 | Atrial fibrillation and flutter |
|  |  | G573z00 | 300132017 | Atrial fibrillation and flutter NOS |
|  |  | G574.00 | 300133010 | Ventricular fibrillation and flutter |
|  |  | G574z00 | 300134016 | Ventricular fibrillation and flutter NOS |
|  |  | G576z00 | 300155019 | Premature beats |
|  |  | G57y400 | 300163018 | Sinus node dysfunction |
|  |  | G57y800 | 300166014 | Bigeminal pulse |
|  |  | G57y900 | 300167017 | Supraventricular tachycardia |
|  |  | G57yA00 | 300168010 | Re-entry ventricular arrhythmia |
|  |  | G57yz00 | 300169019 | Other cardiac dysrhythmia NOS |
|  |  | G57z.00 | 300170018 | Cardiac dysrhythmia NOS |
|  |  | G573300 | 350465014 | Non-rheumatic atrial fibrillation |
|  |  | G56..12 | 350471015 | Heart block |
|  |  | G57y.12 | 371119011 | Pulse missed beats |
|  |  | G57y.13 | 371120017 | Skipped beat |
|  |  | G57y.14 | 371151012 | Irregular heart beat |
|  |  | G576400 | 374316010 | Junctional premature depolarization |
|  |  | G576500 | 374331018 | Ventricular premature depolarisation |
|  |  | G576000 | 395770011 | Ectopic beats unspecified |
|  |  | G57y.00 | 395771010 | Other cardiac dysrhythmias |
|  |  | G561100 | 405111010 | First degree atrioventricular block |
|  |  | 243..11 | 411930014 | O/E - irregular pulse |
|  |  | G573200 | 421235014 | Paroxysmal atrial fibrillation |
|  |  | G576300 | 426568019 | Atrial premature depolarisation |
|  |  | G567200 | 477949015 | Pre-excitation atrioventricular conduction |
|  |  | G567100 | 477950015 | Accessory atrioventricular conduction |
|  |  | G57..00 | 1230146013 | Cardiac dysrhythmia |
|  |  | G57..11 | 1230147016 | Cardiac arrhythmia |
|  |  | G561311 | 1231273012 | Mobitz type 1 second degree atrioventricular block |
|  |  | G56y400 | 1231921018 | Right fascicular block |
|  |  | G56y500 | 1235983011 | Long Q-T syndrome |
|  |  | G576200 | 2619422017 | Ventricular ectopic beats |
|  |  | G573600 | 2675306013 | Paroxysmal atrial flutter |
|  |  | G578.00 | 2920703018 | Atrial standstill |
|  |  | G576100 | 117041000006118 | Supraventricular ectopic beats |
|  |  | G565200 | 162981000006110 | Right bundle branch block with left anterior fascicular block |
|  |  | G565100 | 162991000006113 | Right bundle branch block with left posterior fascicular block |
|  |  | G566200 | 163011000006112 | Right fascicular block |
|  |  | G57y.11 | 194271000006110 | Pulsus alternans |
|  |  | G576.11 | 214431000006114 | Premature beats |
|  |  | G56..11 | 218471000000114 | Conduction disorders of heart |
|  |  | G56..00 | 223971000000115 | Disorder of heart conduction |
|  |  | 2426.11 | 274941000006115 | O/E - tachycardia |
|  |  | 2422.11 | 289521000006112 | O/E - bradycardia |
|  |  | G56y100 | 498091000006111 | Atrioventricular dissociation |
|  |  | G574011 | 537191000006113 | Cardiac arrest - ventricular fibrillation |
|  |  | G573500 | 636701000000115 | Persistent atrial fibrillation |
|  |  | G573400 | 636721000000112 | Permanent atrial fibrillation |
|  |  | G561300 | 699951000006113 | Mobitz type I Wenckebach atrioventricular block |
|  |  | G560.99 | 884341000006118 | Complete atrioventric. block |
|  |  | G566.99 | 884351000006116 | Heart block NOS |
|  |  | G570.99 | 884361000006119 | Parox. supravent. tachycardia |
|  |  | G571.99 | 884371000006114 | Paroxysmal ventric. tachyc. |
|  |  | G576.99 | 884381000006112 | Ectopic beats/extrasystoles |
|  |  | G57y.99 | 884391000006110 | Cardiac dysrhythmias NOS |
|  |  | G57z.99 | 884401000006112 | Cardiac dysrhythmias NOS |
|  |  | G57..99 | 991651000006115 | Cardiac dysrhythmias NOS |
|  |  | 2435 | 1765601000006112 | O/E - irregular pulse |
| Hiccoughs |  | 174..00 | 109542015 | Hiccoughs |
|  |  | 1742 | 252417017 | Hiccough present |
|  |  | 174Z.00 | 252418010 | Hiccough |
|  |  | 174..11 | 822841000006111 | Hiccough symptom |
| Hypertension |  | G201.00 | 3135013 | Benign essential hypertension |
|  |  | G2...00 | 64168014 | Hypertensive disease |
|  |  | G20..11 | 64172013 | Elevated blood pressure |
|  |  | G203.00 | 80224019 | Diastolic hypertension |
|  |  | G202.00 | 93494011 | Systolic hypertension |
|  |  | G20..00 | 99042012 | Essential hypertension |
|  |  | G20..12 | 99047018 | Primary hypertension |
|  |  | G21..00 | 107545013 | Hypertensive heart disease |
|  |  | G200.00 | 131046010 | Malignant essential hypertension |
|  |  | G20z.00 | 395751018 | Essential hypertension NOS |
|  |  | G2...11 | 523801000006119 | BP - hypertensive disease |
|  |  | G20z.11 | 790121000006116 | Hypertension |
|  |  | G200.99 | 884121000006111 | Malignant hypertension |
|  |  | G26..00 | 1846961000006115 | Severe hypertension (NICE - National Institute for Health and Clinical Excellence 2011) |
|  |  | G26..11 | 2193021000000110 | Severe hypertension |
|  |  | ^ESCTHT311748 | 3117481000006113 | HT - Hypertension |
| Intestinal symptoms | Abdominal bloating wind | 19A..00 | 252623015 | Abdominal distension symptom |
|  |  | 19AZ.00 | 252628012 | Abd. distension symptom NOS |
|  |  | 19B3.00 | 252640012 | Excessive belching |
|  |  | 19BZ.00 | 252642016 | Wind NOS |
|  |  | 19B..12 | 370924017 | Bloating symptom |
|  |  | 19B..00 | 397913013 | Wind - flatus |
|  |  | 19B..11 | 411263010 | Belching symptom |
|  |  | 19B..14 | 452022012 | Flatulence symptom |
|  |  | 19B5.00 | 504392014 | Excessive flatus |
|  |  | 19A2.00 | 2475423012 | Abdomen feels bloated |
|  |  | 19A3.00 | 2475503014 | Abdomen feels distended |
|  |  | 19B..15 | 2620502012 | Wind symptom |
|  |  | 19A4.00 | 436421000006112 | Abdomen feels swollen |
|  |  | 19B2.00 | 650701000006112 | Excessive flatulence |
|  | Constipation | 19C..00 | 25076018 | Constipation |
|  |  | 19CZ.00 | 303166010 | Constipation NOS |
|  |  | 19C2.00 | 2162207016 | Constipated |
|  |  | 19C..11 | 590111000006111 | Constipation symptom |
|  |  | 19C..12 | 598301000006114 | Costive symptom |
|  | Diarrhoea | 19F2.00 | 103578017 | Diarrhoea |
|  |  | 19E8.11 | 372415011 | Floating faeces symptom |
|  |  | 19E7.00 | 397920018 | Offensive faeces |
|  |  | 19E8.00 | 397924010 | Floating faeces |
|  |  | 19F..00 | 397927015 | Diarrhoea symptoms |
|  |  | 19FZ.00 | 397928013 | Diarrhoea symptom |
|  |  | 19E7.11 | 407090013 | Offensive faeces symptom |
|  |  | 19EA.00 | 507901013 | Change in bowel habit |
|  |  | 19EA.11 | 507902018 | Altered bowel habit |
|  |  | 19F3.00 | 131671000006119 | Spurious diarrhoea - overflow |
|  |  | 19F..11 | 619741000006114 | Diarrhoea |
|  |  | 19FZ.11 | 619771000006118 | Diarrhoea and vomiting, symptom |
| Palpitations |  | 1812 | 133268018 | Palpitations |
|  |  | 181Z.00 | 252443016 | Palpitations NOS |
|  |  | 181..11 | 217661000000115 | Awareness of heartbeat |
|  |  | 181..12 | 217671000000110 | Fluttering of heart |
|  |  | 181..00 | 249291000006117 | Palpitations |
|  |  | 1813 | 291811000006119 | Bumping heart |
|  |  | 1814 | 291831000006113 | Fluttering heart |
| Rhinorrhoea |  | 1C85.00 | 253218018 | Swollen nose |
|  |  | 1C8Z.00 | 253220015 | Nasal symptom |
|  |  | 1C83.11 | 397999013 | Nose running |
|  |  | 1C83.12 | 398000019 | Nose dripping |
|  |  | 1C83.13 | 2161421018 | Rhinorrhoea |
|  |  | 1C86.00 | 517051000006111 | Nasal obstruction |
|  |  | 1C84.00 | 531231000006113 | C/O - postnasal drip |
|  |  | ^ESCTPO373245 | 3732451000006116 | Postnasal drip |
|  |  | ^ESCTPN373248 | 3732481000006112 | PND - Postnasal drip |
|  |  | ^ESCTCO454824 | 4548241000006116 | Complaining of postnasal drip |
| Sexual dysfunction Female |  | 1AD..00 | 53315015 | Vaginal dryness |
|  |  | 15F..00 | 131111016 | Vaginismus |
|  |  | 15C..00 | 252161016 | Vaginal irritation |
|  |  | 15H..00 | 252163018 | Vulval irritation |
|  |  | 1AE..00 | 252881018 | Vaginal discomfort |
|  |  | 15D..00 | 504772016 | Pain on sexual intercourse |
|  |  | 15E..00 | 64171000006110 | Vulval irritation |
|  |  | K580.00 | 633191000006112 | Dyspareunia due to non-psychogenic cause in the female |
|  |  | K580.99 | 887471000006112 | Dyspareunia - non psychogenic |
|  |  | EMISCDY1 | 962071000006111 | Dyspareunia |
|  |  | EMISNQOE46 | 1861111000006119 | O/E - vulval rash |
|  |  | 15J..00 | 2279851000000117 | Vulval rash |
|  |  | ^ESCTVU364694 | 3646941000006115 | Vulvovaginal discomfort |
|  |  | ^ESCTDY366015 | 3660151000006110 | Dyspareunia |
| Sexual dysfunction Male |  | 1ABE.00 | 9451017 | Abnormal angle of erection |
|  |  | 1ABF.00 | 371276015 | Penetration impossible |
|  |  | 1ABB.00 | 371290017 | Cannot get an erection |
|  |  | 1ABC.00 | 371298012 | Cannot sustain an erection |
|  |  | E227300 | 1777409015 | Impotence |
|  |  | E227311 | 1786154013 | Erectile dysfunction |
|  |  | Eu52212 | 396141000006114 | [X]Male erectile disorder |
|  |  | 1D1B.00 | 1734321000000113 | C/O erectile dysfunction |
|  |  | ^ESCTPO357750 | 3577501000006115 | Poor erection |
|  |  | ^ESCT1395247 | 13952471000006117 | Erectile dysfunction |
| Syncope |  | 1B68.00 | 253056010 | Felt faint |
|  |  | 1B6..13 | 370568012 | Vasovagal symptom |
|  |  | 1B6..12 | 407072012 | Syncope symptom |
|  |  | 1B62.00 | 112741000006118 | Vasovagal syncope |
|  |  | 1B6..11 | 663641000006119 | Faint symptom |
|  |  | ^ESCTFA552650 | 5526501000006111 | Fainting |
|  |  | ^ESCTFA552651 | 5526511000006114 | Faint |
| Taste loss |  | 1924 | 61644018 | Loss of taste |
|  |  | 1924.11 | 407083015 | C/O - loss of taste sense |
| Urinary symptoms | Non infective urinary symptoms-incontinence | 1A25.11 | 124716012 | Urgency of micturition |
|  |  | 1A36.00 | 252803018 | Terminal dribbling of urine |
|  |  | 3940 | 256950013 | Bladder: incontinent |
|  |  | 3941 | 256951012 | Bladder: occasional accident |
|  |  | 1A37.00 | 498306016 | Dribbling of urine |
|  |  | 1A35.11 | 502907019 | Precipitancy of micturition |
|  |  | 1A26.00 | 507635014 | Urge incontinence of urine |
|  |  | 1A23.00 | 1494848017 | Incontinence of urine |
|  |  | 1A25.00 | 74221000006110 | Urgent desire to urinate |
|  |  | 1A24.00 | 121721000006114 | Stress incontinence |
|  |  | 1A24.11 | 121751000006117 | Stress incontinence - symptom |
|  |  | 1A35.00 | 213441000006118 | Precipitancy |
|  |  | 1A27.00 | 529601000000112 | Urge to pass urine again shortly after finishing voiding |
|  |  | 1A27.11 | 529651000000113 | Pis en deux |
|  | Non infective urinary symptoms-retention | 1A33.00 | 252800015 | Micturition stream poor |
|  |  | 1A32.00 | 397940013 | Cannot pass urine - retention |
|  |  | 1A34.11 | 1231994019 | Hesitancy of micturition |
|  |  | 1A34.00 | 1231995018 | Hesitancy |
|  |  | 1A32.11 | 169481000006113 | Retention - symptom |
|  | Urinary symptoms-clearly neurological | F246111 | 497251000006113 | Atonic bladder |
|  |  | F246113 | 675651000006110 | Neuropathic bladder |
|  |  | F246112 | 676521000006111 | Neurogenic bladder |
|  | Urinary symptoms-non specific | 1A56.00 | 28519014 | Strangury |
|  |  | 1A55.00 | 82701015 | Dysuria |
|  |  | 1AZ3.00 | 255420015 | Difficulty with micturition |

| 2c: Diagnostic readcodes and medcodes for cognitive symptoms. | | | | |
| --- | --- | --- | --- | --- |
| Symptom type | Subtype | ReadCode | MedCode | Term |
| Abnormal behaviour |  | 3AB2.00 | 257057017 | Severely abnormal behaviour |
|  |  | 3AB3.00 | 459735014 | Change in behaviour |
|  |  | 3AB1.00 | 702141000006114 | Mildly abnormal behaviour |
|  |  | ^ESCTAB291252 | 2912521000006115 | Abnormal behaviour |
|  |  | ^ESCTUN707894 | 7078941000006119 | Unusual change in behaviour |
| Cognitive impairment |  | 2B52.00 | 255257012 | O/E - agnosia |
|  |  | 2B53.00 | 255258019 | O/E - apraxia |
|  |  | 28E3.00 | 1491795010 | Cognitive impairment |
|  |  | 28E..00 | 1491798012 | Cognitive decline |
|  |  | Eu05700 | 215831000000117 | Mild cognitive disorder |
|  |  | Ryu5100 | 404241000006118 | Impaired cognition |
|  |  | EMISNQIM12 | 1807641000006115 | Impaired cognition |
|  |  | 28E0.00 | 2288201000000116 | Mild cognitive impairment |
|  |  | 28E1.00 | 2288241000000118 | Moderate cognitive impairment |
|  |  | 28E2.00 | 2288281000000114 | Severe cognitive impairment |
| Memory problems |  | 1B1A000 | 252928010 | Temporary loss of memory |
|  |  | 3A10.00 | 257013016 | Memory: own age not known |
|  |  | 3A20.00 | 257017015 | Memory: present time not known |
|  |  | 3A40.00 | 257025018 | Memory: present year not known |
|  |  | 3A50.00 | 257029012 | Memory: own DOB not known |
|  |  | 3A60.00 | 257033017 | Memory: present month not knwn |
|  |  | 3A70.00 | 257037016 | Memory: important event not knwn |
|  |  | 3A80.00 | 257041017 | Memory: important person not known |
|  |  | E2A1000 | 295525018 | Mild memory disturbance |
|  |  | E2A1100 | 295526017 | Organic memory impairment |
|  |  | 1B1A100 | 369704019 | Short-term memory loss |
|  |  | 1B1A.00 | 495013011 | Memory loss - amnesia |
|  |  | 28G..00 | 497290018 | Forgetful |
|  |  | R00z011 | 1222481019 | Memory deficit |
|  |  | 1S23.00 | 1480927011 | Memory impairment |
|  |  | 1B1Y.00 | 2474637019 | Poor visual sequential memory |
|  |  | 1B1a.00 | 2474638012 | Poor auditory sequential memory |
|  |  | 1B1A.11 | 479241000006114 | Amnesia symptom |
|  |  | 3A30.00 | 710751000006111 | Memory: present place not known |
|  |  | 1B1A.13 | 711371000006111 | Memory disturbance |
|  |  | 1B1A.12 | 711391000006112 | Amnesia |
|  |  | 3AA1.00 | 711421000006116 | Memory: address recall unsuccessful |
|  |  | 3A91.00 | 711451000006113 | Memory: count down unsuccessful |
|  |  | ^ESCTPO646218 | 6462181000006110 | Poor memory |

| 2d: Diagnostic readcodes and medcodes for neurological symptoms. | | | | |
| --- | --- | --- | --- | --- |
| Symptom type | Subtype | ReadCode | MedCode | Term |
| Balance problem |  | 1B53.00 | 253031016 | Dizziness present |
|  |  | 1B54.00 | 253032011 | Giddiness present |
|  |  | 1B5Z.00 | 253033018 | Incoordination symptom NOS |
|  |  | 2993.11 | 254950010 | O/E - ataxic gait |
|  |  | 2993 | 254951014 | O/E - gait ataxic |
|  |  | 29L8.00 | 255069012 | O/E - generally unsteady |
|  |  | 29L8.11 | 255070013 | O/E - generally off balance |
|  |  | 1B52.11 | 372880015 | Feels off balance |
|  |  | 1B5..00 | 397975013 | Incoordination symptom |
|  |  | 1B52.00 | 397976014 | Unsteadiness present |
|  |  | 29LB.00 | 420581019 | Unable to balance |
|  |  | 1B55.00 | 2159245017 | Dizziness on standing up |
|  |  | 1B5..13 | 72741000006118 | General unsteadiness |
|  |  | 29LD.00 | 528701000000112 | Disorder of gait and/or balance present |
|  |  | 1B5..11 | 627241000006116 | Dizziness |
|  |  | 1B5..12 | 802161000006111 | Giddiness |
|  |  | 29LF.00 | 1746251000000117 | Worsening balance |
| Bells facial palsy |  | 2BR6.00 | 255474014 | O/E -cranial nerve 7-palsy-LMN |
|  |  | 2BR7.00 | 255475010 | O/E -cranial 7 -paralysis -LMN |
|  |  | 2BR8.00 | 255478012 | O/E -cranial nerve -palsy -UMN |
|  |  | 2BR9.00 | 255479016 | O/E - cranial 7 -paralysis-UMN |
|  |  | F310.00 | 504611000006114 | Bell's palsy |
| EOM |  | F4K5.11 | 2016015 | Nystagmus |
|  |  | F4K5500 | 16663019 | Dissociated nystagmus |
|  |  | F4J5500 | 32650015 | External ophthalmoplegia |
|  |  | 1B72.11 | 41870012 | Diplopia |
|  |  | 1B72.12 | 41871011 | Double vision |
|  |  | F4Jy800 | 67776011 | Skew deviation |
|  |  | F4Jy700 | 82990018 | Internuclear ophthalmoplegia |
|  |  | F4J5600 | 129611013 | Total ophthalmoplegia |
|  |  | 2BD..00 | 255363018 | O/E - strabismus - squint |
|  |  | 2BD..12 | 255364012 | O/E - strabismus |
|  |  | 2BD..11 | 255365013 | O/E - squint |
|  |  | 2BD1.00 | 255366014 | O/E - convergent squint |
|  |  | 2BD2.00 | 255367017 | O/E - divergent squint |
|  |  | 2BDZ.00 | 255368010 | O/E - strabismus NOS |
|  |  | 2BJ2.00 | 255407018 | O/E - eye does not move up |
|  |  | 2BJ3.00 | 255408011 | O/E - eye does not move down |
|  |  | 2BJ4.00 | 255409015 | O/E - eye does not move left |
|  |  | 2BJ5.00 | 255410013 | O/E - eye does not move right |
|  |  | 2BN2.00 | 255441014 | O/E - visual nystagmus |
|  |  | 2BN4.00 | 255443012 | O/E - nystagmus - CNS -regular |
|  |  | 2BNZ.00 | 255444018 | O/E - nystagmus NOS |
|  |  | 2BQ3.00 | 255459012 | O/E - cranial nerve 3 - palsy |
|  |  | 2BQ4.00 | 255460019 | O/E -cranial nerve 3-paralysis |
|  |  | 2BQ6.00 | 255462010 | O/E - cranial nerve 4 - palsy |
|  |  | 2BQ9.00 | 255465012 | O/E - cranial nerve 6 - palsy |
|  |  | 2BQA.00 | 255466013 | O/E -cranial nerve 6-paralysis |
|  |  | F4J5100 | 298822011 | Partial oculomotor nerve palsy |
|  |  | F4J5111 | 298824012 | Third nerve palsy - partial |
|  |  | F4J5211 | 298826014 | Third nerve palsy - total |
|  |  | F4J5200 | 298827017 | Total oculomotor nerve palsy |
|  |  | F4J5z00 | 298828010 | Paralytic squint NOS |
|  |  | F4K5000 | 298905010 | Unspecified nystagmus |
|  |  | F4K5600 | 298907019 | Other forms of nystagmus |
|  |  | F4K5z00 | 298912018 | Nystagmus or other irregular eye movement NOS |
|  |  | FyuK200 | 299503015 | [X]Other specified strabismus |
|  |  | 1B81.00 | 397982012 | Has a squint |
|  |  | F4Jy900 | 399487018 | Other eye movement dissociation or deviation |
|  |  | F4K5.00 | 399493014 | Nystagmus and other irregular eye movements |
|  |  | 2BJ7.00 | 458691015 | O/E - poor visual fixation |
|  |  | F4J5400 | 1786558018 | Abducens (sixth) nerve palsy |
|  |  | F4J5300 | 88511000006119 | Trochlear (fourth) nerve palsy |
|  |  | 1B81.11 | 132091000006117 | Squint - symptom |
|  |  | 2BN3.00 | 271371000006112 | Irregular nystagmus |
|  |  | F4K5400 | 296521000006116 | Nystagmus with vestibular disorder |
|  |  | F482.00 | 622011000006112 | Diplopia (double vision) |
|  |  | 1B72.00 | 622021000006116 | Diplopia/double vision |
|  |  | F482.99 | 883331000006114 | Diplopia - double vision |
|  |  | F4J..99 | 883571000006117 | Strabismus/squint |
| Impaired vision |  | F49..11 | 14191011 | Impaired vision |
|  |  | F481100 | 25807010 | Sudden visual loss |
|  |  | F484H00 | 56892016 | Homonymous hemianopia |
|  |  | F484100 | 65329015 | Central scotoma |
|  |  | F484M00 | 102912011 | Bitemporal hemianopia |
|  |  | F484300 | 107082011 | Paracentral scotoma |
|  |  | F484L00 | 144724014 | Binasal hemianopia |
|  |  | 1B73.00 | 253064016 | Flashing lights seen |
|  |  | 1B76.00 | 253067011 | Temporary visual disturbance |
|  |  | 1B7Z.00 | 253069014 | Visual symptom NOS |
|  |  | 2B6..00 | 255262013 | O/E - visual acuity R-eye |
|  |  | 2B61.00 | 255263015 | O/E - visual acuity R-eye =6/5 |
|  |  | 2B62.00 | 255264014 | O/E - visual acuity R-eye =6/6 |
|  |  | 2B63.00 | 255265010 | O/E - visual acuity R-eye =6/9 |
|  |  | 2B64.00 | 255266011 | O/E - visual acuity R-eye=6/12 |
|  |  | 2B65.00 | 255267019 | O/E - visual acuity R-eye=6/18 |
|  |  | 2B66.00 | 255268012 | O/E - visual acuity R-eye=6/24 |
|  |  | 2B67.00 | 255269016 | O/E - visual acuity R-eye=6/36 |
|  |  | 2B68.00 | 255270015 | O/E - visual acuity R-eye=6/60 |
|  |  | 2B69.00 | 255271016 | O/E -R-eye counts fingers only |
|  |  | 2B6A.11 | 255275013 | O/E - blind R-eye |
|  |  | 2B6B.00 | 255276014 | O/E - R-eye completely blind |
|  |  | 2B7..00 | 255279019 | O/E - visual acuity L-eye |
|  |  | 2B71.00 | 255280016 | O/E - visual acuity L-eye =6/5 |
|  |  | 2B72.00 | 255281017 | O/E - visual acuity L-eye =6/6 |
|  |  | 2B73.00 | 255282012 | O/E - visual acuity L-eye =6/9 |
|  |  | 2B74.00 | 255283019 | O/E - visual acuity L-eye=6/12 |
|  |  | 2B75.00 | 255284013 | O/E - visual acuity L-eye=6/18 |
|  |  | 2B76.00 | 255285014 | O/E - visual acuity L-eye=6/24 |
|  |  | 2B77.00 | 255286010 | O/E - visual acuity L-eye=6/36 |
|  |  | 2B78.00 | 255287018 | O/E - visual acuity L-eye=6/60 |
|  |  | 2B79.00 | 255288011 | O/E -L-eye counts fingers only |
|  |  | 2B7A.11 | 255292016 | O/E - blind L-eye |
|  |  | 2B7B.00 | 255293014 | O/E - L-eye completely blind |
|  |  | 2B7Z.00 | 255295019 | O/E - visual acuity L-eye NOS |
|  |  | 2B9Z.00 | 255315016 | O/E - colour blindness NOS |
|  |  | 2BC2.00 | 255354015 | O/E - hemianopia |
|  |  | 2BC3.00 | 255355019 | O/E - homonymous hemianopia |
|  |  | 2BC4.00 | 255356018 | O/E - bitemporal hemianopia |
|  |  | 2BC5.00 | 255357010 | O/E - binasal hemianopia |
|  |  | 2BC6.00 | 255360015 | O/E - quadrantic hemianopia |
|  |  | 2BC7.00 | 255361016 | O/E - central scotoma |
|  |  | 2BCZ.00 | 255362011 | O/E - visual fields NOS |
|  |  | F481000 | 298166010 | Unspecified subjective visual disturbance |
|  |  | F484000 | 298194019 | Unspecified visual field defect |
|  |  | F484400 | 298198016 | Central area scotoma NOS |
|  |  | F484500 | 298199012 | Blind spot scotoma |
|  |  | F484F00 | 298207013 | Visual field scotoma |
|  |  | F484J00 | 298213016 | Homonymous quadrant anopia |
|  |  | F484K00 | 298214010 | Other homonymous bilateral field defect |
|  |  | F484N00 | 298215011 | Other heteronymous bilateral field defect |
|  |  | F484z00 | 298216012 | Visual field defects NOS |
|  |  | F485.00 | 298220011 | Colour vision deficiency |
|  |  | F485z00 | 298226017 | Colour blindness NOS |
|  |  | F48yz00 | 298241017 | Other specified visual disturbance NOS |
|  |  | F48z.00 | 298242012 | Visual disturbance NOS |
|  |  | F490000 | 298252011 | Unspecified blindness both eyes |
|  |  | F490z00 | 298262016 | Blindness both eyes NOS |
|  |  | F491000 | 298264015 | One eye blind, one eye low vision |
|  |  | F491z00 | 298276015 | One eye blind, one eye low vision NOS |
|  |  | F492.00 | 298277012 | Low vision, both eyes |
|  |  | F492000 | 298278019 | Low vision, both eyes unspecified |
|  |  | F492z00 | 298284016 | Low vision, both eyes NOS |
|  |  | F493.00 | 298285015 | Blind or low vision - both eyes |
|  |  | F495.00 | 298287011 | Profound impairment, one eye |
|  |  | F495000 | 298288018 | Blindness of one eye |
|  |  | F495100 | 298289014 | Lesser eye: total visual impairment, Better eye: unspecified |
|  |  | F495z00 | 298299016 | Profound impairment one eye NOS |
|  |  | F496.00 | 298300012 | Low vision, one eye |
|  |  | F496000 | 298301011 | Low vision, one eye, unspecified |
|  |  | F496z00 | 298308017 | Low vision, one eye NOS |
|  |  | 1B77.00 | 338995019 | Deteriorating vision |
|  |  | F48y000 | 399453011 | Blurred vision |
|  |  | 2B6A.00 | 402527016 | O/E-R-eye perceives light only |
|  |  | 2B7A.00 | 402528014 | O/E-L-eye perceives light only |
|  |  | 2B9..11 | 411867017 | O/E - colour blindness |
|  |  | 2B6C.00 | 451387013 | O/E - R-eye sees hand movements |
|  |  | 2B7C.00 | 451388015 | O/E - L-eye sees hand movements |
|  |  | F490900 | 456798010 | Acquired blindness, both eyes |
|  |  | F495A00 | 456799019 | Acquired blindness, one eye |
|  |  | F484200 | 485392014 | Centrocaecal scotoma |
|  |  | F481500 | 501548010 | Simple eye strain |
|  |  | F481511 | 501549019 | Tired eyes |
|  |  | F485400 | 1216889011 | Acquired colour blindness |
|  |  | 2B6D.00 | 1484837010 | O/E - visual acuity R-eye=6/4 |
|  |  | 2B6E.00 | 1484838017 | O/E - visual acuity R-eye=3/60 |
|  |  | 2B7D.00 | 1484839013 | O/E - visual acuity L-eye =6/4 |
|  |  | 2B7E.00 | 1484840010 | O/E - visual acuity L-eye=3/60 |
|  |  | 1B78.00 | 1484876011 | Scotopic sensitivity |
|  |  | 1B79.00 | 1488913019 | Visual perceptual weakness |
|  |  | 1B7A.00 | 1780217018 | Patient concerned about eyesight |
|  |  | 2B7F.00 | 2163939011 | O/E - pinhole visual acuity L-eye=6/9 |
|  |  | 2B7G.00 | 2163940013 | O/E - pinhole visual acuity L-eye=6/12 |
|  |  | 2B7H.00 | 2163941012 | O/E - pinhole visual acuity L-eye=6/18 |
|  |  | 2B7J.00 | 2163942017 | O/E - pinhole visual acuity L-eye =6/24 |
|  |  | 2B7K.00 | 2163943010 | O/E - pinhole visual acuity L-eye=6/36 |
|  |  | 2B7L.00 | 2163944016 | O/E - pinhole visual acuity L-eye=6/60 |
|  |  | 2B7M.00 | 2163945015 | O/E - pinhole visual acuity L-eye =6/5 |
|  |  | 2B7N.00 | 2163946019 | O/E - pinhole visual acuity L-eye =6/6 |
|  |  | 2B6F.00 | 2163947011 | O/E - pinhole visual acuity R-eye=6/9 |
|  |  | 2B6G.00 | 2163948018 | O/E - pinhole visual acuity R-eye=6/12 |
|  |  | 2B6H.00 | 2163949014 | O/E - pinhole visual acuity R-eye=6/18 |
|  |  | 2B6J.00 | 2163950014 | O/E - pinhole visual acuity R-eye=6/24 |
|  |  | 2B6K.00 | 2163951013 | O/E - pinhole visual acuity R-eye=6/36 |
|  |  | 2B6L.00 | 2163952018 | O/E - pinhole visual acuity R-eye=6/60 |
|  |  | 2B6M.00 | 2163953011 | O/E - pinhole visual acuity R-eye=6/6 |
|  |  | 2B6N.00 | 2163954017 | O/E - pinhole visual acuity R-eye=6/5 |
|  |  | 2B7P.00 | 2163955016 | O/E - pinhole L-eye sees hand movements |
|  |  | 2B7Q.00 | 2163956015 | O/E - pinhole L-eye counts fingers only |
|  |  | 2B7R.00 | 2163957012 | O/E - pinhole L-eye perceives light only |
|  |  | 2B7S.00 | 2163958019 | O/E - pinhole L-eye completely blind |
|  |  | 2B6P.00 | 2163959010 | O/E - pinhole R-eye sees hand movements |
|  |  | 2B6Q.00 | 2163960017 | O/E - pinhole R-eye counts fingers only |
|  |  | 2B6R.00 | 2163961018 | O/E - pinhole R-eye perceives light only |
|  |  | 2B6S.00 | 2163962013 | O/E - pinhole R-eye completely blind |
|  |  | 2B6T.00 | 2163963015 | O/E - R-eye visual acuity (corrected) 1/60 |
|  |  | 2B6V.00 | 2163964014 | O/E - R-eye visual acuity (corrected) 2/60 |
|  |  | 2B6W.00 | 2163965010 | O/E - R-eye visual acuity (corrected) 4/60 |
|  |  | 2B6X.00 | 2163966011 | O/E - R-eye visual acuity (corrected) 5/60 |
|  |  | 2B6Y.00 | 2163967019 | O/E - R-eye visual acuity (corrected) 6/3 |
|  |  | 2B7T.00 | 2163968012 | O/E - L-eye visual acuity (corrected) 1/60 |
|  |  | 2B7V.00 | 2163969016 | O/E - L-eye visual acuity (corrected) 2/60 |
|  |  | 2B7W.00 | 2163970015 | O/E - L-eye visual acuity (corrected) 4/60 |
|  |  | 2B7X.00 | 2163971016 | O/E - L-eye visual acuity (corrected) 5/60 |
|  |  | 2B7Y.00 | 2163972011 | O/E - L-eye visual acuity (corrected) 6/3 |
|  |  | F49..12 | 2164154014 | Low vision |
|  |  | F49..14 | 2164155010 | Sight impaired |
|  |  | F49..13 | 2164158012 | Partial sight |
|  |  | F490100 | 2619503012 | Both eyes total visual impairment |
|  |  | F48..00 | 61821000006117 | Visual disturbance |
|  |  | F481.00 | 124401000006112 | Subjective visual disturbance |
|  |  | F484.11 | 156141000006113 | Scotoma |
|  |  | F484.00 | 219351000000118 | Visual field defect |
|  |  | 2B92.00 | 271501000006111 | O/E - red-green colour blindness |
|  |  | F491.00 | 510181000006115 | Better eye: low vision, Lesser eye: profound visual impairment |
|  |  | F491500 | 510191000006117 | Better eye: moderate VI, Lesser eye: blind, unspecified |
|  |  | F492300 | 510201000006119 | Better eye: moderate VI, Lesser eye: low vision unspecified |
|  |  | F492500 | 510211000006116 | Better eye: moderate visual impairment, Lesser eye: moderate visual impairment |
|  |  | F491700 | 510221000006112 | Better eye: moderate visual impairment, Lesser eye: near total visual impairment |
|  |  | F492400 | 510241000006117 | Better eye: moderate visual impairment, Lesser eye: severe visual impairment |
|  |  | F490400 | 510261000006118 | Better eye: near total visual impairment, Lesser eye: near total visual impairment |
|  |  | F490200 | 510281000006111 | Better eye: near total VI, Lesser eye: unspecified |
|  |  | F490600 | 510311000006113 | Impairment level: better eye: profound impairment: lesser eye: total impairment |
|  |  | F491100 | 510331000006119 | Better eye: severe VI, Lesser eye: blind, unspecified |
|  |  | F491300 | 510351000006114 | Impairment level: better eye: severe impairment: lesser eye: near-total impairment |
|  |  | F491400 | 510361000006111 | Better eye: severe visual impairment, Lesser eye: profound visual impairment |
|  |  | F492200 | 510371000006116 | Better eye: severe visual impairment, Lesser eye: severe visual impairment |
|  |  | F49..00 | 515791000006112 | Blindness and low vision |
|  |  | F490.00 | 515811000006111 | Blindness - both eyes |
|  |  | F48y011 | 570481000006117 | Cloudy vision |
|  |  | F485.11 | 573891000006110 | Colour blindness |
|  |  | F48y012 | 631921000006112 | Dull vision NOS |
|  |  | 1B75.00 | 734661000006116 | Loss of vision |
|  |  | F495600 | 734951000006114 | Lesser eye: near total visual impairment, Better eye: normal vision |
|  |  | F496500 | 747311000006117 | Lesser eye: moderate visual impairment, Better eye: near normal vision |
|  |  | F496600 | 747321000006113 | Impairment level: one eye: moderate impairment: other eye: normal vision |
|  |  | F496400 | 747331000006111 | Lesser eye: moderate VI, Better eye: unspecified |
|  |  | F495500 | 747341000006118 | Lesser eye: near total visual impairment, Better eye: near normal vision |
|  |  | F495400 | 747351000006116 | Lesser eye: near total VI, Better eye: unspecified |
|  |  | F495800 | 747361000006119 | Lesser eye: profound visual impairment, Better eye: near normal vision |
|  |  | F496200 | 747391000006110 | Lesser eye: severe visual impairment, Better eye: near normal vision |
|  |  | F496300 | 747401000006112 | Lesser eye: severe visual impairment, Better eye: normal vision |
|  |  | F496100 | 747411000006110 | Lesser eye: severe VI, Better eye: unspecified |
|  |  | F495200 | 747421000006119 | Lesser eye: total visual impairment, Better eye: near normal vision |
|  |  | F495300 | 747431000006116 | Lesser eye: total visual impairment, Better eye: normal vision |
|  |  | 1B74.00 | 762051000006113 | Floaters in visual field |
|  |  | F485.99 | 883341000006116 | Colour vision deficiencies |
|  |  | F48z.99 | 883351000006119 | Visual disturbances NOS |
|  |  | F490.98 | 883361000006117 | Blind/low vision - both eyes |
|  |  | F490.99 | 883371000006112 | Blind - both eyes |
|  |  | F496.99 | 883381000006110 | Blind/low vision -one eye only |
|  |  | 2BC2.99 | 903081000006116 | Right Hemianopia |
|  |  | F493.99 | 988991000006111 | Blind/low vision - both eyes |
|  |  | 2B6d.00 | 1719841000000118 | O/E - visual acuity right eye = 6/7.5 |
|  |  | 2B6e.00 | 1720061000000113 | O/E - visual acuity right eye = 6/48 |
|  |  | F499.00 | 1816031000006118 | Mild binocular visual impairment |
|  |  | F49A.00 | 1816041000006111 | Blindness, monocular |
|  |  | 2B6f.00 | 2114271000000117 | O/E visual acuity right eye = 6/20 |
|  |  | 2B6g.00 | 2114431000000118 | O/E visual acuity right eye = 6/10 |
|  |  | 2B6h.00 | 2140901000000119 | O/E visual acuity right eye = 6/7 |
|  |  | F497.00 | 2168181000000116 | Severe visual impairment, binocular |
|  |  | F49B.00 | 2168191000000119 | Severe visual impairment, monocular |
|  |  | F498.00 | 2168201000000117 | Moderate visual impairment, binocular |
|  |  | F49C.00 | 2168211000000115 | Moderate visual impairment, monocular |
| Involuntary movements |  | 1B22.12 | 43698017 | Shaking |
|  |  | 2972 | 254923017 | O/E - choreiform movement |
|  |  | 2973 | 254924011 | O/E - athetosis |
|  |  | 2974 | 254925012 | O/E - spasm/tic |
|  |  | 2974.12 | 254926013 | O/E - tic |
|  |  | 2974.11 | 254927016 | O/E - spasm |
|  |  | 2975 | 254928014 | O/E - fine tremor |
|  |  | 2976 | 254929018 | O/E - coarse tremor - flapping |
|  |  | 2976.12 | 254930011 | O/E - flapping tremor |
|  |  | 2976.11 | 254931010 | O/E - coarse tremor |
|  |  | 2977 | 254932015 | O/E - intention tremor |
|  |  | 2979 | 254934019 | O/E - myoclonus |
|  |  | 297Z.00 | 254936017 | O/E - involuntary movement NOS |
|  |  | 1B23.00 | 397971016 | Trembles |
|  |  | 1B24.00 | 397972011 | Has a tic |
|  |  | 1B25.00 | 397973018 | Has 'spasms' |
|  |  | 1B22.00 | 483107017 | Has a tremor |
|  |  | 1B23.11 | 87011000006112 | Trembles - symptom |
|  |  | 1B22.11 | 87021000006116 | Tremor |
|  |  | 1B24.11 | 102161000006115 | Tic |
|  |  | 1B25.11 | 132631000006111 | Spasms - symptom |
| Muscle weakness |  | F2A..00 | 33703013 | Hemiparesis |
|  |  | F22..00 | 84272011 | Hemiplegia |
|  |  | 1B38.00 | 87459019 | Motor dysfunction |
|  |  | F221.00 | 132112014 | Spastic hemiplegia |
|  |  | F220.00 | 134251013 | Flaccid hemiplegia |
|  |  | 1B32300 | 158479016 | Facial weakness |
|  |  | 1B32.00 | 252995016 | Weakness present |
|  |  | 1B33.00 | 253000011 | Paralysis present |
|  |  | 1B3Z.00 | 253004019 | Motor symptom NOS |
|  |  | 283..00 | 254824010 | O/E - paralysis |
|  |  | 2832 | 254826012 | O/E - paresis (weakness) |
|  |  | 2832.12 | 254827015 | O/E - weakness |
|  |  | 2832.11 | 254828013 | O/E - paresis |
|  |  | 2833 | 254829017 | O/E - hemiplegia |
|  |  | 2834 | 254830010 | O/E - monoplegia |
|  |  | 2835 | 254833012 | O/E - paraplegia |
|  |  | 2836 | 254834018 | O/E - quadriplegia |
|  |  | 2837 | 254835017 | O/E - diplegia |
|  |  | 283Z.00 | 254836016 | O/E - paralysis NOS |
|  |  | 2942 | 254899013 | O/E - muscle tone hypertonic |
|  |  | 2943 | 254901016 | O/E - muscle rigid-clasp knife |
|  |  | 2945 | 254904012 | O/E - muscle tone hypotonic |
|  |  | 2946 | 254905013 | O/E - muscle tone atonic |
|  |  | 2946.11 | 254906014 | O/E - atonic muscle |
|  |  | 2948 | 254908010 | O/E - muscle tone spastic |
|  |  | 2992 | 254948019 | O/E - gait spastic |
|  |  | 2992.11 | 254949010 | O/E - spastic gait |
|  |  | 2996 | 254957013 | O/E - steppage gait |
|  |  | 2998 | 254959011 | O/E - limping gait |
|  |  | 29A2.00 | 254965011 | O/E - muscle power reduced |
|  |  | 29A3.00 | 254966012 | O/E - muscle power unequal |
|  |  | 29A4.00 | 254967015 | O/E - muscle power absent |
|  |  | F22z.00 | 297210016 | Hemiplegia NOS |
|  |  | 1B32000 | 372825010 | Weakness of arm |
|  |  | 1B32100 | 372826011 | Weakness of leg |
|  |  | 2932 | 402510012 | O/E - muscle atrophy present |
|  |  | 2932.11 | 411815016 | O/E- quadriceps muscle wasting |
|  |  | 294..11 | 411831012 | O/E - rigid muscle |
|  |  | F223.00 | 415146010 | Right hemiplegia |
|  |  | F222.00 | 415147018 | Left hemiplegia |
|  |  | F223.11 | 415149015 | Right sided weakness |
|  |  | F222.11 | 415152011 | Left sided weakness |
|  |  | F221.11 | 450556015 | Spastic foot |
|  |  | 2949 | 1488401018 | Lower limb spasticity |
|  |  | 294A.00 | 1488402013 | Upper limb spasticity |
|  |  | 1B37.00 | 2476475010 | Loss of power in limb |
|  |  | F2Az.00 | 302151000000117 | Hemiparesis NOS |
|  |  | 1B32200 | 460931000006111 | Adbominal weakness |
|  |  | F22..11 | 820131000006114 | Hemiparesis |
|  |  | 294B.00 | 1747851000000119 | Worsening limb spasticity |
|  |  | 294C.00 | 1749601000000110 | Muscle flaccidity improving |
|  |  | 294E.00 | 1751911000000117 | Flaccidity of muscle of upper limb |
| Sensory symptoms |  | 29B6.00 | 24950014 | Hemisensory loss |
|  |  | 1B48.00 | 60126016 | Burning feet |
|  |  | 1B47.00 | 216639013 | Transient paraesthesia |
|  |  | 1B42.00 | 253007014 | Has prickling sensation |
|  |  | 1B43.00 | 253008016 | Has tingling sensation |
|  |  | 1B44.00 | 253009012 | Numbness |
|  |  | 1B4Z.00 | 253020012 | Sensory symptom NOS |
|  |  | 29B2000 | 254977018 | O/E - anaesthesia in legs |
|  |  | 29B2100 | 254980017 | O/E - anaesthesia of extremities |
|  |  | 29B3.00 | 254984014 | O/E - hypoaesthesia present |
|  |  | 29B5.00 | 254992017 | O/E - paraesthesia present |
|  |  | 29B5000 | 254993010 | O/E - paraesthesia in hands |
|  |  | 29C2.00 | 255001013 | O/E - tactile discrimination abnormal |
|  |  | 29E2.00 | 255018012 | O/E - pain sensation reduced |
|  |  | 29E3.00 | 255019016 | O/E - analgesia present |
|  |  | 29F2.00 | 255023012 | On examination - temperature discrimination reduced |
|  |  | 29F3.00 | 255024018 | On examination - temperature discrimination absent |
|  |  | 29B2.00 | 402516018 | O/E - anaesthesia present |
|  |  | 29B4.00 | 402519013 | O/E - hyperaesthesia present |
|  |  | 29B4.11 | 411824013 | O/E - hyperalgesia present |
|  |  | 29B2.11 | 411825014 | O/E - loss of touch sensation |
|  |  | 29C3.00 | 451379012 | Pin prick sensation of right foot abnormal |
|  |  | 29C4.00 | 451380010 | Pin prick sensation of left foot abnormal |
|  |  | 1B46.00 | 454065012 | C/O paraesthesia |
|  |  | 1B44200 | 454088013 | Numbness of limbs |
|  |  | 29C7.00 | 459217018 | Tactile discrimination right foot abnormal |
|  |  | 29C8.00 | 459235010 | Tactile discrimination left foot abnormal |
|  |  | 1B44000 | 476557016 | Hemianaesthesia |
|  |  | 1B49.00 | 1488779016 | Sensory disturbance in limb |
|  |  | 1B41.00 | 817031000006115 | Pins and needles |
|  |  | 29E4.00 | 1173331000000118 | On examination - allodynia |
| Trigeminal damage |  | F300.00 | 30355011 | Post-herpetic trigeminal neuralgia |
|  |  | F301000 | 52935016 | Tic douloureux |
|  |  | F302.00 | 118426010 | Atypical face pain |
|  |  | 2BR3.00 | 255471018 | O/E - cranial nerve 5 - palsy |
|  |  | F301.00 | 297427010 | Trigeminal neuralgia |
|  |  | F301z00 | 297428017 | Trigeminal neuralgia NOS |
|  |  | F30..00 | 87721000006119 | Trigeminal nerve disorder |
| Vertigos |  | F560.11 | 17117015 | Endolymphatic hydrops |
|  |  | F563.00 | 40171019 | Labyrinthitis |
|  |  | F561500 | 178784013 | Benign paroxysmal positional vertigo |
|  |  | F560000 | 299159019 | Menieres disease |
|  |  | F560100 | 299160012 | Active cochleovestibular Meniere's disease |
|  |  | F560200 | 299161011 | Active cochlear Meniere's disease |
|  |  | F560300 | 299162016 | Active vestibular Meniere's disease |
|  |  | F560400 | 299163014 | Inactive Meniere's disease |
|  |  | F560z00 | 299164015 | MÃƒÂ©niÃƒÂ¨re's disease |
|  |  | F561.00 | 299165019 | Other and unspecified peripheral vertigo |
|  |  | F561000 | 299166018 | Peripheral vertigo |
|  |  | F561100 | 299167010 | Benign paroxysmal positional vertigo or nystagmus |
|  |  | F561200 | 299168017 | Acute vestibular neuronitis |
|  |  | F561z00 | 299171013 | Other peripheral vertigo NOS |
|  |  | F562000 | 299172018 | Central positional nystagmus |
|  |  | F562100 | 299173011 | Malignant positional vertigo |
|  |  | F562z00 | 299174017 | Vertigo of central origin NOS |
|  |  | F563000 | 299175016 | Unspecified labyrinthitis |
|  |  | F563z00 | 299182017 | Labyrinthitis NOS |
|  |  | F565z00 | 299194014 | Labyrinthine dysfunction NOS |
|  |  | F56y.00 | 299196011 | Other labyrinth disorders |
|  |  | F56z.00 | 299197019 | Labyrinthine disorder |
|  |  | FyuQ100 | 299544019 | [X]Other peripheral vertigo |
|  |  | FyuQ200 | 299545018 | [X]Other disorders of vestibular function |
|  |  | FyuQ400 | 299547014 | [X]Disorder of vestibular function, unspecified |
|  |  | R004300 | 317028016 | [D]Vertigo NOS |
|  |  | R004400 | 317029012 | [D]Acute vertigo |
|  |  | F561300 | 478043019 | Recurrent vestibular neuronitis |
|  |  | F562.00 | 480691012 | Vertigo of central origin |
|  |  | F563500 | 2471473019 | Viral labyrinthitis |
|  |  | F56..00 | 67551000006116 | Disorder of inner ear |
|  |  | F56X.00 | 624251000006114 | Dysfunction of vestibular system |
|  |  | F560.00 | 710871000006110 | Meniere disease |
|  |  | F561299 | 883791000006112 | Vestibular neuronitis |
|  |  | F562.99 | 883801000006113 | Central nystagmus/vertigo |
|  |  | F56y.99 | 883811000006111 | Labyrinthine disorders NOS |

| 2e: Diagnostic readcodes and medcodes for pain symptoms. | | | | |
| --- | --- | --- | --- | --- |
| Symptom type | Subtype | ReadCode | MedCode | Term |
| Back neck pain |  | N135z12 | 116373011 | Wry neck |
|  |  | 16C8.00 | 216625013 | Exacerbation of backache |
|  |  | 16A2.00 | 252296010 | Stiff neck |
|  |  | 16AZ.00 | 252303013 | Stiff neck symptom NOS |
|  |  | 16C2.00 | 252311015 | Backache |
|  |  | 16C5.00 | 252314011 | C/O - low back pain |
|  |  | 16C6.00 | 252315012 | Back pain without radiation NOS |
|  |  | 16C7.00 | 252316013 | C/O - upper back ache |
|  |  | 16CZ.00 | 252317016 | Backache symptom NOS |
|  |  | N135z11 | 372797015 | Stiff neck NOS |
|  |  | N135z00 | 400281010 | Torticollis |
|  |  | N145.00 | 400286017 | Backache, unspecified |
|  |  | 16A3.00 | 405083016 | Wry neck/torticollis |
|  |  | 16C9.00 | 415888015 | Chronic low back pain |
|  |  | 16CA.00 | 416146018 | Mechanical low back pain |
|  |  | 16A3.12 | 54781000006115 | Wry neck symptom |
|  |  | N131.00 | 215271000000119 | Neck pain |
|  |  | 16A3.11 | 215431000000115 | Torticollis - symptom |
|  |  | N145.11 | 455801000006111 | Acute back pain - unspecified |
|  |  | N145.12 | 501991000006110 | Back pain |
|  |  | 16C..00 | 502061000006113 | Backache symptom |
|  |  | N145.99 | 890991000006119 | Backache NOS |
|  |  | N138.00 | 1682441000006112 | Cervicalgia |
|  |  | ^ESCTCO454518 | 4545181000006114 | Complaining of low back pain |
|  |  | ^ESCTCO454520 | 4545201000006110 | Complaining of upper back ache |
|  |  | ^ESCTLB562068 | 5620681000006112 | LBP - Low back pain |
| Eye pain |  | 1B84.11 | 69485018 | Pain in eye |
|  |  | F4Kz100 | 298923019 | Eye pain |
|  |  | 1B84.00 | 816931000006119 | Has eye pain |
| Headache |  | 1BA6.00 | 1634018 | Occipital headache |
|  |  | F260.11 | 7595017 | Migraine with aura |
|  |  | F260.00 | 7596016 | Migraine with typical aura |
|  |  | 1BA9.00 | 9300010 | Sinus headache |
|  |  | 1B1G.00 | 41990019 | Headache |
|  |  | 1B1G.12 | 41994011 | Cephalgia |
|  |  | F26..00 | 63055014 | Migraine |
|  |  | 1BA8.00 | 69073019 | Temporal headache |
|  |  | F261.11 | 93294013 | Migraine without aura |
|  |  | F261.00 | 93295014 | Common migraine |
|  |  | F261000 | 93296010 | Atypical migraine |
|  |  | F262700 | 158459012 | Chronic paroxysmal hemicrania |
|  |  | 1BA3.00 | 166489012 | Unilateral headache |
|  |  | 1BA2.00 | 253105015 | Generalised headache |
|  |  | 1BA4.00 | 253109014 | Bilateral headache |
|  |  | 1BA7.00 | 253115014 | Parietal headache |
|  |  | 1BAZ.00 | 253116010 | Headache site NOS |
|  |  | 1BB1.00 | 253118011 | Aching headache |
|  |  | 1BB2.00 | 253119015 | Throbbing headache |
|  |  | 1BB3.00 | 253120014 | Shooting headache |
|  |  | 1BB4.00 | 253121013 | Morning headache |
|  |  | 1BB5.00 | 253122018 | Heavy head |
|  |  | 1BBZ.00 | 253123011 | Finding of headache character |
|  |  | F261z00 | 297336013 | Common migraine NOS |
|  |  | F262.00 | 297337016 | Migraine variants |
|  |  | F262000 | 297338014 | Cluster headache syndrome |
|  |  | F262z00 | 297351016 | Migraine variant NOS |
|  |  | F26y.00 | 297352011 | Other forms of migraine |
|  |  | F26y300 | 297354012 | Complicated migraine |
|  |  | F26yz00 | 297355013 | Other forms of migraine NOS |
|  |  | F26z.00 | 297356014 | Migraine NOS |
|  |  | Fyu5300 | 299338017 | [X]Other migraine |
|  |  | Fyu5B00 | 299347013 | Drug induced headache |
|  |  | F26y200 | 345355014 | Status migrainosus |
|  |  | F262B00 | 345361012 | Chronic tension-type headache |
|  |  | 1BA5.00 | 397993014 | Frontal headache |
|  |  | 1B1G.11 | 407069017 | C/O - a headache |
|  |  | F262C00 | 416176013 | Short-lasting unilateral neuralgiform headache attacks with conjunctival injection and tearing syndrome |
|  |  | F262D00 | 2838283014 | Paroxysmal hemicrania |
|  |  | 1BA5.11 | 217741000000112 | Sinus headache |
|  |  | F262600 | 359201000006116 | Tension-type headache |
|  |  | Fyu5D00 | 1715221000006117 | Cervicogenic headache |
|  |  | Fyu5E00 | 1716421000006114 | Chronic headache disorder |
|  |  | F262800 | 1746951000000114 | Migraine induced by oestrogen contraceptive |
|  |  | F262900 | 1754211000000113 | Infrequent episodic tension-type headache |
|  |  | F262A00 | 1756651000000117 | Frequent episodic tension-type headache |
|  |  | F262E00 | 1757331000000118 | Trigeminal autonomic cephalalgia |
|  |  | ^ESCTCL257016 | 2570161000006115 | Classical migraine |
|  |  | ^ESCTCL476983 | 4769831000006110 | Cluster headache |
|  |  | ^ESCTCO553280 | 5532801000006112 | Complaining of a headache |
|  |  | ^ESCT1234532 | 12345321000006119 | Chronic migraine |
| Limb pain |  | N245.16 | 18423015 | Leg pain |
|  |  | 1M10.00 | 51875014 | Knee pain |
|  |  | 1M01.00 | 94135011 | Pain in wrist |
|  |  | 1M00.00 | 123425013 | Pain in elbow |
|  |  | 1M00.11 | 123426014 | Elbow pain |
|  |  | N245.00 | 150521017 | Pain in limb |
|  |  | N245.12 | 165844010 | Arm pain |
|  |  | 1M0..00 | 251425018 | Pain in upper limb |
|  |  | 1M1..00 | 297707010 | Pain in lower limb |
|  |  | 1M12.00 | 359208019 | Anterior knee pain |
|  |  | 1M13.00 | 369388017 | Ankle pain |
|  |  | 1M11.00 | 494960013 | Foot pain |
|  |  | N245000 | 496539011 | Hand pain |
|  |  | N245200 | 221731000000119 | Pain in leg |
|  |  | N245300 | 248531000006112 | Pain in arm |
|  |  | N245.11 | 483791000006115 | Ankle pain |
|  |  | N094W00 | 486291000006115 | Anterior knee pain |
|  |  | N245.13 | 764321000006112 | Foot pain |
|  |  | N245100 | 764331000006110 | Foot pain |
|  |  | N245.14 | 815651000006110 | Hand pain |
|  |  | N245.94 | 891871000006117 | Pain in limb NOS |
|  |  | N245.95 | 891881000006119 | Pain in right leg |
|  |  | N245.96 | 891891000006116 | Pain in left leg |
|  |  | N245.97 | 891901000006117 | Pain in right arm |
|  |  | N245.98 | 891911000006119 | Pain in left arm |
|  |  | N245.99 | 891921000006110 | Pain in limb - multiple |
|  |  | ^ESCTPA572183 | 5721831000006111 | Pain in left lower limb |
|  |  | ^ESCTPA572185 | 5721851000006116 | Pain in right lower limb |

| 2f: Diagnostic readcodes and medcodes for psychiatric symptoms. | | | | |
| --- | --- | --- | --- | --- |
| Symptom type | Subtype | ReadCode | MedCode | Term |
| Anxiety |  | 1B1H.12 | 83231016 | Apprehension |
|  |  | E200000 | 294953016 | Anxiety state unspecified |
|  |  | E200400 | 294960010 | Chronic anxiety |
|  |  | E200500 | 294961014 | Recurrent anxiety |
|  |  | E200z00 | 294963012 | Anxiety state |
|  |  | E203.11 | 295011010 | Anancastic neurosis |
|  |  | E203000 | 295015018 | Compulsive neurosis |
|  |  | E203100 | 295016017 | Obsessional neurosis |
|  |  | E203z00 | 295017014 | Obsessive-compulsive disorder NOS |
|  |  | Eu40.00 | 296224013 | [X]Phobic anxiety disorders |
|  |  | Eu40y00 | 296236019 | [X]Other phobic anxiety disorders |
|  |  | Eu40z00 | 296237011 | [X]Phobic anxiety disorder, unspecified |
|  |  | Eu41.00 | 296238018 | [X]Other anxiety disorders |
|  |  | Eu41000 | 296239014 | [X]Panic disorder [episodic paroxysmal anxiety] |
|  |  | Eu41300 | 296245018 | [X]Other mixed anxiety disorders |
|  |  | Eu41z00 | 296249012 | [X]Anxiety disorder, unspecified |
|  |  | Eu42000 | 296251011 | [X]Predominantly obsessional thoughts or ruminations |
|  |  | Eu42100 | 296252016 | Compulsion expressed as ritual |
|  |  | Eu42200 | 296253014 | [X]Mixed obsessional thoughts and acts |
|  |  | Eu42y00 | 296254015 | [X]Other obsessive-compulsive disorders |
|  |  | Eu42z00 | 296255019 | [X]Obsessive-compulsive disorder, unspecified |
|  |  | E200.99 | 304838011 | Anxiety state |
|  |  | E200111 | 339044013 | Panic attack |
|  |  | 1B14.00 | 392209017 | Tenseness |
|  |  | Eu41y00 | 401881014 | [X]Other specified anxiety disorders |
|  |  | E200200 | 481154010 | Generalised anxiety disorder |
|  |  | 1B16.00 | 481850015 | Agitated |
|  |  | E200100 | 1210253015 | Panic disorder |
|  |  | 1B1U.00 | 1488626018 | Symptoms of depression |
|  |  | 1B1V.00 | 1488717011 | C/O - panic attack |
|  |  | 1B1U.11 | 1494612017 | Depressive symptoms |
|  |  | 1B13.00 | 2536376012 | Anxiousness |
|  |  | 1B14.11 | 109271000006112 | Tenseness - symptom |
|  |  | 1B12.12 | 109281000006110 | Nervous tension |
|  |  | 1B1L.00 | 121881000006115 | Stress-related problem |
|  |  | 1B1O.00 | 168971000006110 | Restlessness |
|  |  | E203.00 | 269821000006113 | Obsessive-compulsive disorder |
|  |  | 1B12.11 | 291881000006114 | 'Nerves' |
|  |  | 1B12.00 | 291891000006112 | Nervousness |
|  |  | Eu40211 | 362181000006117 | [X]Acrophobia |
|  |  | Eu40000 | 362871000006111 | Agoraphobia |
|  |  | Eu40011 | 362881000006114 | [X]Agoraphobia without history of panic disorder |
|  |  | Eu42.11 | 363211000006112 | [X]Anankastic neurosis |
|  |  | Eu40212 | 363281000006117 | [X]Animal phobias |
|  |  | Eu41y11 | 363641000006114 | [X]Anxiety hysteria |
|  |  | Eu41111 | 363651000006111 | Anxiety neurosis |
|  |  | Eu41z11 | 363661000006113 | [X]Anxiety NOS |
|  |  | Eu41112 | 363671000006118 | [X]Anxiety reaction |
|  |  | Eu41113 | 363681000006115 | [X]Anxiety state |
|  |  | Eu40213 | 371071000006117 | [X]Claustrophobia |
|  |  | Eu41100 | 388071000006116 | [X]Generalized anxiety disorder |
|  |  | Eu41211 | 398351000006110 | [X]Mild anxiety depression |
|  |  | Eu41200 | 398561000006117 | [X]Mixed anxiety and depressive disorder |
|  |  | Eu42.00 | 400321000006113 | [X]Obsessive - compulsive disorder |
|  |  | Eu42.12 | 400341000006118 | [X]Obsessive-compulsive neurosis |
|  |  | Eu41011 | 418031000006113 | [X]Panic attack |
|  |  | Eu40012 | 418051000006118 | [X]Panic disorder with agoraphobia |
|  |  | Eu41012 | 418061000006116 | [X]Panic state |
|  |  | Eu40z11 | 420631000006114 | Phobic disorder |
|  |  | Eu40z12 | 420671000006112 | [X]Phobic state NOS |
|  |  | Eu40214 | 426871000006113 | [X]Simple phobia |
|  |  | Eu40112 | 427061000006110 | [X]Social neurosis |
|  |  | Eu40100 | 427071000006115 | Social phobia |
|  |  | Eu40200 | 427231000006111 | Specific phobia |
|  |  | 1B16.11 | 474161000006117 | Agitated - symptom |
|  |  | E200.00 | 488201000006114 | Anxiety disorder |
|  |  | E200300 | 488211000006112 | Mixed anxiety and depressive disorder |
|  |  | 1B13.11 | 488251000006113 | Anxiousness - symptom |
|  |  | 1B13.12 | 516601000000113 | Anxious |
|  |  | 1B15.00 | 742321000006111 | Feeling irritable |
|  |  | 1B15.11 | 745571000006112 | Irritable - symptom |
|  |  | Eu40299 | 932131000006118 | Needle phobia |
|  |  | Eu40300 | 980191000006111 | Needle phobia |
|  |  | 1B13-100 | 1818111000006112 | Feeling anxious |
|  |  | ^ESCTGE284829 | 2848291000006116 | Generalized anxiety disorder |
|  |  | ^ESCTGA284830 | 2848301000006115 | GAD - Generalized anxiety disorder |
|  |  | ^ESCTGA284831 | 2848311000006117 | GAD - Generalised anxiety disorder |
|  |  | ^ESCTAN328774 | 3287741000006111 | Anxiety |
|  |  | ^ESCTFE328776 | 3287761000006110 | Feeling anxious |
|  |  | ^ESCTMO349737 | 3497371000006119 | Moderate anxiety |
|  |  | ^ESCTMI365468 | 3654681000006114 | Mild anxiety |
|  |  | ^ESCTNO502403 | 5024031000006116 | Non-situational panic attack |
|  |  | ^ESCTAN588648 | 5886481000006112 | Anxiety attack |
| Depression |  | 1Ba2.00 | 13594019 | Thought retardation |
|  |  | 1BX9.00 | 49145012 | Light sleep |
|  |  | 2257 | 253619019 | O/E - depressed |
|  |  | E112000 | 294824018 | Single major depressive episode, unspecified |
|  |  | E112100 | 294825017 | Mild major depression, single episode |
|  |  | E112200 | 294826016 | Moderate major depression, single episode |
|  |  | E112400 | 294828015 | Single major depressive episode, severe, with psychosis |
|  |  | E112600 | 294831019 | Single episode of major depression in full remission |
|  |  | E112z00 | 294832014 | Single major depressive episode NOS |
|  |  | E113000 | 294836012 | Recurrent major depressive episodes, unspecified |
|  |  | E113100 | 294837015 | Recurrent major depressive episodes, mild |
|  |  | E113200 | 294838013 | Recurrent major depressive episodes, moderate |
|  |  | E113400 | 294840015 | Recurrent major depressive episodes, severe, with psychosis |
|  |  | E113600 | 294843018 | Recurrent major depression in full remission |
|  |  | E113700 | 294844012 | Recurrent depression |
|  |  | E113z00 | 294845013 | Recurrent major depressive episode NOS |
|  |  | E11y200 | 294894013 | Atypical depressive disorder |
|  |  | E2B..00 | 295535012 | Depressive disorder |
|  |  | E2B1.00 | 295537016 | Chronic depression |
|  |  | Eu32000 | 296137015 | Mild depression |
|  |  | Eu32100 | 296138013 | [X]Moderate depressive episode |
|  |  | Eu33000 | 296180012 | [X]Recurrent depressive disorder, current episode mild |
|  |  | Eu33100 | 296181011 | [X]Recurrent depressive disorder, current episode moderate |
|  |  | Eu33400 | 296198011 | [X]Recurrent depressive disorder, currently in remission |
|  |  | Eu33y00 | 296199015 | [X]Other recurrent depressive disorders |
|  |  | E112.13 | 346972018 | Endogenous depression first episode |
|  |  | E11z200 | 346973011 | Masked depression |
|  |  | 1BP..00 | 369915018 | Loss of interest |
|  |  | 1B1P000 | 369940013 | Cries easily |
|  |  | 1BQ..00 | 369972011 | Loss of capacity for enjoyment |
|  |  | 1BU..00 | 369974012 | Feeling hopeless |
|  |  | 1BF..11 | 370104015 | Pathological guilt |
|  |  | 1B1K.00 | 397966011 | Lonely |
|  |  | 1B1K.11 | 397967019 | Loneliness |
|  |  | 1BF..00 | 397994015 | Guilty ideas |
|  |  | E112.00 | 401766011 | Major depression, single episode |
|  |  | Eu32200 | 401866015 | [X]Severe depressive episode without psychotic symptoms |
|  |  | Eu32300 | 401869010 | [X]Severe depressive episode with psychotic symptoms |
|  |  | Eu32y00 | 401871010 | [X]Other depressive episodes |
|  |  | Eu32z00 | 401872015 | [X]Depressive episode, unspecified |
|  |  | Eu33.00 | 401873013 | [X]Recurrent depressive disorder |
|  |  | Eu33z00 | 401876017 | [X]Recurrent depressive disorder, unspecified |
|  |  | 1B17.11 | 407062014 | C/O - feeling depressed |
|  |  | 1B17.12 | 407066012 | C/O - feeling unhappy |
|  |  | E113.11 | 410861011 | Endogenous depression - recurrent |
|  |  | E112.14 | 441826016 | Endogenous depression |
|  |  | 1B1P.00 | 446851016 | Crying |
|  |  | 1B1U.00 | 1488626018 | Symptoms of depression |
|  |  | 1B1U.11 | 1494612017 | Depressive symptoms |
|  |  | 1BX3.00 | 1780430015 | Early morning waking |
|  |  | 1BT..00 | 2164005017 | Depressed mood |
|  |  | 1B17.00 | 2164006016 | Depressed |
|  |  | 1BT..11 | 2164009011 | Low mood |
|  |  | 1BP0.00 | 2548573012 | Loss of interest in previously enjoyable activity |
|  |  | E112500 | 142521000006110 | Major depression single episode, in partial remission |
|  |  | E112300 | 142541000006115 | Severe major depression, single episode |
|  |  | E113.00 | 182721000006111 | Recurrent major depressive episodes |
|  |  | E113300 | 182771000006112 | Recurrent major depressive episodes, severe |
|  |  | E113500 | 182801000006114 | Recurrent major depressive episodes,partial/unspec remission |
|  |  | Eu32400 | 213641000000111 | [X]Mild depression |
|  |  | Eu32212 | 223741000000112 | Severe depression |
|  |  | Eu32z14 | 359121000006116 | Reactive depression |
|  |  | Eu32y11 | 366561000006119 | [X]Atypical depression |
|  |  | Eu32z11 | 376691000006116 | Depression |
|  |  | Eu32z12 | 376711000006118 | [X]Depressive disorder NOS |
|  |  | Eu32.00 | 376721000006114 | Depressive episode |
|  |  | Eu34111 | 376741000006119 | [X]Depressive neurosis |
|  |  | Eu34112 | 376751000006117 | Depressive personality disorder |
|  |  | Eu34100 | 379431000006113 | Dysthymia |
|  |  | Eu33311 | 379771000006116 | [X]Endogenous depression with psychotic symptoms |
|  |  | Eu33211 | 379781000006118 | [X]Endogenous depression without psychotic symptoms |
|  |  | Eu33212 | 396081000006116 | [X]Major depression, recurrent without psychotic symptoms |
|  |  | Eu33213 | 396771000006117 | [X] Manic-depressive psychosis, depressed type without psychotic symptoms |
|  |  | Eu33312 | 396781000006119 | [X]Manic-depress psychosis,depressed type+psychotic symptoms |
|  |  | Eu33z11 | 398841000006119 | [X]Monopolar depression NOS |
|  |  | Eu34113 | 399961000006118 | Depressive neurosis |
|  |  | Eu32z13 | 423611000006111 | [X]Prolonged single episode of reactive depression |
|  |  | Eu33200 | 424531000006118 | [X]Recurr depress disorder cur epi severe without psyc sympt |
|  |  | Eu33313 | 424541000006111 | [X]Recurr severe episodes/major depression+psychotic symptom |
|  |  | Eu33314 | 424551000006113 | [X]Recurr severe episodes/psychogenic depressive psychosis |
|  |  | Eu3y111 | 424561000006110 | [X]Recurrent brief depressive episodes |
|  |  | Eu33300 | 424571000006115 | [X]Recurrent depress disorder cur epi severe with psyc symp |
|  |  | Eu33.11 | 424631000006119 | [X]Recurrent episodes of depressive reaction |
|  |  | Eu33.12 | 424641000006112 | [X]Recurrent episodes of psychogenic depression |
|  |  | Eu33.13 | 424651000006114 | [X]Recurrent episodes of reactive depression |
|  |  | Eu33315 | 424671000006116 | [X]Recurrent severe episodes of psychotic depression |
|  |  | Eu33316 | 424681000006118 | Recurrent reactive depressive episodes, severe, with psychosis |
|  |  | Eu33.15 | 425411000006110 | SAD - Seasonal affective disorder |
|  |  | Eu33.14 | 425751000006115 | [X]Seasonal depressive disorder |
|  |  | Eu32211 | 426891000006114 | [X] Single episode agitated depression without psychotic symptoms |
|  |  | Eu32.11 | 426911000006111 | [X]Single episode of depressive reaction |
|  |  | Eu32311 | 426921000006115 | [X]Single episode of major depression and psychotic symptoms |
|  |  | Eu32y12 | 426931000006117 | [X]Single episode of masked depression NOS |
|  |  | Eu32.12 | 426941000006110 | [X]Single episode of psychogenic depression |
|  |  | Eu32312 | 426951000006112 | [X]Single episode of psychogenic depressive psychosis |
|  |  | Eu32313 | 426961000006114 | [X]Single episode of psychotic depression |
|  |  | Eu32.13 | 426971000006119 | [X]Single episode of reactive depression |
|  |  | Eu32314 | 426981000006116 | [X]Single episode of reactive depressive psychosis |
|  |  | Eu32213 | 426991000006118 | [X]Single episode vital depression w'out psychotic symptoms |
|  |  | Eu33214 | 432511000006119 | [X]Vital depression, recurrent without psychotic symptoms |
|  |  | E112.11 | 474171000006112 | Agitated depression |
|  |  | E112.12 | 642461000006116 | Endogenous depression first episode |
|  |  | E2B..98 | 882671000006112 | Depression |
|  |  | E2B..99 | 882681000006110 | Depression NOS |
|  |  | Eu32099 | 882811000006119 | Mild depression |
|  |  | Eu32199 | 882821000006110 | Moderate depression |
|  |  | Eu32299 | 882831000006113 | Severe depression |
|  |  | 1BT..12 | 983861000006115 | Sad mood |
|  |  | 16ZB100 | 1146851000000112 | howRu rating score - Feeling low or worried |
|  |  | 665A000 | 1166201000000117 | Antidepressant drug treatment stopped |
|  |  | Eu32600 | 1715181000006114 | Moderate major depression |
|  |  | Eu32800 | 1715191000006112 | Severe major depression with psychotic features |
|  |  | Eu32500 | 1715771000006112 | Mild major depression |
|  |  | Eu32700 | 1715781000006110 | Severe major depression without psychotic features |
|  |  | 6659000 | 1730231000000110 | Antidepressant drug treatment started |
|  |  | Eu32900 | 1755901000006112 | [X]Single major depressive episode, severe, with psychosis, psychosis in remission |
|  |  | Eu32A00 | 1755911000006110 | [X]Recurrent major depressive episodes, severe, with psychosis, psychosis in remission |
|  |  | Eu32B00 | 1785881000006119 | Antenatal depression |
|  |  | 6658000 | 2143511000000118 | Antidepressant drug treatment changed |
|  |  | ^ESCTDE307180 | 3071801000006112 | Depressed |
|  |  | ^ESCTCO553272 | 5532721000006114 | Complaining of feeling depressed |
|  |  | ^ESCTMO778866 | 7788661000006119 | Moderately severe depression |
| Eating disorders |  | E275200 | 23968015 | Pica |
|  |  | E271.00 | 94597012 | Anorexia nervosa |
|  |  | 1624 | 252194018 | Abnormal weight gain |
|  |  | E275.00 | 295436010 | Other and unspecified non-organic eating disorders |
|  |  | E275000 | 295437018 | Unspecified non-organic eating disorder |
|  |  | E275300 | 295441019 | Psychogenic rumination |
|  |  | E275400 | 295442014 | Psychogenic vomiting |
|  |  | E275800 | 295449017 | Specific food craving |
|  |  | E275y00 | 295451018 | Other specified non-organic eating disorder |
|  |  | Eu50.00 | 296361019 | Eating disorder |
|  |  | E275711 | 346989014 | Compulsive water drinking |
|  |  | 1612.11 | 372208017 | Anorexia symptom |
|  |  | 1614 | 397863019 | Excessive eating - polyphagia |
|  |  | 1625 | 397867018 | Abnormal weight loss |
|  |  | Eu50y00 | 401889011 | [X]Other eating disorders |
|  |  | 1612 | 404431014 | Appetite loss - anorexia |
|  |  | 1614.12 | 407104010 | Polyphagia symptom |
|  |  | 1615 | 499835012 | Reduced appetite |
|  |  | 1626 | 2549043017 | Intentional weight loss |
|  |  | Eu50000 | 363321000006111 | [X]Anorexia nervosa |
|  |  | Eu50100 | 366521000006113 | Atypical anorexia nervosa |
|  |  | Eu50300 | 366541000006118 | Atypical bulimia nervosa |
|  |  | Eu50200 | 368051000006111 | Bulimia nervosa |
|  |  | Eu50211 | 368061000006113 | [X]Bulimia NOS |
|  |  | Eu50212 | 389311000006116 | [X]Hyperorexia nervosa |
|  |  | Eu50400 | 417851000006113 | Overeating associated with other psychological disturbances |
|  |  | Eu50411 | 424061000006117 | [X]Psychogenic overeating |
|  |  | Eu50511 | 424151000006115 | [X]Psychogenic vomiting |
|  |  | Eu50500 | 432551000006118 | Vomiting associated with other psychological disturbances |
|  |  | 1625.11 | 437661000006118 | Abnormal weight loss - symptom |
|  |  | 1613 | 489641000006115 | Increased appetite |
|  |  | E275100 | 527421000006116 | Bulimia (non-organic overeating) |
|  |  | E275111 | 577901000006119 | Compulsive eating disorder |
|  |  | 1612.12 | 734511000006115 | Loss of appetite |
|  |  | 1614.11 | 830401000006110 | Hyperalimentation - symptom |
|  |  | E275.99 | 882611000006115 | Eating disorder NOS - psychog. |
|  |  | EMISCAN3 | 960281000006115 | Anorexia |
|  |  | E275099 | 988931000006112 | Eating disorder NOS - psychog. |
|  |  | EMISNQEA3 | 1620911000006112 | Eating disorders |
| GID |  | 7L0By00 | 278517011 | Other specified operation for sexual transformation |
|  |  | 7L0Bz00 | 278518018 | Operation for sexual transformation NOS |
|  |  | E22y400 | 295119010 | Gender role disorder of adolescent or adult |
|  |  | Eu64.00 | 296515015 | Gender identity disorder |
|  |  | Eu64y00 | 296518018 | [X]Other gender identity disorders |
|  |  | Eu64z00 | 296519014 | [X]Gender identity disorder, unspecified |
|  |  | 7L0B.00 | 45781000006119 | Operations for sexual transformation |
|  |  | Eu64100 | 379391000006119 | [X]Dual-role transvestism |
|  |  | Eu64200 | 388001000006110 | Gender identity disorder of childhood |
|  |  | Eu64z11 | 388031000006119 | [X]Gender-role disorder NOS |
|  |  | Eu64000 | 429301000006117 | [X]Transsexualism |
|  |  | 7L0B100 | 574451000006117 | Combined operations for transformation from female to male |
|  |  | 7L0B000 | 574461000006115 | Combined operations for transformation from male to female |
|  |  | 7L0Bz99 | 877261000006114 | Sex transformation operat. NOS |
| Insomnia |  | E274D11 | 21110011 | Restless sleep |
|  |  | 1B1B000 | 98133013 | Initial insomnia |
|  |  | 1B1B100 | 111701012 | Middle insomnia |
|  |  | 1B1B200 | 252935019 | Late insomnia |
|  |  | E274200 | 295412011 | Persistent insomnia |
|  |  | R005200 | 317035012 | [D]Insomnia NOS |
|  |  | E274111 | 345377013 | Insomnia NOS |
|  |  | E274100 | 401805019 | Transient insomnia |
|  |  | 1B1B.11 | 407067015 | Complaining of insomnia |
|  |  | 1B1Q.00 | 459355013 | Poor sleep pattern |
|  |  | R005.11 | 1222474014 | [D]Insomnia - symptom |
|  |  | 1BX0.00 | 1780361011 | Delayed onset of sleep |
|  |  | 1B1B.00 | 217721000000117 | Insomnia |
|  |  | Eu51000 | 400121000006111 | Nonorganic insomnia |
|  |  | E274.12 | 772561000006111 | Insomnia due to nonorganic sleep disorder |
|  |  | EMISCIN31 | 960621000006117 | Insomnia |
| Manic |  | 1BO..00 | 31960011 | Mood swings |
|  |  | 1BG..00 | 48238012 | Flight of ideas |
|  |  | 1S40.00 | 51590017 | Dysphoric mood |
|  |  | 1BY..00 | 135278017 | Elevated mood |
|  |  | 665B.00 | 264612016 | Lithium stopped |
|  |  | E110000 | 294802018 | Single manic episode, unspecified |
|  |  | E110100 | 294803011 | Single manic episode, mild |
|  |  | E110200 | 294804017 | Single manic episode, moderate |
|  |  | E110300 | 294805016 | Single manic episode, severe |
|  |  | E110400 | 294806015 | Single manic episode, severe, with psychosis |
|  |  | E110500 | 294807012 | Single manic episode in partial remission |
|  |  | E110600 | 294808019 | Single manic episode in full remission |
|  |  | E110z00 | 294809010 | Manic disorder, single episode NOS |
|  |  | E111.00 | 294810017 | Recurrent manic episodes |
|  |  | E111000 | 294811018 | Recurrent manic episodes, unspecified |
|  |  | E111100 | 294812013 | Recurrent manic episodes, mild |
|  |  | E111200 | 294813015 | Recurrent manic episodes, moderate |
|  |  | E111400 | 294815010 | Recurrent manic episodes, severe, with psychosis |
|  |  | E111600 | 294817019 | Recurrent manic episodes, in full remission |
|  |  | E111z00 | 294818012 | Recurrent manic episode NOS |
|  |  | E114.11 | 294847017 | Manic-depressive - now manic |
|  |  | E114000 | 294848010 | Bipolar affective disorder, currently manic, unspecified |
|  |  | E114100 | 294849019 | Bipolar affective disorder, currently manic, mild |
|  |  | E114200 | 294850019 | Bipolar affective disorder, currently manic, moderate |
|  |  | E114z00 | 294858014 | Bipolar affective disorder, currently manic, NOS |
|  |  | E115.11 | 294860011 | Manic-depressive - now depressed |
|  |  | E115000 | 294861010 | Bipolar affective disorder, currently depressed, unspecified |
|  |  | E115100 | 294862015 | Bipolar affective disorder, currently depressed, mild |
|  |  | E115200 | 294863013 | Bipolar affective disorder, currently depressed, moderate |
|  |  | E115z00 | 294868016 | Bipolar affective disorder, currently depressed, NOS |
|  |  | E116.00 | 294869012 | Mixed bipolar affective disorder |
|  |  | E116000 | 294870013 | Mixed bipolar affective disorder, unspecified |
|  |  | E116100 | 294871012 | Mixed bipolar affective disorder, mild |
|  |  | E116200 | 294872017 | Mixed bipolar affective disorder, moderate |
|  |  | E116400 | 294874016 | Mixed bipolar affective disorder, severe, with psychosis |
|  |  | E116600 | 294876019 | Mixed bipolar affective disorder, in full remission |
|  |  | E116z00 | 294877011 | Mixed bipolar affective disorder, NOS |
|  |  | E117.00 | 294880012 | Unspecified bipolar affective disorder |
|  |  | E117000 | 294881011 | Unspecified bipolar affective disorder, unspecified |
|  |  | E117100 | 294882016 | Mild bipolar disorder |
|  |  | E117200 | 294883014 | Moderate bipolar disorder |
|  |  | E117600 | 294887010 | Bipolar disorder in full remission |
|  |  | E117z00 | 294888017 | Unspecified bipolar affective disorder, NOS |
|  |  | E11y.00 | 294891017 | Other and unspecified manic-depressive psychoses |
|  |  | E11y000 | 294892012 | Unspecified manic-depressive psychoses |
|  |  | E11y100 | 294893019 | Atypical manic disorder |
|  |  | E11y300 | 294895014 | Mixed bipolar I disorder |
|  |  | E11yz00 | 294896010 | Other and unspecified manic-depressive psychoses NOS |
|  |  | E11z.00 | 294897018 | Affective psychosis |
|  |  | E11z000 | 294898011 | Unspecified affective psychoses NOS |
|  |  | E11z100 | 294899015 | Rebound mood swings |
|  |  | E11zz00 | 294902017 | Other affective psychosis NOS |
|  |  | Eu30100 | 296110017 | Mania |
|  |  | Eu30y00 | 296118012 | [X]Other manic episodes |
|  |  | Eu31700 | 296130018 | Bipolar disorder in remission |
|  |  | Eu31z00 | 296135011 | [X]Bipolar affective disorder, unspecified |
|  |  | 1S4..00 | 298986013 | Mood observations |
|  |  | 1BR0.00 | 369925011 | Reduced concentration span |
|  |  | 1BR0.11 | 369926012 | Short attention span |
|  |  | E118.00 | 369982012 | Seasonal affective disorder |
|  |  | 6657 | 401522012 | On lithium |
|  |  | 6657.12 | 401523019 | Started lithium |
|  |  | E110.00 | 401765010 | Manic disorder, single episode |
|  |  | Eu30200 | 401863011 | [X]Mania with psychotic symptoms |
|  |  | Eu30z00 | 401864017 | [X]Manic episode, unspecified |
|  |  | Eu31y00 | 401865016 | [X]Other bipolar affective disorders |
|  |  | 6657.11 | 411890010 | Lithium monitoring |
|  |  | 1S42.00 | 2157096015 | Manic mood |
|  |  | 1S41.00 | 2551254013 | Ecstatic mood |
|  |  | E117500 | 82091000006116 | Bipolar disorder in partial remission |
|  |  | E117300 | 82151000006114 | Severe bipolar disorder without psychotic features |
|  |  | E117400 | 82171000006116 | Severe bipolar disorder with psychotic features |
|  |  | E111500 | 182861000006110 | Recurrent manic episodes, in partial remission |
|  |  | E111300 | 182871000006115 | Recurrent manic episodes, severe |
|  |  | Eu31400 | 367051000006112 | Severe depressed bipolar I disorder without psychotic features |
|  |  | Eu31500 | 367061000006114 | Psychosis and severe depression co-occurrent and due to bipolar affective disorder |
|  |  | Eu31200 | 367071000006119 | Bipolar affective disorder, currently manic, severe, with psychosis |
|  |  | Eu31100 | 367081000006116 | [X]Bipolar affect disorder cur epi manic wout psychotic symp |
|  |  | Eu31300 | 367091000006118 | [X]Bipolar affect disorder cur epi mild or moderate depressn |
|  |  | Eu31.00 | 367101000006112 | Bipolar affective disorder |
|  |  | Eu31000 | 367111000006110 | Bipolar affective disorder, current episode hypomanic |
|  |  | Eu31600 | 367121000006119 | [X]Bipolar affective disorder, current episode mixed |
|  |  | Eu30.11 | 367151000006111 | [X]Bipolar disorder, single manic episode |
|  |  | Eu31y11 | 367161000006113 | [X]Bipolar II disorder |
|  |  | Eu34012 | 376261000006114 | [X]Cycloid personality |
|  |  | Eu34000 | 376291000006118 | Cyclothymia |
|  |  | Eu34013 | 376301000006117 | [X]Cyclothymic personality |
|  |  | Eu30000 | 389401000006111 | Hypomania |
|  |  | Eu31.13 | 396071000006119 | [X]Manic-depressive reaction |
|  |  | Eu30z11 | 396691000006111 | [X]Mania NOS |
|  |  | Eu30211 | 396701000006111 | [X]Mania with mood-congruent psychotic symptoms |
|  |  | Eu30212 | 396711000006114 | [X]Mania with mood-incongruent psychotic symptoms |
|  |  | Eu30.00 | 396741000006113 | [X]Manic episode |
|  |  | Eu31.11 | 396791000006116 | Manic-depressive illness |
|  |  | Eu31.12 | 396801000006115 | Manic-depressive psychosis |
|  |  | Eu31y12 | 424661000006111 | [X]Recurrent manic episodes |
|  |  | 212V.00 | 492761000000114 | Bipolar affective disorder resolved |
|  |  | E114300 | 513691000006116 | Severe manic bipolar I disorder without psychotic features |
|  |  | E114400 | 513701000006116 | Bipolar affect disord, currently manic,severe with psychosis |
|  |  | E115500 | 513711000006118 | Depressed bipolar I disorder in partial remission |
|  |  | E115400 | 513721000006114 | Bipolar affect disord, now depressed, severe with psychosis |
|  |  | E115300 | 513731000006112 | Bipolar affect disord, now depressed, severe, no psychosis |
|  |  | E114500 | 513741000006119 | Manic bipolar I disorder in partial remission |
|  |  | E115.00 | 513751000006117 | Bipolar affective disorder, current episode depression |
|  |  | E114.00 | 513801000006112 | Bipolar affective disorder, current episode manic |
|  |  | E114600 | 513811000006110 | Bipolar affective disorder, currently manic, in full remission |
|  |  | E115600 | 513861000006113 | Bipolar affective disorder, currently depressed, in full remission |
|  |  | E116500 | 701051000006118 | Mixed bipolar affective disorder, partial/unspec remission |
|  |  | E116300 | 701071000006111 | Mixed bipolar affective disorder, severe |
|  |  | E110.11 | 789221000006116 | Hypomanic psychoses |
|  |  | EMISQHY1 | 851691000006114 | Hypomanic |
|  |  | E110.99 | 882321000006119 | Mania/hypomania |
|  |  | EMISCMO2 | 981181000006117 | Mood swings |
|  |  | 665J.00 | 1662121000000117 | Lithium level checked at 3 monthly intervals |
|  |  | 665K.00 | 1667141000000114 | Lithium therapy record book completed |
|  |  | EMISNQPA105 | 1747721000006112 | Patient lithium therapy pack given |
|  |  | Eu31800 | 1785851000006110 | Bipolar I disorder |
|  |  | Eu31900 | 1785861000006112 | Bipolar II disorder |
|  |  | Eu31911 | 1785871000006117 | [X]Bipolar II disorder |
| Personality disorders |  | E210.00 | 23211011 | Paranoid personality disorder |
|  |  | E21y200 | 33677019 | Borderline personality disorder |
|  |  | E21y100 | 62962010 | Avoidant personality disorder |
|  |  | E212.00 | 88108012 | Schizoid personality disorder |
|  |  | E21y000 | 133885012 | Narcissistic personality disorder |
|  |  | E211000 | 295044016 | Affective personality disorder |
|  |  | E211100 | 295045015 | Hypomanic personality disorder |
|  |  | E211z00 | 295048018 | Affective personality disorder NOS |
|  |  | E212000 | 295049014 | Unspecified schizoid personality disorder |
|  |  | E212z00 | 295052018 | Schizoid personality disorder NOS |
|  |  | E214z00 | 295063015 | Compulsive personality disorder NOS |
|  |  | E215000 | 295064014 | Unspecified histrionic personality disorder |
|  |  | E215300 | 295066011 | Psychoinfantile personality |
|  |  | E215z00 | 295067019 | Histrionic personality disorder NOS |
|  |  | E21y.00 | 295077017 | Other personality disorders |
|  |  | E21y400 | 295079019 | Eccentric personality disorder |
|  |  | E21y500 | 295080016 | Immature personality disorder |
|  |  | E21y600 | 295081017 | Masochistic personality disorder |
|  |  | Eu60.00 | 296460017 | [X]Specific personality disorders |
|  |  | Eu60z00 | 296488011 | [X]Personality disorder, unspecified |
|  |  | E213.00 | 347011014 | Explosive personality disorder |
|  |  | E21y700 | 401785014 | Psychoneurotic personality disorder |
|  |  | E21yz00 | 401786010 | Other personality disorder NOS |
|  |  | E21z.00 | 401787018 | Personality disorder NOS |
|  |  | Eu60y00 | 401898014 | [X]Other specific personality disorders |
|  |  | E21yz11 | 453182018 | Manipulative personality |
|  |  | E214100 | 475487017 | Obsessional personality |
|  |  | E215100 | 481069014 | Munchausen's syndrome |
|  |  | E21y300 | 1230168015 | Passive-aggressive personality disorder |
|  |  | E211.00 | 1495439012 | Affective personality disorder |
|  |  | Eu60800 | 2160091013 | [X]Addictive personality |
|  |  | E212200 | 155281000006119 | Schizotypal personality |
|  |  | E213.12 | 195991000006117 | Quarrelsome personality |
|  |  | E21z.11 | 199821000006114 | Psychopathic personality |
|  |  | E216.12 | 223661000000115 | Dependent personality |
|  |  | E21..00 | 230091000006118 | Personality disorder |
|  |  | Eu60311 | 362851000006118 | [X]Aggressive personality disorder |
|  |  | Eu60211 | 363081000006112 | [X]Amoral personality disorder |
|  |  | Eu60500 | 363221000006116 | Anankastic personality disorder |
|  |  | Eu60212 | 363561000006112 | Antisocial personality disorder |
|  |  | Eu60600 | 363691000006117 | Anxious personality disorder |
|  |  | Eu60213 | 363921000006112 | [X]Asocial personality disorder |
|  |  | Eu60711 | 366421000006114 | [X]Asthenic personality disorder |
|  |  | Eu60312 | 367941000006111 | [X]Borderline personality disorder |
|  |  | Eu60511 | 371341000006110 | [X]Compulsive personality disorder |
|  |  | Eu60700 | 376671000006117 | Dependent personality disorder |
|  |  | Eu60200 | 378071000006112 | Dissocial personality disorder |
|  |  | Eu60y11 | 379511000006118 | [X]Eccentric personality disorder |
|  |  | Eu60300 | 379701000006110 | Emotionally unstable personality disorder |
|  |  | Eu60313 | 380611000006117 | [X]Explosive personality disorder |
|  |  | Eu60y12 | 388391000006117 | [X]Haltlose type personality disorder |
|  |  | Eu60400 | 388871000006111 | [X]Histrionic personality disorder |
|  |  | Eu60411 | 389441000006113 | [X]Hysterical personality disorder |
|  |  | Eu60y13 | 389491000006116 | [X]Immature personality disorder |
|  |  | Eu60712 | 389581000006115 | [X]Inadequate personality disorder |
|  |  | Eu60y14 | 399451000006114 | [X]Narcissistic personality disorder |
|  |  | Eu60512 | 400311000006117 | [X]Obsessional personality disorder |
|  |  | Eu60513 | 400351000006116 | [X]Obsessive-compulsive personality disorder |
|  |  | Eu60000 | 418201000006111 | [X]Paranoid personality disorder |
|  |  | Eu60713 | 418531000006117 | [X]Passive personality disorder |
|  |  | Eu60y15 | 418541000006110 | [X]Passive-aggressive personality disorder |
|  |  | Eu60412 | 424161000006118 | [X]Psychoinfantile personality disorder |
|  |  | Eu60y16 | 424201000006112 | [X]Psychoneurotic personality disorder |
|  |  | Eu60214 | 424211000006110 | [X]Psychopathic personality disorder |
|  |  | Eu60013 | 424331000006112 | [X]Querulant personality disorder |
|  |  | Eu60100 | 425591000006118 | [X]Schizoid personality disorder |
|  |  | Eu60714 | 425841000006112 | [X]Self defeating personality disorder |
|  |  | Eu60014 | 425911000006118 | [X]Sensitive paranoid personality disorder |
|  |  | Eu60215 | 427091000006119 | [X]Sociopathic personality disorder |
|  |  | E213.11 | 474121000006111 | Aggressive character |
|  |  | E217.11 | 480071000006112 | Amoral personality |
|  |  | E214.11 | 481471000006112 | Anancastic personality |
|  |  | E214000 | 481481000006110 | Anankastic personality |
|  |  | E217.00 | 487991000006113 | Antisocial or sociopathic personality disorder |
|  |  | E216.11 | 496091000006112 | Asthenic personality |
|  |  | E214.00 | 577931000006110 | Compulsive personality disorder |
|  |  | E211300 | 605901000006114 | Cyclothymic personality disorder |
|  |  | E211200 | 613781000006118 | Depressive personality disorder |
|  |  | E215200 | 640481000006113 | Emotionally unstable personality |
|  |  | E21y711 | 675901000006118 | Neurotic personality |
|  |  | E21..11 | 675911000006115 | Neurotic personality disorder |
|  |  | E216.13 | 735811000006119 | Labile personality |
|  |  | E212100 | 746351000006118 | Introverted personality |
|  |  | E216.00 | 782871000006113 | Inadequate personality disorder |
|  |  | E215.11 | 787571000006113 | Hysterical personality disorders |
|  |  | E215.00 | 824751000006111 | Histrionic personality disorder |
|  |  | E211299 | 882421000006113 | Depressive personality |
|  |  | E215.99 | 882431000006111 | Hysterical personality |
|  |  | E216.99 | 882441000006118 | Inadequate,passive personality |
|  |  | ^ESCTAN252038 | 2520381000006114 | Anancastic personality disorder |
|  |  | ^ESCTHY339857 | 3398571000006119 | Hysterical personality disorder |
|  |  | ^ESCTOB748840 | 7488401000006113 | Obsessional personality disorder |
| Psychoses |  | 1BH0.00 | 1452014 | Delusion of persecution |
|  |  | 1BH..11 | 4569010 | Delusion |
|  |  | E100500 | 9225016 | Schizophrenia in remission |
|  |  | E13y100 | 10122017 | Brief reactive psychosis |
|  |  | E100100 | 28758018 | Subchronic schizophrenia |
|  |  | 28D..00 | 30144016 | Confabulation |
|  |  | E106.00 | 43595011 | Residual schizophrenia |
|  |  | E122.00 | 44335019 | Paraphrenia |
|  |  | E103200 | 52897013 | Chronic paranoid schizophrenia |
|  |  | E102100 | 71539017 | Subchronic catatonic schizophrenia |
|  |  | E103500 | 105029017 | Paranoid schizophrenia in remission |
|  |  | E103.00 | 107878010 | Paranoid schizophrenia |
|  |  | E103100 | 132503015 | Subchronic paranoid schizophrenia |
|  |  | E135.00 | 138421012 | Agitated depression |
|  |  | E102500 | 178723016 | Catatonic schizophrenia in remission |
|  |  | 287..00 | 254847011 | O/E - bizarre appearance |
|  |  | 665C.00 | 264614015 | Inject.phenothiaz.stopped |
|  |  | E1...00 | 294724012 | Non-organic psychoses |
|  |  | E10..00 | 294725013 | Schizophrenic disorders |
|  |  | E100.11 | 294726014 | Schizophrenia simplex |
|  |  | E100.00 | 294727017 | Simple schizophrenia |
|  |  | E100000 | 294728010 | Unspecified schizophrenia |
|  |  | E100300 | 294730012 | Acute exacerbation of subchronic schizophrenia |
|  |  | E100400 | 294731011 | Acute exacerbation of chronic schizophrenia |
|  |  | E100z00 | 294734015 | Simple schizophrenia NOS |
|  |  | E101000 | 294735019 | Unspecified hebephrenic schizophrenia |
|  |  | E101400 | 294739013 | Acute exacerbation of chronic hebephrenic schizophrenia |
|  |  | E101500 | 294740010 | Hebephrenic schizophrenia in remission |
|  |  | E101z00 | 294741014 | Hebephrenic schizophrenia NOS |
|  |  | E102.00 | 294742019 | Catatonic schizophrenia |
|  |  | Eu20214 | 294743012 | [X]Schizophrenic flexibilatis cerea |
|  |  | E102000 | 294744018 | Unspecified catatonic schizophrenia |
|  |  | E102400 | 294751010 | Acute exacerbation of chronic catatonic schizophrenia |
|  |  | E102z00 | 294753013 | Catatonic schizophrenia NOS |
|  |  | E103000 | 294754019 | Unspecified paranoid schizophrenia |
|  |  | E103300 | 294757014 | Acute exacerbation of subchronic paranoid schizophrenia |
|  |  | E103400 | 294758016 | Acute exacerbation of chronic paranoid schizophrenia |
|  |  | E103z00 | 294760019 | Paranoid schizophrenia NOS |
|  |  | E105.00 | 294764011 | Latent schizophrenia |
|  |  | E105000 | 294765012 | Unspecified latent schizophrenia |
|  |  | E105200 | 294767016 | Chronic latent schizophrenia |
|  |  | E105500 | 294770017 | Latent schizophrenia in remission |
|  |  | E105z00 | 294771018 | Latent schizophrenia NOS |
|  |  | E107.11 | 294773015 | Cyclic schizophrenia |
|  |  | E10y100 | 294787015 | Coenesthopathic schizophrenia |
|  |  | E10y.11 | 294788013 | Cenesthopathic schizophrenia |
|  |  | E10yz00 | 294789017 | Other schizophrenia NOS |
|  |  | E10z.00 | 294790014 | Schizophrenia |
|  |  | E120.00 | 294904016 | Simple paranoid state |
|  |  | E123.11 | 294908018 | Folie a deux |
|  |  | E123.00 | 294909014 | Shared paranoid disorder |
|  |  | E12y.00 | 294910016 | Other paranoid states |
|  |  | E12y000 | 294911017 | Paranoia querulans |
|  |  | E12yz00 | 294912012 | Other paranoid states NOS |
|  |  | E12z.00 | 294913019 | Paranoid psychosis |
|  |  | E130.00 | 294917018 | Reactive depressive psychosis |
|  |  | E130.11 | 294918011 | Psychotic reactive depression |
|  |  | E131.00 | 294919015 | Acute hysterical psychosis |
|  |  | E132.00 | 294920014 | Reactive confusion |
|  |  | E134.00 | 294924017 | Psychogenic paranoid psychosis |
|  |  | E13y.00 | 294926015 | Other reactive psychoses |
|  |  | E13y000 | 294927012 | Psychogenic stupor |
|  |  | E13yz00 | 294929010 | Other reactive psychoses NOS |
|  |  | E14..00 | 294933015 | Psychoses with origin in childhood |
|  |  | E14y100 | 294944011 | Borderline psychosis of childhood |
|  |  | E14yz00 | 294945012 | Other childhood psychoses NOS |
|  |  | E1y..00 | 294949018 | Other specified non-organic psychoses |
|  |  | Eu20300 | 296031017 | Undifferentiated schizophrenia |
|  |  | Eu20z00 | 296040018 | [X]Schizophrenia, unspecified |
|  |  | Eu22z00 | 296066015 | [X]Persistent delusional disorder, unspecified |
|  |  | Eu23y00 | 296083012 | [X]Other acute and transient psychotic disorders |
|  |  | Eu25y00 | 296095012 | [X]Other schizoaffective disorders |
|  |  | Eu25z00 | 296096013 | [X]Schizoaffective disorder, unspecified |
|  |  | E13z.11 | 346895011 | Psychotic disorder |
|  |  | E13..11 | 346896012 | Reactive psychoses |
|  |  | E14z.11 | 346955013 | Childhood schizophrenia NOS |
|  |  | 1BH1.00 | 369805011 | Grandiose delusions |
|  |  | 6656 | 401521017 | On injectable phenothiazine |
|  |  | E104.00 | 401763015 | Acute schizophrenic episode |
|  |  | E10y.00 | 401764014 | Other schizophrenia |
|  |  | E121.00 | 401768012 | Chronic paranoid psychosis |
|  |  | E133.00 | 401770015 | Acute paranoid reaction |
|  |  | E1z..00 | 401771016 | Non-organic psychosis NOS |
|  |  | E14z.00 | 401772011 | Child psychosis NOS |
|  |  | Eu20y00 | 401855010 | [X]Other schizophrenia |
|  |  | Eu21.00 | 401856011 | Schizotypal personality disorder |
|  |  | Eu22y00 | 401857019 | [X]Other persistent delusional disorders |
|  |  | Eu23300 | 401859016 | [X]Other acute predominantly delusional psychotic disorders |
|  |  | Eu23z00 | 401860014 | [X]Acute and transient psychotic disorder, unspecified |
|  |  | Eu2z.00 | 401862018 | [X]Unspecified nonorganic psychosis |
|  |  | 285..00 | 402503018 | Neurotic condition, insight present |
|  |  | 285..11 | 402504012 | Psychotic condition, insight present |
|  |  | 286..00 | 402505013 | Poor insight into neurotic condition |
|  |  | 1BH..00 | 480834016 | Delusions |
|  |  | 1BH2.00 | 492910018 | Ideas of reference |
|  |  | 1Ba1.00 | 493041013 | Disorder of form of thought |
|  |  | 1B1E.00 | 501492014 | Hallucinations |
|  |  | E10y000 | 1219653018 | Atypical schizophrenia |
|  |  | E100200 | 1234861017 | Chronic schizophrenic |
|  |  | 665D.00 | 2474658013 | Neuroleptic medication due |
|  |  | 665E.00 | 2474659017 | Injectable neuroleptic given |
|  |  | 665F.00 | 2474661014 | On injectable neuroleptic |
|  |  | 1BH3.00 | 2548181016 | Paranoid ideation |
|  |  | 1B1b.00 | 2549087014 | Transient hallucinations |
|  |  | E13..00 | 25461000006115 | Other nonorganic psychoses |
|  |  | E107000 | 78531000006116 | Unspecified schizo-affective schizophrenia |
|  |  | E107100 | 123611000006110 | Subchronic schizo-affective schizophrenia |
|  |  | E107.00 | 155141000006116 | Schizoaffective schizophrenia |
|  |  | E107500 | 155151000006119 | Schizoaffective schizophrenia in remission |
|  |  | E107z00 | 155161000006117 | Schizo-affective schizophrenia NOS |
|  |  | E121.11 | 161371000006118 | Sander's disease |
|  |  | E106.11 | 169061000006112 | Restzustand - schizophrenia |
|  |  | 286..11 | 215691000006112 | Poor insight into psychotic condition |
|  |  | Eu22000 | 215841000000114 | Delusional disorder |
|  |  | Eu22011 | 215851000000112 | [X]Paranoid psychosis |
|  |  | Eu22015 | 215871000000115 | [X]Paranoia |
|  |  | 6656.11 | 218971000000116 | Phenothiazine injection - monitor |
|  |  | E11..13 | 223601000000119 | Manic psychosis |
|  |  | E13z.00 | 223611000000117 | Non-organic psychosis |
|  |  | E12..00 | 243361000006117 | Paranoid disorder |
|  |  | Eu23.00 | 362271000006110 | Acute transient psychotic disorder |
|  |  | Eu23100 | 362381000006115 | Acute polymorphic psychotic disorder co-occurrent with symptoms of schizophrenia |
|  |  | Eu23000 | 362391000006117 | Acute polymorphic psychotic disorder without symptoms of schizophrenia |
|  |  | Eu23200 | 362421000006113 | Acute schizophrenia-like psychotic disorder |
|  |  | Eu20311 | 366571000006114 | [X]Atypical schizophrenia |
|  |  | Eu21.12 | 367951000006113 | Borderline schizophrenia |
|  |  | Eu23011 | 367961000006110 | [X]Bouffee delirante |
|  |  | Eu23z11 | 367991000006119 | [X]Brief reactive psychosis NOS |
|  |  | Eu23211 | 368001000006112 | [X]Brief schizophreniform disorder |
|  |  | Eu23212 | 368011000006110 | [X]Brief schizophrenifrm psych |
|  |  | Eu20200 | 370451000006110 | [X]Catatonic schizophrenia |
|  |  | Eu20211 | 370461000006112 | [X]Catatonic stupor |
|  |  | Eu2y.11 | 370981000006112 | [X]Chronic hallucinatory psychosis |
|  |  | Eu20511 | 371031000006115 | [X]Chronic undifferentiated schizophrenia |
|  |  | Eu25211 | 376251000006112 | [X]Cyclic schizophrenia |
|  |  | Eu23012 | 376271000006119 | [X]Cycloid psychosis |
|  |  | Eu23112 | 376281000006116 | Cycloid psychosis |
|  |  | Eu22y11 | 376501000006110 | [X]Delusional dysmorphophobia |
|  |  | Eu20111 | 378051000006119 | [X]Disorganised schizophrenia |
|  |  | Eu24.11 | 387071000006117 | [X]Folie a deux |
|  |  | Eu20100 | 388741000006110 | [X]Hebephrenic schizophrenia |
|  |  | Eu24.00 | 389641000006110 | Induced delusional disorder |
|  |  | Eu24.12 | 389651000006112 | [X]Induced paranoid disorder |
|  |  | Eu24.13 | 389661000006114 | [X]Induced psychotic disorder |
|  |  | Eu22y12 | 394461000006110 | [X]Involutional paranoid state |
|  |  | Eu21.13 | 395021000006111 | [X]Latent schizophrenia |
|  |  | Eu21.11 | 395031000006114 | [X]Latent schizophrenic reaction |
|  |  | Eu25212 | 398631000006113 | Mixed schizophrenic and affective pschosis |
|  |  | Eu2y.00 | 412201000006113 | [X]Other nonorganic psychotic disorders |
|  |  | Eu22y13 | 418181000006110 | [X]Paranoia querulans |
|  |  | Eu20000 | 418221000006118 | [X]Paranoid schizophrenia |
|  |  | Eu22012 | 418231000006115 | [X]Paranoid state |
|  |  | Eu22013 | 418251000006110 | Late paraphrenia |
|  |  | Eu20011 | 418261000006112 | [X]Paraphrenic schizophrenia |
|  |  | Eu22.00 | 419861000006117 | Persistent delusional disorder |
|  |  | Eu20400 | 423041000006119 | [X]Post-schizophrenic depression |
|  |  | Eu21.14 | 423271000006116 | Prepsychotic schizophrenia |
|  |  | Eu21.15 | 423471000006117 | Prodromal schizophrenia |
|  |  | Eu21.16 | 423731000006114 | [X]Pseudoneurotic schizophrenia |
|  |  | Eu21.17 | 423741000006116 | [X]Pseudopsychopathic schizophrenia |
|  |  | Eu23312 | 424071000006112 | [X]Psychogenic paranoid psychosis |
|  |  | Eu2z.11 | 424231000006116 | Psychotic |
|  |  | Eu23z12 | 424511000006112 | [X]Reactive psychosis |
|  |  | Eu20500 | 424841000006113 | [X]Residual schizophrenia |
|  |  | Eu25100 | 425501000006113 | Schizoaffective disorder, depressive type |
|  |  | Eu25000 | 425511000006111 | Schizoaffective disorder, manic type |
|  |  | Eu25200 | 425521000006115 | Schizoaffective disorder, mixed type |
|  |  | Eu25.00 | 425541000006110 | Schizoaffective disorder |
|  |  | Eu25z11 | 425551000006112 | [X]Schizoaffective psychosis NOS |
|  |  | Eu25111 | 425561000006114 | [X]Schizoaffective psychosis, depressive type |
|  |  | Eu25011 | 425571000006119 | [X]Schizoaffective psychosis, manic type |
|  |  | Eu20.00 | 425601000006114 | [X]Schizophrenia |
|  |  | Eu20212 | 425641000006111 | [X]Schizophrenic catalepsy |
|  |  | Eu20213 | 425651000006113 | [X]Schizophrenic catatonia |
|  |  | Eu23214 | 425671000006115 | [X]Schizophrenic reaction |
|  |  | Eu20y12 | 425681000006117 | [X]Schizophreniform disord NOS |
|  |  | Eu25112 | 425691000006119 | [X]Schizophreniform psychosis, depressive type |
|  |  | Eu25012 | 425701000006119 | [X]Schizophreniform psychosis, manic type |
|  |  | Eu20y13 | 425711000006116 | [X]Schizophrenifrm psychos NOS |
|  |  | Eu21.18 | 425731000006110 | [X]Schizotypal personality disorder |
|  |  | Eu22014 | 425921000006114 | [X]Sensitiver Beziehungswahn |
|  |  | Eu20600 | 426881000006111 | [X]Simple schizophrenia |
|  |  | E107400 | 456731000006115 | Acute exacerbation of chronic schizo-affective schizophrenia |
|  |  | E107300 | 456801000006115 | Acute exacerbation subchronic schizo-affective schizophrenia |
|  |  | E11..00 | 473201000006114 | Mood disorder |
|  |  | 212W.00 | 492821000000113 | Schizophrenia resolved |
|  |  | 212X.00 | 492881000000114 | Psychosis resolved |
|  |  | E11..11 | 513871000006118 | Bipolar disorder |
|  |  | E133.11 | 523481000006114 | Bouffee delirante |
|  |  | E107200 | 556631000006116 | Chronic schizoaffective schizophrenia |
|  |  | E11..12 | 613791000006115 | Depressive psychoses |
|  |  | 665H.00 | 693361000000119 | Injectable neuroleptic managed by practice |
|  |  | E101.00 | 819351000006115 | Hebephrenic schizophrenia |
|  |  | E107.99 | 882301000006112 | Acute schizo affective psychosis |
|  |  | E11..99 | 882311000006110 | Manic-depressive psychoses |
|  |  | Eu22111 | 914461000006118 | [X]Capgras syndrome |
|  |  | Eu22100 | 914471000006113 | [X]Delusional misidentification syndrome |
|  |  | Eu22200 | 967791000006111 | [X]Cotard syndrome |
|  |  | 212T.00 | 996171000006114 | Psychosis, schizophrenia and bipolar affective disorder resolved |
|  |  | Eu22300 | 1667581000000114 | [X]Paranoid state in remission |
|  |  | Eu26.00 | 1667591000000111 | [X]Nonorganic psychosis in remission |
|  |  | 665G.00 | 1679341000006117 | Injectable neuroleptic managed by community mental health team |
|  |  | ^ESCTDI306812 | 3068121000006112 | Disorganised schizophrenia |
|  |  | ^ESCTCH386266 | 3862661000006112 | Chronic schizophrenia |
| Psycogenic disorders |  | E201800 | 7046013 | Hysterical fugue |
|  |  | E207.00 | 30729012 | Hypochondriasis |
|  |  | E201B00 | 56251019 | Compensation neurosis |
|  |  | E201612 | 73437013 | Globus hystericus |
|  |  | E260100 | 108724018 | Psychogenic torticollis |
|  |  | E201200 | 117876016 | Hysterical deafness |
|  |  | E201100 | 147404016 | Hysterical blindness |
|  |  | E201400 | 147537017 | Hysterical paralysis |
|  |  | E261500 | 158091017 | Psychogenic aphonia |
|  |  | E264311 | 196858012 | Spurious diarrhoea |
|  |  | E201000 | 294964018 | Hysteria unspecified |
|  |  | E201300 | 294965017 | Hysterical tremor |
|  |  | E201500 | 294968015 | Hysterical seizures |
|  |  | E201A00 | 294978017 | Dissociative reaction unspecified |
|  |  | E20y.00 | 295026012 | Other neurotic disorders |
|  |  | E20y100 | 295029017 | Writer's cramp neurosis |
|  |  | E20y200 | 295031014 | Other occupational neurosis |
|  |  | E20y300 | 295032019 | Psychasthenic neurosis |
|  |  | E260.00 | 295342013 | Psychogenic musculoskeletal symptoms |
|  |  | E260z00 | 295346011 | Psychogenic musculoskeletal symptoms NOS |
|  |  | E261000 | 295348012 | Psychogenic air hunger |
|  |  | E261100 | 295349016 | Psychogenic cough |
|  |  | E261200 | 295350016 | Psychogenic hiccough |
|  |  | E261300 | 295351017 | Psychogenic hyperventilation |
|  |  | E261400 | 295355014 | Psychogenic yawning |
|  |  | E261z00 | 295357018 | Psychogenic respiratory symptom |
|  |  | E262200 | 295360013 | Neurocirculatory asthenia |
|  |  | E262z00 | 295362017 | Psychogenic cardiovascular symptom |
|  |  | E263.00 | 295363010 | Psychogenic skin symptoms |
|  |  | E263000 | 295364016 | Psychogenic pruritus |
|  |  | E263z00 | 295366019 | Psychogenic skin symptoms NOS |
|  |  | E264200 | 295374018 | Cyclical vomiting - psychogenic |
|  |  | E264400 | 295381013 | Psychogenic dyspepsia |
|  |  | E264500 | 295382018 | Psychogenic constipation |
|  |  | E264z00 | 295383011 | Psychogenic gastrointestinal tract symptom NOS |
|  |  | E265.00 | 295384017 | Psychogenic genitourinary tract symptoms |
|  |  | E265200 | 295387012 | Psychogenic dysmenorrhea |
|  |  | E265300 | 295388019 | Psychogenic dysuria |
|  |  | E265z00 | 295389010 | Psychogenic genitourinary tract symptom NOS |
|  |  | E267.00 | 295391019 | Psychogenic symptom of special sense organ |
|  |  | E26y.00 | 295392014 | Other psychogenic malfunction |
|  |  | E26yz00 | 295394010 | Other psychogenic malfunction NOS |
|  |  | E26z.00 | 295395011 | Psychosomatic disorder NOS |
|  |  | E27..00 | 295396012 | Psychogenic syndromes NEC |
|  |  | E264.11 | 346997019 | Globus abdominalis |
|  |  | E201600 | 401778010 | Conversion disorder |
|  |  | E201z00 | 401779019 | Hysteria NOS |
|  |  | E264300 | 401804015 | Psychogenic diarrhoea |
|  |  | E264011 | 484680014 | Air swallowing - excessive |
|  |  | E264000 | 484681013 | Psychogenic aerophagy |
|  |  | E201.00 | 493878012 | Hysteria |
|  |  | E206.00 | 1216885017 | Depersonalisation syndrome |
|  |  | E20y000 | 1774451016 | Somatization disorder |
|  |  | E20y011 | 1785963019 | Briquet's disorder |
|  |  | E262000 | 2162219012 | Cardiac neurosis |
|  |  | E201C00 | 2648186013 | Phantom pregnancy |
|  |  | E262300 | 199321000006118 | Psychogenic cardiovascular disorder |
|  |  | E262.00 | 199341000006113 | Psychogenic cardiovascular symptoms |
|  |  | E264.00 | 199441000006117 | Somatoform autonomic dysfunction - gastrointestinal tract |
|  |  | E260000 | 199531000006114 | Psychogenic paralysis |
|  |  | E261.00 | 199581000006110 | Psychogenic respiratory symptoms |
|  |  | E265100 | 199671000006111 | Psychologic vaginismus |
|  |  | E201z12 | 223621000000111 | Ataxia - hysterical |
|  |  | E26..00 | 232501000006118 | Physiological malfunction arising from mental factors |
|  |  | E201z11 | 489141000006110 | Aphonia - hysterical |
|  |  | E201611 | 496061000006116 | Astasia - abasia, hysterical |
|  |  | E201900 | 694811000006118 | Multiple personality |
|  |  | E201511 | 760791000006116 | Fit - hysterical |
|  |  | E201700 | 787521000006112 | Hysterical amnesia |
|  |  | E201z13 | 798571000006111 | Ganser's syndrome - hysterical |
|  |  | E261.99 | 882541000006117 | Pschogenic resp. symptoms |
|  |  | E262099 | 882551000006115 | Cardiac neuroses |
|  |  | E265.99 | 882561000006118 | Psychogenic GU symptoms |
|  |  | E26z.99 | 882571000006113 | Other psychogenic symptoms |
| Self harm |  | 1BD1.00 | 11734010 | Suicidal ideation |
|  |  | 1BD..00 | 253125016 | Harmful thoughts |
|  |  | 1BD2.00 | 253126015 | Morbid thoughts |
|  |  | TK01000 | 331522012 | Suicide and self inflicted injury by Amylobarbitone |
|  |  | TK01100 | 331523019 | Suicide and self inflicted injury by Barbitone |
|  |  | TK01200 | 331524013 | Suicide and self inflicted injury by Butabarbitone |
|  |  | TK01300 | 331525014 | Suicide and self inflicted injury by Pentabarbitone |
|  |  | TK01400 | 331526010 | Suicide and self inflicted injury by Phenobarbitone |
|  |  | TK01z00 | 331528011 | Suicide and self inflicted injury by barbiturates |
|  |  | TK1y.00 | 331542015 | Suicide and selfinflicted poisoning by other utility gas |
|  |  | TK21.00 | 331546017 | Suicide and selfinflicted poisoning by other carbon monoxide |
|  |  | TK4..00 | 331555019 | Suicide and selfinflicted injury by drowning |
|  |  | TK54.00 | 331561016 | Suicide and selfinflicted injury by other firearm |
|  |  | TK60100 | 331566014 | Self inflicted lacerations to wrist |
|  |  | TKx..00 | 331574010 | Suicide and selfinflicted injury by other means |
|  |  | TKxy.00 | 331586013 | Suicide and selfinflicted injury by other specified means |
|  |  | TKxz.00 | 331587016 | Suicide and selfinflicted injury by other means NOS |
|  |  | TKz..00 | 331589018 | Suicide and selfinflicted injury NOS |
|  |  | U200.12 | 334616010 | Ibuprofen overdose |
|  |  | U202.15 | 334642010 | Nitrazepam overdose |
|  |  | U202.11 | 334643017 | Sedative overdose |
|  |  | U202.13 | 334644011 | Overdose of temazepam |
|  |  | U202.14 | 334645012 | Flurazepam overdose |
|  |  | U204.13 | 334668015 | Selective serotonin re-uptake inhibitor overdose |
|  |  | U204.11 | 334669011 | Overdose of antidepressant drug |
|  |  | U204.12 | 334670012 | Amitriptyline overdose |
|  |  | U20A.11 | 334743014 | [X]Self poisoning from glue solvent |
|  |  | U22..00 | 334805018 | [X]Intentional self harm by drowning and submersion |
|  |  | U23..00 | 334817016 | [X]Intentional self harm by handgun discharge |
|  |  | U26..00 | 334853019 | [X]Intentional self harm by explosive material |
|  |  | U27..00 | 334864016 | [X]Intentional self harm by smoke, fire and flames |
|  |  | U29..00 | 334886015 | [X]Intentional self harm by sharp object |
|  |  | U2A..00 | 334897015 | [X]Intentional self harm by blunt object |
|  |  | U2B..00 | 334908015 | Attempted suicide - jumping from a high place |
|  |  | U2D..00 | 334931015 | [X]Intentional self harm by crashing of motor vehicle |
|  |  | U2E..00 | 334942012 | Self-mutilation |
|  |  | U2y..00 | 334943019 | Intentionally harming self |
|  |  | U2z..00 | 334956014 | [X]Intentional self harm by unspecified means |
|  |  | 1BD3.00 | 369784017 | Suicidal plans |
|  |  | 1B19.00 | 397957012 | Suicidal |
|  |  | TK...00 | 404265013 | Suicide and selfinflicted injury |
|  |  | TK...14 | 404266014 | Suicide and self harm |
|  |  | U2...00 | 404287016 | Self-injurious behaviour |
|  |  | U20C.11 | 410813016 | [X]Self poisoning with weedkiller |
|  |  | TK...12 | 413175018 | Injury - self-inflicted |
|  |  | 1BD5.00 | 1488407019 | High suicide risk |
|  |  | 1BD6.00 | 1488408012 | Moderate suicide risk |
|  |  | 1BD7.00 | 1488409016 | Low suicide risk |
|  |  | 1BDA.00 | 1780423014 | Thoughts of deliberate self harm |
|  |  | 1BDB.00 | 1780424015 | Plans for deliberate self harm without intent |
|  |  | 1BDC.00 | 1780425019 | Intent of deliberate self harm with detailed plans |
|  |  | 1BDD.00 | 1780426018 | Unknown risk of deliberate self harm |
|  |  | 1BD8.00 | 1787477010 | At risk of DSH - deliberate self harm |
|  |  | TK01411 | 2475831012 | Suicide and self inflicted injury by Phenobarbital |
|  |  | 1BD4.00 | 2535917013 | Suicide risk |
|  |  | 1B19.11 | 119471000006114 | Suicidal - symptom |
|  |  | TK3z.00 | 119501000006119 | Suicide + selfinflicted inj by hang/strangle/suffocate NOS |
|  |  | TK3y.00 | 119511000006116 | Suicide + selfinflicted inj oth mean hang/strangle/suffocate |
|  |  | TKx0z00 | 119521000006112 | Suicide + selfinflicted inj-jump/lie before moving obj NOS |
|  |  | TK3..00 | 119531000006110 | Suicide + selfinflicted injury by hang/strangulate/suffocate |
|  |  | TK31.00 | 119541000006117 | Suicide + selfinflicted injury by suffocation by plastic bag |
|  |  | TKx0.00 | 119551000006115 | Suicide + selfinflicted injury-jump/lie before moving object |
|  |  | TKx0000 | 119561000006118 | Suicide + selfinflicted injury-jumping before moving object |
|  |  | TKx0100 | 119571000006113 | Suicide + selfinflicted injury-lying before moving object |
|  |  | TK06.00 | 119581000006111 | Suicide + selfinflicted poisoning by agricultural chemical |
|  |  | TK00.00 | 119591000006114 | Suicide + selfinflicted poisoning by analgesic/antipyretic |
|  |  | TK08.00 | 119601000006118 | Suicide + selfinflicted poisoning by arsenic + its compounds |
|  |  | TK01.00 | 119611000006115 | Suicide + selfinflicted poisoning by barbiturates |
|  |  | TK07.00 | 119621000006111 | Suicide + selfinflicted poisoning by corrosive/caustic subst |
|  |  | TK1z.00 | 119631000006114 | Suicide + selfinflicted poisoning by domestic gases NOS |
|  |  | TK05.00 | 119641000006116 | Intentional self poisoning |
|  |  | TK10.00 | 119651000006119 | Suicide and selfinflicted poisoning by gas distributed by pipeline |
|  |  | TK2z.00 | 119661000006117 | Suicide + selfinflicted poisoning by gases and vapours NOS |
|  |  | TK1..00 | 119671000006112 | Suicide + selfinflicted poisoning by gases in domestic use |
|  |  | TK11.00 | 119681000006110 | Suicide and selfinflicted poisoning by liquefied petroleum gas distributed in mobile containers |
|  |  | TK20.00 | 119691000006113 | Suicide + selfinflicted poisoning by motor veh exhaust gas |
|  |  | TK02.00 | 119701000006113 | Suicide + selfinflicted poisoning by oth sedatives/hypnotics |
|  |  | TK04.00 | 119711000006111 | Intentional poisoning by drug |
|  |  | TK2..00 | 119721000006115 | Suicide + selfinflicted poisoning by other gases and vapours |
|  |  | TK2y.00 | 119731000006117 | Suicide + selfinflicted poisoning by other gases and vapours |
|  |  | TK0..00 | 119751000006112 | Self poisoning by non-drug solid or liquid agents |
|  |  | TK03.00 | 119761000006114 | Suicide + selfinflicted poisoning tranquilliser/psychotropic |
|  |  | TKx1.00 | 119861000006116 | Suicide and selfinflicted injury by burns or fire |
|  |  | TKx5.00 | 119871000006111 | Suicide and selfinflicted injury by crashing motor vehicle |
|  |  | TK60.00 | 119891000006112 | Suicide and selfinflicted injury by cutting |
|  |  | TK6..00 | 119901000006111 | Suicide and selfinflicted injury by cutting and stabbing |
|  |  | TK6z.00 | 119911000006114 | Suicide and selfinflicted injury by cutting and stabbing NOS |
|  |  | TKx4.00 | 119931000006115 | Suicide and selfinflicted injury by electrocution |
|  |  | TK55.00 | 119941000006113 | Suicide and selfinflicted injury by explosives |
|  |  | TKx3.00 | 119951000006110 | Suicide and selfinflicted injury by extremes of cold |
|  |  | TK5..00 | 119961000006112 | Suicide and selfinflicted injury by firearms and explosives |
|  |  | TK5z.00 | 119971000006117 | Suicide and selfinflicted injury by firearms/explosives NOS |
|  |  | TK50.00 | 119981000006119 | Suicide and selfinflicted injury by handgun |
|  |  | TK30.00 | 119991000006116 | Attempted suicide - hanging |
|  |  | TK7..00 | 120011000006112 | Suicide and selfinflicted injury by jumping from high place |
|  |  | TKx2.00 | 120071000006115 | Suicide and selfinflicted injury by scald |
|  |  | TK51.00 | 120081000006117 | Suicide and selfinflicted injury by shotgun |
|  |  | TK61.00 | 120091000006119 | Suicide and selfinflicted injury by stabbing |
|  |  | TKx7.00 | 120101000006113 | Suicide and selfinflicted injury caustic subst, excl poison |
|  |  | TK7z.00 | 120161000006114 | Suicide+selfinflicted injury-jump from high place NOS |
|  |  | TK72.00 | 120171000006119 | Suicide+selfinflicted injury-jump from natural sites |
|  |  | TK71.00 | 120181000006116 | Suicide+selfinflicted injury-jump from oth manmade structure |
|  |  | TK70.00 | 120191000006118 | Suicide+selfinflicted injury-jump from residential premises |
|  |  | TK60111 | 136541000006115 | Slashed wrists self inflicted |
|  |  | TK...13 | 220641000006117 | Deliberate self poisoning |
|  |  | U20C.00 | 222791000000110 | [X]Intentional self poisoning by and exposure to pesticides |
|  |  | TK...17 | 250291000006113 | Parasuicide |
|  |  | TK01011 | 345851000000113 | Suicide and self inflicted injury by amobarbital |
|  |  | U2...14 | 366461000006115 | [X]Attempted suicide |
|  |  | U20..11 | 376401000006114 | [X]Deliberate drug overdose / other poisoning |
|  |  | U2...12 | 390531000006111 | [X]Injury - self-inflicted |
|  |  | U2B6.00 | 391121000006110 | [X]Intentional self harm by jumping from a high place, occurrence at industrial and construction area |
|  |  | U2By.00 | 391131000006113 | [X]Intentional self harm by jumping from a high place, occurrence at other specified place |
|  |  | U2Bz.00 | 391141000006115 | [X]Intentional self harm by jumping from a high place, occurrence at unspecified place |
|  |  | U282.00 | 391151000006118 | [X]Intentional self harm by steam, hot vapours and hot objects, occurrence at school, other institution and public administrative area |
|  |  | U286.00 | 391161000006116 | [X]Intentional self harm by steam, hot vapours and hot objects, occurrence at industrial and construction area |
|  |  | U283.00 | 391171000006111 | [X]Intentional self harm by steam, hot vapours and hot objects, occurrence at sports and athletics area |
|  |  | U285.00 | 391181000006114 | [X]Intentional self harm by steam, hot vapours and hot objects, occurrence at trade and service area |
|  |  | U2D2.00 | 391191000006112 | [X]Intentional self harm by crashing of motor vehicle, occurrence at school, other institution and public administrative area |
|  |  | U2B2.00 | 391201000006110 | [X]Intentional self harm by jumping from a high place, occurrence at school, other institution and public administrative area |
|  |  | U2C3.00 | 391211000006113 | [X]Intentional self harm by jumping or lying before moving object, occurrence at sports and athletics area |
|  |  | U2C5.00 | 391221000006117 | [X]Intentional self harm by jumping or lying before moving object, occurrence at trade and service area |
|  |  | U2C2.00 | 391231000006119 | [X]Intentional self harm by jumping or lying before moving object, occurrence at school, other institution and public administrative area |
|  |  | U2Cy.00 | 391241000006112 | [X]Intentional self harm by jumping or lying before moving object, occurrence at other specified place |
|  |  | U2Cz.00 | 391251000006114 | [X]Intentional self harm by jumping or lying before moving object, occurrence at unspecified place |
|  |  | U2C6.00 | 391261000006111 | [X]Intentional self harm by jumping or lying before moving object, occurrence at industrial and construction area |
|  |  | U2C1.00 | 391271000006116 | [X]Intentional self harm by jumping or lying before moving object, occurrence in residential institution |
|  |  | U2C4.00 | 391281000006118 | [X]Intentional self harm by jumping or lying before moving object, occurrence on street and highway |
|  |  | U241.00 | 391291000006115 | [X]Intentional self harm by rifle, shotgun and larger firearm discharge, occurrence in residential institution |
|  |  | U244.00 | 391301000006119 | [X]Intentional self harm by rifle, shotgun and larger firearm discharge, occurrence on street and highway |
|  |  | U24z.00 | 391311000006116 | [X]Intentional self harm by rifle, shotgun and larger firearm discharge, occurrence at unspecified place |
|  |  | U24y.00 | 391321000006112 | [X]Intentional self harm by rifle, shotgun and larger firearm discharge, occurrence at other specified place |
|  |  | U243.00 | 391331000006110 | [X]Intentional self harm by rifle, shotgun and larger firearm discharge, occurrence at sports and athletics area |
|  |  | U245.00 | 391341000006117 | [X]Intentional self harm by rifle, shotgun and larger firearm discharge, occurrence at trade and service area |
|  |  | U209600 | 391351000006115 | [X]Intentional self poisoning by and exposure to alcohol, occurrence at industrial and construction area |
|  |  | U203600 | 391361000006118 | [X]Intentional self poisoning by and exposure to antiparkinson drugs, occurrence at industrial and construction area |
|  |  | U206600 | 391371000006113 | [X]Intentional self poisoning by and exposure to hallucinogens, occurrence at industrial and construction area |
|  |  | U205600 | 391381000006111 | [X]Intentional self poisoning by and exposure to narcotic drugs, occurrence at industrial and construction area |
|  |  | U200600 | 391391000006114 | [X]Intentional self poisoning by and exposure to nonopioid analgesics, occurrence at industrial and construction area |
|  |  | U20Az00 | 391401000006111 | [X]Intentional self poisoning by and exposure to organic solvents and halogenated hydrocarbons /vapours, halogens, occurrence at unspecified place |
|  |  | U20Ay00 | 391411000006114 | [X]Intentional self poisoning by and exposure to organic solvents and halogenated hydrocarbons /vapours, occurrence at other specified place |
|  |  | U20A600 | 391421000006118 | [X]Intentional self poisoning by and exposure to organic solvents and halogenated hydrocarbons /vapours, occurrence at industrial and construction area |
|  |  | U207600 | 391431000006115 | [X]Intentional self poisoning by and exposure to other autonomic drugs, occurrence at industrial and construction area |
|  |  | U208600 | 391441000006113 | [X]Int self pois oth/unsp drug/medic indust/construct area |
|  |  | U20B600 | 391451000006110 | [X]Int self pois other gas/vapour indust/construct area |
|  |  | U20C600 | 391461000006112 | [X]Intentional self poisoning by and exposure to pesticides, occurrence at industrial and construction area |
|  |  | U204600 | 391471000006117 | [X]Intentional self poisoning by and exposure to psychotropic drugs, occurrence at industrial and construction area |
|  |  | U202600 | 391481000006119 | [X]Intentional self poisoning by and exposure to sedative hypnotics, occurrence at industrial and construction area |
|  |  | U20y600 | 391491000006116 | [X]Int self pois unspecif chemical indust/construct area |
|  |  | U209300 | 391501000006112 | [X]Intentional self poisoning by and exposure to alcohol, occurrence at sports and athletics area |
|  |  | U209y00 | 391511000006110 | [X]Intentional self poisoning by and exposure to alcohol, occurrence at other specified place |
|  |  | U209200 | 391521000006119 | [X]Intentional self poisoning by and exposure to alcohol, occurrence at school, other institution and public administrative area |
|  |  | U201300 | 391531000006116 | [X]Int self poison antiepileptic in sport/athletic area |
|  |  | U201600 | 391541000006114 | [X]Intentional self poisoning by and exposure to antiepileptics, occurrence at industrial and construction area |
|  |  | U203y00 | 391551000006111 | [X]Intentional self poisoning by and exposure to antiparkinson drugs, occurrence at other specified place |
|  |  | U203300 | 391561000006113 | [X]Intentional self poisoning by and exposure to antiparkinson drugs, occurrence at sports and athletics area |
|  |  | U203200 | 391571000006118 | [X]Intentional self poisoning by and exposure to antiparkinson drugs, occurrence at school, other institution and public administrative area |
|  |  | U206y00 | 391581000006115 | [X]Intentional self poisoning by and exposure to hallucinogens, occurrence at other specified place |
|  |  | U206300 | 391591000006117 | [X]Intentional self poisoning by and exposure to hallucinogens, occurrence at sports and athletics area |
|  |  | U206200 | 391601000006113 | [X]Intentional self poisoning by and exposure to hallucinogens, occurrence at school, other institution and public administrative area |
|  |  | U205300 | 391611000006111 | [X]Intentional self poisoning by and exposure to narcotic drugs, occurrence at sports and athletics area |
|  |  | U205y00 | 391621000006115 | [X]Intentional self poisoning by and exposure to narcotic drugs, occurrence at other specified place |
|  |  | U205200 | 391631000006117 | [X]Intentional self poisoning by and exposure to narcotic drugs, occurrence at school, other institution and public administrative area |
|  |  | U200300 | 391641000006110 | [X]Intentional self poisoning by and exposure to nonopioid analgesics, occurrence at sports and athletics area |
|  |  | U200200 | 391651000006112 | [X]Intentional self poisoning by and exposure to nonopioid analgesics, occurrence at school, other institution and public administrative area |
|  |  | U200y00 | 391661000006114 | [X]Intentional self poisoning by and exposure to nonopioid analgesics, occurrence at other specified place |
|  |  | U20A200 | 391671000006119 | [X]Intentional self poisoning by and exposure to organic solvents and halogenated hydrocarbons /vapours, at school, other institution and pub admin area |
|  |  | U20A400 | 391681000006116 | [X]Intentional self poisoning by and exposure to organic solvents and halogenated hydrocarbons /vapours, on street and highway |
|  |  | U20A700 | 391691000006118 | [X]Intentional self poisoning by and exposure to organic solvents and halogenated hydrocarbons /vapours, on farm |
|  |  | U20A100 | 391701000006118 | [X]Intentional self poisoning by and exposure to organic solvents and halogenated hydrocarbons /vapours, in residential institution |
|  |  | U20A300 | 391711000006115 | [X]Intentional self poisoning by and exposure to organic solvents and halogenated hydrocarbons /vapours, halogens, at sports and athletics area |
|  |  | U20A500 | 391721000006111 | [X]Intentional self poisoning by and exposure to organic solvents and halogenated hydrocarbons /vapours, at trade and service area |
|  |  | U207300 | 391731000006114 | [X]Intentional self poisoning by and exposure to other autonomic drugs, at sports and athletics area |
|  |  | U207200 | 391741000006116 | [X]Intentional self poisoning by and exposure to other autonomic drugs, at school, other institution and public administrative area |
|  |  | U207y00 | 391751000006119 | [X]Intentional self poisoning by and exposure to other autonomic drugs, at other specified place |
|  |  | U208300 | 391761000006117 | [X]Int self poison oth/uns drug/med in sport/athletic area |
|  |  | U208200 | 391771000006112 | [X]Int self poison oth/uns drug/med school/pub admin area |
|  |  | U208y00 | 391781000006110 | [X]Int self poison oth/unsp drug/medic other spec place |
|  |  | U20B300 | 391791000006113 | [X]Int self poison other gas/vapour in sport/athletic area |
|  |  | U20By00 | 391801000006114 | [X]Int self poison other gas/vapour other spec place |
|  |  | U20B200 | 391811000006112 | [X]Int self poison other gas/vapour school/pub admin area |
|  |  | U20C300 | 391821000006116 | [X]Intentional self poisoning by and exposure to pesticides, occurrence at sports and athletics area |
|  |  | U20Cy00 | 391831000006118 | [X]Intentional self poisoning by and exposure to pesticides, occurrence at other specified place |
|  |  | U20C200 | 391841000006111 | [X]Intentional self poisoning by and exposure to pesticides, occurrence at school, other institution and public administrative area |
|  |  | U204300 | 391851000006113 | [X]Intentional self poisoning by and exposure to psychotropic drugs, occurrence at sports and athletics area |
|  |  | U204y00 | 391861000006110 | [X]Intentional self poisoning by and exposure to psychotropic drugs, occurrence at other specified place |
|  |  | U204200 | 391871000006115 | [X]Intentional self poisoning by and exposure to psychotropic drugs, occurrence at school, other institution and public administrative area |
|  |  | U202300 | 391881000006117 | [X]Intentional self poisoning by and exposure to sedative hypnotics, occurrence at sports and athletics area |
|  |  | U202y00 | 391891000006119 | [X]Intentional self poisoning by and exposure to sedative hypnotics, occurrence at other specified place |
|  |  | U202200 | 391901000006115 | [X]Intentional self poisoning by and exposure to sedative hypnotics, occurrence at school, other institution and public administrative area |
|  |  | U20y300 | 391911000006117 | [X]Int self poison unspecif chemical in sport/athletic area |
|  |  | U20yy00 | 391921000006113 | [X]Int self poison unspecif chemical other spec place |
|  |  | U20y200 | 391931000006111 | [X]Int self poison unspecif chemical school/pub admin area |
|  |  | U209000 | 391941000006118 | [X]Intentional self poisoning by and exposure to alcohol, occurrence at home |
|  |  | U209700 | 391951000006116 | [X]Intentional self poisoning by and exposure to alcohol, occurrence on farm |
|  |  | U201000 | 391961000006119 | [X]Intentional self poisoning by and exposure to antiepileptics, occurrence at home |
|  |  | U201700 | 391971000006114 | [X]Intentional self poisoning by and exposure to antiepileptics, occurrence on farm |
|  |  | U203000 | 391981000006112 | [X]Intentional self poisoning by and exposure to antiparkinson drugs, occurrence at home |
|  |  | U203700 | 391991000006110 | [X]Intentional self poisoning by and exposure to antiparkinson drugs, occurrence on farm |
|  |  | U206000 | 392001000006112 | [X]Intentional self poisoning by and exposure to hallucinogens, occurrence at home |
|  |  | U206700 | 392011000006110 | [X]Intentional self poisoning by and exposure to hallucinogens, occurrence on farm |
|  |  | U205000 | 392021000006119 | [X]Intentional self poisoning by and exposure to narcotic drugs, occurrence at home |
|  |  | U205700 | 392031000006116 | [X]Intentional self poisoning by and exposure to narcotic drugs, occurrence on farm |
|  |  | U200000 | 392041000006114 | Self-administered intentional poisoning by analgesic |
|  |  | U200700 | 392051000006111 | [X]Intentional self poisoning by and exposure to nonopioid analgesics, occurrence on farm |
|  |  | U207000 | 392061000006113 | [X]Intentional self poisoning by and exposure to other autonomic drugs, occurrence at home |
|  |  | U207700 | 392071000006118 | [X]Intentional self poisoning by and exposure to other autonomic drugs, occurrence on farm |
|  |  | U208700 | 392081000006115 | [X]Int self poison/exposure to oth/unsp drug/medic on farm |
|  |  | U208000 | 392091000006117 | [X]Int self poison/exposure to oth/unsp drug/medicam home |
|  |  | U20B000 | 392101000006111 | [X]Int self poison/exposure to other gas/vapour at home |
|  |  | U20B700 | 392111000006114 | [X]Int self poison/exposure to other gas/vapour on farm |
|  |  | U208.00 | 392121000006118 | [X]Int self poison/exposure to other/unspec drug/medicament |
|  |  | U20C000 | 392131000006115 | [X]Intentional self poisoning by and exposure to pesticides, occurrence at home |
|  |  | U20C700 | 392141000006113 | [X]Intentional self poisoning by and exposure to pesticides, occurrence on farm |
|  |  | U204000 | 392151000006110 | [X]Intentional self poisoning by and exposure to psychotropic drugs, occurrence at home |
|  |  | U204700 | 392161000006112 | [X]Intentional self poisoning by and exposure to psychotropic drugs, occurrence on farm |
|  |  | U202000 | 392171000006117 | [X]Intentional self poisoning by and exposure to sedative hypnotics, occurrence at home |
|  |  | U202700 | 392181000006119 | [X]Intentional self poisoning by and exposure to sedative hypnotics, occurrence on farm |
|  |  | U20y000 | 392191000006116 | [X]Int self poison/exposure to unspecif chemical at home |
|  |  | U20y700 | 392201000006118 | [X]Int self poison/exposure to unspecif chemical on farm |
|  |  | U242.00 | 392211000006115 | [X]Intentional self harm by rifle, shotgun and larger firearm discharge, occurrence at school, other institution and public administrative area |
|  |  | U246.00 | 392221000006111 | [X]Intentional self harm by rifle, shotgun and larger firearm discharge, occurrence at industrial and construction area |
|  |  | U252.00 | 392231000006114 | [X]Intentional self harm by other and unspecified firearm discharge, occurrence at school, other institution and public administrative area |
|  |  | U256.00 | 392241000006116 | [X]Intentional self harm by other and unspecified firearm discharge, occurrence at industrial and construction area |
|  |  | U253.00 | 392251000006119 | [X]Intentional self harm by other and unspecified firearm discharge, occurrence at sports and athletics area |
|  |  | U212.00 | 392261000006117 | [X]Inten slf harm hang strang/suffc sch oth ins/pub adm area |
|  |  | U2A2.00 | 392281000006110 | [X]Intentional self harm by blunt object, occurrence at school, other institution and public administrative area |
|  |  | U2A6.00 | 392291000006113 | [X]Intentional self harm by blunt object, occurrence at industrial and construction area |
|  |  | U2A1.00 | 392301000006114 | [X]Intentional self harm by blunt object, occurrence in residential institution |
|  |  | U2A3.00 | 392311000006112 | [X]Intentional self harm by blunt object, occurrence at sports and athletics area |
|  |  | U2A5.00 | 392321000006116 | [X]Intentional self harm by blunt object, occurrence at trade and service area |
|  |  | U2Dy.00 | 392331000006118 | [X]Intentional self harm by crashing of motor vehicle, occurrence at other specified place |
|  |  | U2Dz.00 | 392341000006111 | [X]Intentional self harm by crashing of motor vehicle, occurrence at unspecified place |
|  |  | U2D1.00 | 392351000006113 | [X]Intentional self harm by crashing of motor vehicle, occurrence in residential institution |
|  |  | U2D3.00 | 392361000006110 | [X]Intentional self harm by crashing of motor vehicle, occurrence at sports and athletics area |
|  |  | U2D4.00 | 392371000006115 | [X]Intentional self harm by crashing of motor vehicle, occurrence on street and highway |
|  |  | U2D0.00 | 392381000006117 | [X]Intentional self harm by crashing of motor vehicle, occurrence at home |
|  |  | U2D7.00 | 392391000006119 | [X]Intentional self harm by crashing of motor vehicle, occurrence on farm |
|  |  | U226.00 | 392401000006117 | [X]Intentional self harm by drowning and submersion, occurrence at industrial and construction area |
|  |  | U22y.00 | 392411000006119 | [X]Intentional self harm by drowning and submersion, occurrence at other specified place |
|  |  | U223.00 | 392421000006110 | [X]Intentional self harm by drowning and submersion, occurrence at sports and athletics area |
|  |  | U225.00 | 392431000006113 | [X]Intentional self harm by drowning and submersion, occurrence at trade and service area |
|  |  | U22z.00 | 392441000006115 | [X]Intentional self harm by drowning and submersion, occurrence at unspecified place |
|  |  | U220.00 | 392451000006118 | [X]Intentional self harm by drowning and submersion, occurrence at home |
|  |  | U227.00 | 392461000006116 | [X]Intentional self harm by drowning and submersion, occurrence on farm |
|  |  | U221.00 | 392471000006111 | [X]Intentional self harm by drowning and submersion, occurrence in residential institution |
|  |  | U224.00 | 392481000006114 | [X]Intentional self harm by drowning and submersion, occurrence on street and highway |
|  |  | U26y.00 | 392491000006112 | [X]Intentional self harm by explosive material, occurrence at other specified place |
|  |  | U26z.00 | 392501000006116 | [X]Intentional self harm by explosive material, occurrence at unspecified place |
|  |  | U262.00 | 392511000006118 | [X]Intentional self harm by explosive material, occurrence at school, other institution and public administrative area |
|  |  | U266.00 | 392521000006114 | [X]Intentional self harm by explosive material, occurrence at industrial and construction area |
|  |  | U263.00 | 392531000006112 | [X]Intentional self harm by explosive material, occurrence at sports and athletics area |
|  |  | U265.00 | 392541000006119 | [X]Intentional self harm by explosive material, occurrence at trade and service area |
|  |  | U23y.00 | 392551000006117 | [X]Intentional self harm by handgun discharge, occurrence at other specified place |
|  |  | U23z.00 | 392561000006115 | [X]Intentional self harm by handgun discharge, occurrence at unspecified place |
|  |  | U231.00 | 392571000006110 | [X]Intentional self harm by handgun discharge, occurrence in residential institution |
|  |  | U236.00 | 392581000006113 | [X]Intentional self harm by handgun discharge, occurrence at industrial and construction area |
|  |  | U234.00 | 392591000006111 | [X]Intentional self harm by handgun discharge, occurrence on street and highway |
|  |  | U233.00 | 392601000006115 | [X]Intentional self harm by handgun discharge, occurrence at sports and athletics area |
|  |  | U235.00 | 392611000006117 | [X]Intentional self harm by handgun discharge, occurrence at trade and service area |
|  |  | U216.00 | 392621000006113 | [X]Intent self harm by hang strangl/suffc indust/constr area |
|  |  | U213.00 | 392631000006111 | [X]Intent self harm by hang strangl/suffc sport/athlet area |
|  |  | U215.00 | 392641000006118 | [X]Intent self harm by hang strangl/suffc trade/service area |
|  |  | U217.00 | 392651000006116 | [X]Intent self harm by hanging strangulat/suffocat occ farm |
|  |  | U210.00 | 392661000006119 | [X]Intent self harm by hanging strangulat/suffocat occ home |
|  |  | U21..00 | 392671000006114 | Self-destructive behavior |
|  |  | U21y.00 | 392681000006112 | [X]Intent self harm by hangng strangul/suffoct oth spec plce |
|  |  | U21z.00 | 392691000006110 | [X]Intent self harm by hangng strangul/suffoct unspecif plce |
|  |  | U211.00 | 392701000006110 | [X]Intent self harm by hangng strangult/suffoct resid instit |
|  |  | U214.00 | 392711000006113 | [X]Intent self harm by hangng strangult/suffoct street/h'way |
|  |  | U2B1.00 | 392721000006117 | [X]Intentional self harm by jumping from a high place, occurrence in residential institution |
|  |  | U2B4.00 | 392731000006119 | [X]Intentional self harm by jumping from a high place, occurrence on street and highway |
|  |  | U2B3.00 | 392741000006112 | [X]Intentional self harm by jumping from a high place, occurrence at sports and athletics area |
|  |  | U2B5.00 | 392751000006114 | [X]Intentional self harm by jumping from a high place, occurrence at trade and service area |
|  |  | U2C7.00 | 392761000006111 | [X]Intentional self harm by jumping or lying before moving object, occurrence on farm |
|  |  | U2C0.00 | 392771000006116 | [X]Intentional self harm by jumping or lying before moving object, occurrence at home |
|  |  | U2C..00 | 392781000006118 | [X]Intentional self harm by jumping or lying before moving object |
|  |  | U2B0.00 | 392791000006115 | [X]Intentional self harm by jumping from a high place, occurrence at home |
|  |  | U2B7.00 | 392801000006119 | [X]Intentional self harm by jumping from a high place, occurrence on farm |
|  |  | U2y1.00 | 392811000006116 | [X]Intentional self harm by other specified means, occurrence in residential institution |
|  |  | U2y3.00 | 392821000006112 | [X]Intentional self harm by other specified means, occurrence at sports and athletics area |
|  |  | U2y4.00 | 392831000006110 | [X]Intentional self harm by other specified means, occurrence on street and highway |
|  |  | U2y5.00 | 392841000006117 | [X]Intentional self harm by other specified means, occurrence at trade and service area |
|  |  | U2yz.00 | 392851000006115 | [X]Intentional self harm by other specified means, occurrence at unspecified place |
|  |  | U25..00 | 392861000006118 | [X]Intentional self harm by other and unspecified firearm discharge |
|  |  | U24..00 | 392871000006113 | [X]Intentional self harm by rifle, shotgun and larger firearm discharge |
|  |  | U296.00 | 392881000006111 | [X]Intentional self harm by sharp object, occurrence at industrial and construction area |
|  |  | U291.00 | 392891000006114 | [X]Intentional self harm by sharp object, occurrence in residential institution |
|  |  | U293.00 | 392901000006113 | [X]Intentional self harm by sharp object, occurrence at sports and athletics area |
|  |  | U295.00 | 392911000006111 | [X]Intentional self harm by sharp object, occurrence at trade and service area |
|  |  | U276.00 | 392921000006115 | [X]Intentional self harm by smoke, fire and flames, occurrence at industrial and construction area |
|  |  | U273.00 | 392931000006117 | [X]Intentional self harm by smoke, fire and flames, occurrence at sports and athletics area |
|  |  | U275.00 | 392941000006110 | [X]Intentional self harm by smoke, fire and flames, occurrence at trade and service area |
|  |  | U27y.00 | 392951000006112 | [X]Intentional self harm by smoke, fire and flames, occurrence at other specified place |
|  |  | U271.00 | 392961000006114 | [X]Intentional self harm by smoke, fire and flames, occurrence in residential institution |
|  |  | U274.00 | 392971000006119 | [X]Intentional self harm by smoke, fire and flames, occurrence on street and highway |
|  |  | U272.00 | 392981000006116 | [X]Intentional self harm by smoke, fire and flames, occurrence at school, other institution and public administrative area |
|  |  | U27z.00 | 392991000006118 | [X]Intentional self harm by smoke, fire and flames, occurrence at unspecified place |
|  |  | U280.00 | 393001000006117 | [X]Intentional self harm by steam, hot vapours and hot objects, occurrence at home |
|  |  | U287.00 | 393011000006119 | [X]Intentional self harm by steam, hot vapours and hot objects, occurrence on farm |
|  |  | U281.00 | 393021000006110 | [X]Intentional self harm by steam, hot vapours and hot objects, occurrence in residential institution |
|  |  | U284.00 | 393031000006113 | [X]Intentional self harm by steam, hot vapours and hot objects, occurrence on street and highway |
|  |  | U28z.00 | 393041000006115 | [X]Intentional self harm by steam, hot vapours and hot objects, occurrence at unspecified place |
|  |  | U28y.00 | 393051000006118 | [X]Intentional self harm by steam, hot vapours and hot objects, occurrence at other specified place |
|  |  | U2z2.00 | 393061000006116 | [X]Intentional self harm by unspecified means, occurrence at school, other institution and public administrative area |
|  |  | U2zz.00 | 393071000006111 | [X]Intentional self harm by unspecified means, occurrence at unspecified place |
|  |  | U2zy.00 | 393081000006114 | [X]Intentional self harm by unspecified means, occurrence at other specified place |
|  |  | U2z1.00 | 393091000006112 | [X]Intentional self harm by unspecified means, occurrence in residential institution |
|  |  | U2z4.00 | 393101000006118 | [X]Intentional self harm by unspecified means, occurrence on street and highway |
|  |  | U2D6.00 | 393111000006115 | [X]Intentional self harm by crashing of motor vehicle, occurrence at industrial and construction area |
|  |  | U2D5.00 | 393121000006111 | [X]Intentional self harm by crashing of motor vehicle, occurrence at trade and service area |
|  |  | U222.00 | 393131000006114 | [X]Intentional self harm by drowning and submersion, occurrence at school, other institution and public administrative area |
|  |  | U232.00 | 393141000006116 | [X]Intentional self harm by handgun discharge, occurrence at school, other institution and public administrative area |
|  |  | U2y2.00 | 393151000006119 | [X]Intentional self harm by other specified means, occurrence at school, other institution and public administrative area |
|  |  | U2y6.00 | 393161000006117 | [X]Intentional self harm by other specified means, occurrence at industrial and construction area |
|  |  | U2yy.00 | 393171000006112 | [X]Intentional self harm by other specified means, occurrence at other specified place |
|  |  | U255.00 | 393181000006110 | [X]Intentional self harm by other and unspecified firearm discharge, occurrence at trade and service area |
|  |  | U251.00 | 393191000006113 | [X]Intentional self harm by other and unspecified firearm discharge, occurrence in residential institution |
|  |  | U254.00 | 393201000006111 | [X]Intentional self harm by other and unspecified firearm discharge, occurrence on street and highway |
|  |  | U25z.00 | 393211000006114 | [X]Intentional self harm by other and unspecified firearm discharge, occurrence at unspecified place |
|  |  | U25y.00 | 393221000006118 | [X]Intentional self harm by other and unspecified firearm discharge, occurrence at other specified place |
|  |  | U250.00 | 393231000006115 | [X]Intentional self harm by other and unspecified firearm discharge, occurrence at home |
|  |  | U257.00 | 393241000006113 | [X]Intentional self harm by other and unspecified firearm discharge, occurrence on farm |
|  |  | U247.00 | 393251000006110 | [X]Intentional self harm by rifle, shotgun and larger firearm discharge, occurrence on farm |
|  |  | U240.00 | 393261000006112 | [X]Intentional self harm by rifle, shotgun and larger firearm discharge, occurrence at home |
|  |  | U292.00 | 393271000006117 | [X]Intentional self harm by sharp object, occurrence at school, other institution and public administrative area |
|  |  | U2z6.00 | 393281000006119 | [X]Intentional self harm by unspecified means, occurrence at industrial and construction area |
|  |  | U2z3.00 | 393291000006116 | [X]Intentional self harm by unspecified means, occurrence at sports and athletics area |
|  |  | U2z5.00 | 393301000006115 | [X]Intentional self harm by unspecified means, occurrence at trade and service area |
|  |  | U209400 | 393311000006117 | [X]Intentional self poisoning by and exposure to alcohol, occurrence on street and highway |
|  |  | U209500 | 393321000006113 | [X]Intentional self poisoning by and exposure to alcohol, occurrence at trade and service area |
|  |  | U201400 | 393331000006111 | [X]Intentional self poisoning by and exposure to antiepileptics, occurrence on street and highway |
|  |  | U201500 | 393341000006118 | [X]Intentional self poisoning by and exposure to antiepileptics, occurrence at trade and service area |
|  |  | U203400 | 393351000006116 | [X]Intentional self poisoning by and exposure to antiparkinson drugs, occurrence on street and highway |
|  |  | U203500 | 393361000006119 | [X]Intentional self poisoning by and exposure to antiparkinson drugs, occurrence at trade and service area |
|  |  | U206400 | 393371000006114 | [X]Intentional self poisoning by and exposure to hallucinogens, occurrence on street and highway |
|  |  | U206500 | 393381000006112 | [X]Intentional self poisoning by and exposure to hallucinogens, occurrence at trade and service area |
|  |  | U205400 | 393391000006110 | [X]Intentional self poisoning by and exposure to narcotic drugs, occurrence on street and highway |
|  |  | U205500 | 393401000006112 | [X]Intentional self poisoning by and exposure to narcotic drugs, occurrence at trade and service area |
|  |  | U201200 | 393411000006110 | [X]Intentional self poisoning by and exposure to antiepileptics, occurrence at school, other institution and public administrative area |
|  |  | U200400 | 393421000006119 | [X]Intentional self poisoning by and exposure to nonopioid analgesics, occurrence on street and highway |
|  |  | U200500 | 393431000006116 | [X]Intentional self poisoning by and exposure to nonopioid analgesics, occurrence at trade and service area |
|  |  | U20A000 | 393441000006114 | [X]Intentional self poisoning by and exposure to organic solvents and halogenated hydrocarbons /vapours, occurrence at home |
|  |  | U207400 | 393451000006111 | [X]Intentional self poisoning by and exposure to other autonomic drugs, occurrence on street and highway |
|  |  | U207500 | 393461000006113 | [X]Intentional self poisoning by and exposure to other autonomic drugs, occurrence at trade and service area |
|  |  | U208400 | 393471000006118 | [X]Intent self pois oth/unsp drug/medic in street/highway |
|  |  | U208500 | 393481000006115 | [X]Intent self pois oth/unsp drug/medic trade/service area |
|  |  | U20B400 | 393491000006117 | [X]Intent self pois other gas/vapour in street/highway |
|  |  | U20B500 | 393501000006113 | [X]Intent self pois other gas/vapour trade/service area |
|  |  | U20C400 | 393511000006111 | [X]Intentional self poisoning by and exposure to pesticides, occurrence on street and highway |
|  |  | U20C500 | 393521000006115 | [X]Intentional self poisoning by and exposure to pesticides, occurrence at trade and service area |
|  |  | U204400 | 393531000006117 | [X]Intentional self poisoning by and exposure to psychotropic drugs, occurrence on street and highway |
|  |  | U204500 | 393541000006110 | [X]Intentional self poisoning by and exposure to psychotropic drugs, occurrence at trade and service area |
|  |  | U202400 | 393551000006112 | [X]Intentional self poisoning by and exposure to sedative hypnotics, occurrence on street and highway |
|  |  | U202500 | 393561000006114 | [X]Intentional self poisoning by and exposure to sedative hypnotics, occurrence at trade and service area |
|  |  | U20y400 | 393571000006119 | [X]Intent self pois unspecif chemical in street/highway |
|  |  | U20y500 | 393581000006116 | [X]Intent self pois unspecif chemical trade/service area |
|  |  | U209100 | 393591000006118 | [X]Intentional self poisoning by and exposure to alcohol, occurrence in residential institution |
|  |  | U209z00 | 393601000006114 | [X]Intentional self poisoning by and exposure to alcohol, occurrence at unspecified place |
|  |  | U201100 | 393611000006112 | [X]Intentional self poisoning by and exposure to antiepileptics, occurrence in residential institution |
|  |  | U201y00 | 393621000006116 | [X]Intentional self poisoning by and exposure to antiepileptics, occurrence at other specified place |
|  |  | U201z00 | 393631000006118 | [X]Intentional self poisoning by and exposure to antiepileptics, occurrence at unspecified place |
|  |  | U203100 | 393641000006111 | [X]Intentional self poisoning by and exposure to antiparkinson drugs, occurrence in residential institution |
|  |  | U203z00 | 393651000006113 | [X]Intentional self poisoning by and exposure to antiparkinson drugs, occurrence at unspecified place |
|  |  | U206100 | 393661000006110 | [X]Intentional self poisoning by and exposure to hallucinogens, occurrence in residential institution |
|  |  | U206z00 | 393671000006115 | [X]Intentional self poisoning by and exposure to hallucinogens, occurrence at unspecified place |
|  |  | U205100 | 393681000006117 | [X]Intentional self poisoning by and exposure to narcotic drugs, occurrence in residential institution |
|  |  | U205z00 | 393691000006119 | [X]Intentional self poisoning by and exposure to narcotic drugs, occurrence at unspecified place |
|  |  | U200100 | 393701000006119 | [X]Intentional self poisoning by and exposure to nonopioid analgesics, occurrence in residential institution |
|  |  | U200z00 | 393711000006116 | [X]Intentional self poisoning by and exposure to nonopioid analgesics, occurrence at unspecified place |
|  |  | U207100 | 393721000006112 | [X]Intentional self poisoning by and exposure to other autonomic drugs, occurrence in residential institution |
|  |  | U207z00 | 393731000006110 | [X]Intentional self poisoning by and exposure to other autonomic drugs, occurrence at unspecified place |
|  |  | U208z00 | 393741000006117 | [X]Intent self poison oth/unsp drug/medic unspecif place |
|  |  | U208100 | 393751000006115 | [X]Intent self poison oth/unsp drug/medicam res institut |
|  |  | U20B100 | 393761000006118 | [X]Intent self poison other gas/vapour at res institut |
|  |  | U20Bz00 | 393771000006113 | [X]Intent self poison other gas/vapour unspecif place |
|  |  | U20C100 | 393781000006111 | [X]Intentional self poisoning by and exposure to pesticides, occurrence in residential institution |
|  |  | U20Cz00 | 393791000006114 | [X]Intentional self poisoning by and exposure to pesticides, occurrence at unspecified place |
|  |  | U204100 | 393801000006110 | [X]Intentional self poisoning by and exposure to psychotropic drugs, occurrence in residential institution |
|  |  | U204z00 | 393811000006113 | [X]Intentional self poisoning by and exposure to psychotropic drugs, occurrence at unspecified place |
|  |  | U202100 | 393821000006117 | [X]Intentional self poisoning by and exposure to sedative hypnotics, occurrence in residential institution |
|  |  | U202z00 | 393831000006119 | [X]Intentional self poisoning by and exposure to sedative hypnotics, occurrence at unspecified place |
|  |  | U20y100 | 393841000006112 | [X]Intent self poison unspecif chemical at res institut |
|  |  | U20yz00 | 393851000006114 | [X]Intent self poison unspecif chemical unspecif place |
|  |  | U209.00 | 393861000006111 | [X]Intentional self poisoning by and exposure to alcohol |
|  |  | U201.00 | 393871000006116 | [X]Intentional self poisoning by and exposure to antiepileptics |
|  |  | U203.00 | 393881000006118 | [X]Intentional self poisoning by and exposure to antiparkinson drugs |
|  |  | U206.00 | 393891000006115 | [X]Intentional self poisoning by and exposure to hallucinogens |
|  |  | U205.00 | 393901000006116 | Intentional narcotic poisoning |
|  |  | U200.00 | 393911000006118 | [X]Intentional self poisoning by and exposure to nonopioid analgesics |
|  |  | U207.00 | 393921000006114 | [X]Intentional self poisoning by and exposure to other autonomic drugs |
|  |  | U20B.00 | 393931000006112 | [X]Intent self poison/exposure to other gas/vapour |
|  |  | U204.00 | 393951000006117 | [X]Intentional self poisoning by and exposure to psychotropic drugs |
|  |  | U202.00 | 393961000006115 | [X]Intentional self poisoning by and exposure to sedative hypnotics |
|  |  | U20y.00 | 393971000006110 | [X]Intent self poison/exposure to unspecif chemical |
|  |  | U2Ay.00 | 393981000006113 | [X]Intentional self harm by blunt object, occurrence at other specified place |
|  |  | U2A4.00 | 393991000006111 | [X]Intentional self harm by blunt object, occurrence on street and highway |
|  |  | U261.00 | 394001000006119 | [X]Intentional self harm by explosive material, occurrence in residential institution |
|  |  | U264.00 | 394011000006116 | [X]Intentional self harm by explosive material, occurrence on street and highway |
|  |  | U267.00 | 394021000006112 | [X]Intentional self harm by explosive material, occurrence on farm |
|  |  | U260.00 | 394031000006110 | [X]Intentional self harm by explosive material, occurrence at home |
|  |  | U230.00 | 394041000006117 | [X]Intentional self harm by handgun discharge, occurrence at home |
|  |  | U237.00 | 394051000006115 | [X]Intentional self harm by handgun discharge, occurrence on farm |
|  |  | U29y.00 | 394061000006118 | [X]Intentional self harm by sharp object, occurrence at other specified place |
|  |  | U294.00 | 394071000006113 | [X]Intentional self harm by sharp object, occurrence on street and highway |
|  |  | U270.00 | 394081000006111 | [X]Intentional self harm by smoke, fire and flames, occurrence at home |
|  |  | U277.00 | 394091000006114 | [X]Intentional self harm by smoke, fire and flames, occurrence on farm |
|  |  | U2Az.00 | 394111000006117 | [X]Intentional self harm by blunt object, occurrence at unspecified place |
|  |  | U2A0.00 | 394121000006113 | [X]Intentional self harm by blunt object, occurrence at home |
|  |  | U2A7.00 | 394131000006111 | [X]Intentional self harm by blunt object, occurrence on farm |
|  |  | U29z.00 | 394211000006111 | [X]Intentional self harm by sharp object, occurrence at unspecified place |
|  |  | U290.00 | 394221000006115 | [X]Intentional self harm by sharp object, occurrence at home |
|  |  | U297.00 | 394231000006117 | [X]Intentional self harm by sharp object, occurrence on farm |
|  |  | U28..00 | 394251000006112 | [X]Intentional self harm by steam, hot vapours and hot objects |
|  |  | U2z0.00 | 394261000006114 | [X]Intentional self harm by unspecified means, occurrence at home |
|  |  | U2z7.00 | 394271000006119 | [X]Intentional self harm by unspecified means, occurrence on farm |
|  |  | U20A.00 | 394291000006118 | [X]Intentional self poisoning by and exposure to organic solvents and halogenated hydrocarbons /vapours |
|  |  | U20..00 | 394301000006117 | [X]Intentional self poisoning by and exposure to noxious substances |
|  |  | U2y0.00 | 394341000006115 | [X]Intentional self harm by other specified means, occurrence at home |
|  |  | U2y7.00 | 394351000006118 | [X]Intentional self harm by other specified means, occurrence on farm |
|  |  | U202.18 | 417701000006118 | Amobarbital overdose |
|  |  | U200.13 | 417721000006111 | [X]Overdose - aspirin |
|  |  | U202.17 | 417731000006114 | Barbiturate overdose |
|  |  | U202.16 | 417741000006116 | Benzodiazepine overdose |
|  |  | U202.12 | 417751000006119 | Diazepam overdose |
|  |  | U205.11 | 417771000006112 | Heroin overdose |
|  |  | U200.11 | 417801000006114 | Paracetamol overdose |
|  |  | U2...15 | 418121000006111 | [X]Para-suicide |
|  |  | U20B.11 | 425831000006119 | [X]Self carbon monoxide poisoning |
|  |  | U2...11 | 425851000006114 | [X]Self inflicted injury |
|  |  | U20C.12 | 425881000006118 | [X]Self poisoning with paraquat |
|  |  | U2...13 | 428251000006115 | Suicide |
|  |  | TK...15 | 498561000006113 | Suicide attempt |
|  |  | TK...11 | 539941000006110 | Intentional drug overdose |
|  |  | TK1..99 | 902561000006110 | Attempt suicide - domestic gas |
|  |  | TK20.98 | 902571000006115 | Suicide - car exhaust |
|  |  | TK20.99 | 902581000006117 | Attempt suicide - car exhaust |
|  |  | TK30.98 | 902591000006119 | Suicide - hanging |
|  |  | TK30.99 | 902601000006110 | Attempt suicide - hanging |
|  |  | TK31.98 | 902611000006113 | Suicide - suffocate |
|  |  | TK31.99 | 902621000006117 | Attempt suicide - suffocate |
|  |  | TK4..98 | 902631000006119 | Suicide - drowning |
|  |  | TK4..99 | 902641000006112 | Attempt suicide - drowning |
|  |  | TK5..98 | 902651000006114 | Suicide - firearms |
|  |  | TK5..99 | 902661000006111 | Attempt suicide - firearms |
|  |  | TK6..98 | 902671000006116 | Suicide - cut/stab |
|  |  | TK6..99 | 902681000006118 | Attempt suicide - cut/stab |
|  |  | TK7..98 | 902691000006115 | Suicide - jump from high |
|  |  | TK7..99 | 902701000006115 | Attempt suicide-jump from high |
|  |  | TKz..98 | 902711000006117 | Suicide - NOS |
|  |  | TKz..99 | 902721000006113 | Attempt suicide - NOS |
|  |  | TK...98 | 991081000006119 | Attempt suicide - NOS |
|  |  | 1BDE.00 | 2151531000000111 | Suicide risk increased from previous level |
|  |  | 1BDF.00 | 2151571000000113 | Suicide risk unchanged from previous level |
| Stress events |  | 13Z2.00 | 7762013 | Educational problem |
|  |  | 13HM.00 | 37385015 | Legal problem |
|  |  | 13H4200 | 65545019 | Marital conflict |
|  |  | 13H7.00 | 97258010 | Unwanted pregnancy |
|  |  | 13H3.00 | 105113011 | Divorce |
|  |  | 13HL.13 | 120897018 | Cultural deprivation |
|  |  | 13HL.14 | 169463011 | Social withdrawal |
|  |  | 13HF.00 | 169470011 | Broken engagement |
|  |  | 13HB.00 | 169485017 | Divorced parents |
|  |  | 13HR.00 | 169602014 | On probation |
|  |  | 13H2.00 | 250640019 | Separation |
|  |  | 13H3100 | 250645012 | Divorce proceedings pending |
|  |  | 13H4100 | 250650018 | Marital breakdown |
|  |  | 13HA.00 | 250675019 | Battered wife - history |
|  |  | 13HC.00 | 250676018 | Battered husband - history |
|  |  | 13HD.00 | 250677010 | Violent spouse |
|  |  | 13HJ.00 | 250705011 | Father remarried |
|  |  | 13HK.00 | 250706012 | Mother remarried |
|  |  | 13HM000 | 250717013 | Charged with crime |
|  |  | 13HM100 | 250718015 | Compensation claim pending |
|  |  | 13HM200 | 250719011 | Legal insurance problem |
|  |  | 13HM300 | 250720017 | Litigation pending |
|  |  | 13HM400 | 250721018 | Court case pending |
|  |  | 13HM500 | 250722013 | Pleads not guilty |
|  |  | 13HM600 | 250723015 | Pleads guilty |
|  |  | 13HM700 | 250724014 | Lost custody of children |
|  |  | 13HP200 | 250735016 | Poor family relationship |
|  |  | 13HP300 | 250736015 | Parental marital problems |
|  |  | 13HP400 | 250737012 | Parent relationship problem |
|  |  | 13HP500 | 250738019 | Stepparent relationship problem |
|  |  | 13HP600 | 250739010 | Violence between parents |
|  |  | 13HP700 | 250740012 | Child relationship problem |
|  |  | 13HS.11 | 250745019 | Impending prosecution |
|  |  | 13HS.00 | 250746018 | On remand |
|  |  | 13HV.00 | 250764013 | Extra-marital problems |
|  |  | 13HV100 | 250769015 | Affair ended |
|  |  | 13HV200 | 250770019 | Affair unsatisfactory |
|  |  | 13HV400 | 250777016 | Seven year itch - marital |
|  |  | 13HVZ00 | 250778014 | Extra-marital problems NOS |
|  |  | 13Z2.11 | 251264017 | School problem |
|  |  | 13Z2.12 | 251265016 | School difficulties |
|  |  | 1468 | 251632016 | H/O: psychological trauma |
|  |  | 13HQ.00 | 291519013 | In prison |
|  |  | 13HQ.12 | 291520019 | Prison sentence |
|  |  | TL0z.00 | 331598015 | Homicide or assault by fight, brawl or rape NOS |
|  |  | U3K..00 | 335193014 | [X]Assault by bodily force |
|  |  | U3K0.00 | 335194015 | [X]Assault by bodily force, occurrence at home |
|  |  | U3K7.00 | 335202015 | [X]Assault by bodily force, occurrence on farm |
|  |  | U3Kz.00 | 335204019 | [X]Assault by bodily force, occurrence at unspecified place |
|  |  | U3L..12 | 335205018 | [X]Attempted rape |
|  |  | U3L..00 | 335206017 | Forcible sexual assault |
|  |  | U3L0.00 | 335207014 | [X]Sexual assault by bodily force, occurrence at home |
|  |  | U3M..00 | 335217016 | [X]Neglect and abandonment |
|  |  | U3M0.00 | 335218014 | [X]Neglect and abandonment, by spouse or partner |
|  |  | U3M1.00 | 335219018 | [X]Neglect and abandonment, by parent |
|  |  | U3M2.00 | 335220012 | [X]Neglect and abandonment, by acquaintance or friend |
|  |  | U3My.00 | 335221011 | [X]Neglect and abandonment, by other specified persons |
|  |  | U3Mz.00 | 335222016 | [X]Neglect and abandonment, by unspecified person |
|  |  | U3N..00 | 335224015 | [X]Other maltreatment syndromes |
|  |  | U3N0.00 | 335225019 | [X]Other maltreatment syndromes, by spouse or partner |
|  |  | U3N1.00 | 335226018 | [X]Other maltreatment syndromes, by parent |
|  |  | U3N2.00 | 335227010 | [X]Other maltreatment syndromes, by acquaintance or friend |
|  |  | U3N3.00 | 335228017 | [X]Other maltreatment syndromes, by official authorities |
|  |  | U3Ny.00 | 335229013 | [X]Other maltreatment syndromes, by other specified persons |
|  |  | U3Nz.00 | 335230015 | [X]Other maltreatment syndromes, by unspecified person |
|  |  | U3y..00 | 335231016 | [X]Assault by other specified means |
|  |  | U3y0.00 | 335232011 | [X]Assault by other specified means, occurrence at home |
|  |  | U3z..00 | 335242013 | [X]Assault by unspecified means |
|  |  | U3z0.00 | 335243015 | [X]Assault by unspecified means, occurrence at home |
|  |  | U3z7.00 | 335250016 | [X]Assault by unspecified means, occurrence on farm |
|  |  | 13HU.11 | 337135016 | Prisoner of war - Japan |
|  |  | 13HU.12 | 337136015 | Prisoner of war - Germany |
|  |  | 13H3000 | 338024013 | Divorce proceedings |
|  |  | 14X5.00 | 339324014 | Victim of physical abuse |
|  |  | 14X6.00 | 339326011 | Victim of sexual abuse |
|  |  | 13HN000 | 370285010 | Theft |
|  |  | 13HN100 | 370296019 | Shoplifting |
|  |  | TL01.11 | 370402013 | Sexual assault |
|  |  | 13H6.00 | 397763010 | Single parent |
|  |  | 13H9.00 | 397765015 | Imprisonment record |
|  |  | 13H9.11 | 397766019 | Prison record |
|  |  | 13HG.00 | 397767011 | Broken with partner |
|  |  | 13HH.00 | 397768018 | Looks after chronically sick relative |
|  |  | 13HI.00 | 397769014 | Parents separated |
|  |  | 13HN.11 | 397771014 | Police record |
|  |  | 13HN.00 | 397772019 | Criminal record |
|  |  | 13HT115 | 397773012 | Domestic problems |
|  |  | 13HT.00 | 397774018 | Home problems |
|  |  | 13HU.00 | 397776016 | Prisoner of war |
|  |  | 13HV000 | 397777013 | Affair started |
|  |  | TL01.00 | 404274010 | Homicide or assault by rape |
|  |  | 13HI.12 | 411073015 | Father left home |
|  |  | 13HI.13 | 411074014 | Mother left home |
|  |  | 13HH.11 | 412029016 | Cares for mentally handicapped dependent |
|  |  | 13HH.12 | 412030014 | Looks after chronically sick father |
|  |  | 13HH.13 | 412031013 | Looks after chronically sick husband |
|  |  | 13HH.14 | 412032018 | Looks after chronically sick mother |
|  |  | 13HH.15 | 412033011 | Looks after chronically sick spouse |
|  |  | 13HH.16 | 412034017 | Looks after chronically sick wife |
|  |  | 13HH.17 | 412035016 | Looks after elderly dependent |
|  |  | 13HH.18 | 412036015 | Looks after physically handicapped dependent |
|  |  | 13HN.12 | 412083018 | Vandalism record |
|  |  | 13HQ.11 | 412084012 | Arrested in police custody |
|  |  | 13HM.11 | 412086014 | Legal problem with separation |
|  |  | 13HM.12 | 412087017 | Legal problem with divorce |
|  |  | 13H3.11 | 412088010 | Divorce problems |
|  |  | 13H5.12 | 412089019 | Spouse returned home |
|  |  | 13HT.11 | 412093013 | Unhappy childhood |
|  |  | 13HT.12 | 412095018 | Unhappy home |
|  |  | 13HT113 | 412096017 | Home unsettled |
|  |  | 13HV011 | 412097014 | Lover taken |
|  |  | 13HV012 | 412098016 | Mistress taken |
|  |  | 13HV312 | 412099012 | Wife committed adultery |
|  |  | 13HV313 | 412100016 | Husband committed adultery |
|  |  | 13HV314 | 412101017 | Oil rig wives syndrome |
|  |  | 13H4213 | 412102012 | Row with wife |
|  |  | 13H4311 | 412103019 | Spouse unsympathetic |
|  |  | 13H4312 | 412104013 | Spouse inattentive |
|  |  | 13HG.11 | 412135019 | Spouse left home |
|  |  | 13H9.12 | 415855010 | Released from prison |
|  |  | 13HT112 | 443774017 | Tower block syndrome |
|  |  | 13HT114 | 443775016 | Wife unable to cope |
|  |  | ZV4H600 | 451481010 | [V]Lack of learning and play experience |
|  |  | ZV19C00 | 455516019 | [V]Family history of physical abuse to sibling |
|  |  | ZV19E00 | 455518018 | [V]Family history of sexual abuse to sibling |
|  |  | ZV19G00 | 455520015 | [V]Family history of mental abuse to sibling |
|  |  | ZV19J00 | 455522011 | [V]Family history of sibling abuse NOS |
|  |  | ZV19K00 | 455523018 | [V]Family history of sibling abuse by family member NOS |
|  |  | ZV4G700 | 455537010 | Bullying of child |
|  |  | 14X..00 | 457072016 | History of abuse |
|  |  | 14X0.00 | 457073014 | History of physical abuse |
|  |  | 14X1.00 | 457074015 | History of sexual abuse |
|  |  | 14X2.00 | 457075019 | History of emotional abuse |
|  |  | ZV4FA00 | 460796018 | Institutional upbringing |
|  |  | ZV4G.00 | 460797010 | [V]Problems related to negative life events in childhood |
|  |  | ZV4G100 | 460799013 | [V]Removal from home in childhood |
|  |  | ZV4G200 | 460800012 | [V]Altered pattern of family relationships in childhood |
|  |  | ZV4G300 | 460801011 | [V]Events resulting in loss of self-esteem in childhood |
|  |  | ZV4G500 | 460803014 | [V]Problems related to alleged physical abuse of child |
|  |  | ZV4G600 | 460804015 | [V]Personal frightening experience in childhood |
|  |  | ZV4Gy00 | 460806018 | [V]Other negative life events in childhood |
|  |  | ZV4H.00 | 460807010 | [V]Other problems related to upbringing |
|  |  | ZV4H300 | 460811016 | [V]Emotional neglect of child |
|  |  | ZV4H400 | 460812011 | [V]Other problems related to neglect in upbringing |
|  |  | ZV4Hy00 | 460815013 | [V]Other specified problems related to upbringing |
|  |  | ZV61200 | 460982018 | [V]Child abuse |
|  |  | 13H8.00 | 476066010 | Illegitimate pregnancy |
|  |  | 13HV311 | 483247015 | Spouse committed infidelity |
|  |  | 13HV300 | 483248013 | Spouse committed adultery |
|  |  | 13H4300 | 486188017 | Maladjustment to married life |
|  |  | 13H4.00 | 500032013 | Marital problems |
|  |  | 13HB.11 | 1219112010 | Parents divorced |
|  |  | ZV61212 | 1227742014 | [V]Child neglect |
|  |  | ZV61213 | 1227743016 | [V]Parent - child conflict |
|  |  | ZV61211 | 1227744010 | [V]Child battering |
|  |  | ZV61511 | 1227747015 | [V]Alcoholism in family |
|  |  | 14X3.00 | 2474290010 | History of domestic violence |
|  |  | 14X7.00 | 2576632018 | Victim of emotional abuse |
|  |  | 13HL.00 | 2645385015 | Social isolation |
|  |  | 13HL.11 | 2645386019 | Family isolation |
|  |  | 13HL.12 | 2645387011 | Social outcast |
|  |  | 14XD.00 | 2695471013 | History of domestic abuse |
|  |  | 13H7.11 | 72771000006114 | Unwanted child |
|  |  | 13H6.11 | 81121000006114 | Unmarried parent |
|  |  | 13HT100 | 121671000006117 | Stress at home |
|  |  | 14X8.00 | 314681000000113 | Victim of domestic violence |
|  |  | ZV19H00 | 339961000006111 | [V]Family history of mental abuse to sibling by family member |
|  |  | ZV19F00 | 339971000006116 | [V]Family history of sexual abuse to sibling by family member |
|  |  | ZV19D00 | 339981000006118 | [V]Family history of physical abuse to sibling by family member |
|  |  | ZV4F900 | 346711000006119 | [V]Problems related to alleged sexual of abuse child by person outside primary support group |
|  |  | U3K..11 | 358921000006113 | [X] Assault by fight |
|  |  | U3K5.00 | 364111000006115 | [X]Assault by bodily force occurrence at trade/service area |
|  |  | U3Ky.00 | 364131000006114 | [X]Assault by bodily force occurrn at other specified place |
|  |  | U3K1.00 | 364141000006116 | [X]Assault by bodily force occurrn in residential institut'n |
|  |  | U3K2.00 | 364151000006119 | [X]Assault by bodily force occurrn sch oth ins/pub adm area |
|  |  | U3K3.00 | 364171000006112 | [X]Assault by bodily force, occurrence at sport/athlet area |
|  |  | U3K4.00 | 364201000006111 | [X]Assault by bodily force, occurrence on street / highway |
|  |  | U3y2.00 | 365111000006119 | [X]Assault by oth specif means occ sch oth ins/pub adm area |
|  |  | U3y1.00 | 365141000006115 | [X]Assault by oth specif means occurrn resident institution |
|  |  | U3y3.00 | 365151000006118 | [X]Assault by oth specif means occurrn sports/athletic area |
|  |  | U3yy.00 | 365191000006112 | [X]Assault by other specif means occurrn other specif place |
|  |  | U3yz.00 | 365201000006110 | [X]Assault by other specif means occurrn unspecified place |
|  |  | U3y4.00 | 365251000006114 | [X]Assault by othr specif means occurrn on street / highway |
|  |  | U3z2.00 | 365901000006115 | [X]Assault by unspecified means occ sch oth ins/pub adm area |
|  |  | U3zz.00 | 365911000006117 | [X]Assault by unspecified means occurrn at unspecif place |
|  |  | U3z4.00 | 365931000006111 | [X]Assault by unspecified means occurrn on street / highway |
|  |  | U3zy.00 | 365941000006118 | [X]Assault by unspecified means occurrn other specif place |
|  |  | U3z1.00 | 365951000006116 | [X]Assault by unspecified means occurrn resident institut'n |
|  |  | U3L..11 | 424431000006117 | Rape |
|  |  | U3L2.00 | 426671000006114 | [X]Sexual assault by bodil forc occ sch oth ins/pub adm area |
|  |  | U3L6.00 | 426691000006110 | [X]Sexual assault by bodily force occurrn indust/constr area |
|  |  | U3L4.00 | 426701000006110 | [X]Sexual assault by bodily force occurrn on street/highway |
|  |  | U3Ly.00 | 426711000006113 | [X]Sexual assault by bodily force occurrn oth specif place |
|  |  | U3L1.00 | 426721000006117 | [X]Sexual assault by bodily force occurrn resident instit'n |
|  |  | U3L5.00 | 426741000006112 | [X]Sexual assault by bodily force occurrn trade/servce area |
|  |  | U3Lz.00 | 426751000006114 | [X]Sexual assault by bodily force occurrn unspecified place |
|  |  | 13HI.11 | 526241000006112 | Broken home |
|  |  | 13HT111 | 627541000006119 | Domestic stress |
|  |  | 13H4211 | 717161000006113 | Marital discord |
|  |  | 13H4212 | 717171000006118 | Marital disharmony |
|  |  | 13H4.12 | 717221000006112 | Marital stress |
|  |  | 13H4.11 | 717231000006110 | Marital trouble |
|  |  | 13HN200 | 766711000006111 | Forged or altered prescription |
|  |  | TL01.99 | 902751000006116 | Assault: rape |
|  |  | ZV61299 | 903031000006117 | Child sexual abuse |
|  |  | U3P..00 | 1817351000006112 | Maltreatment |
|  |  | U3P0.00 | 1817361000006114 | [X]Maltreatment, by spouse or partner |
|  |  | U3P1.00 | 1817371000006119 | [X]Maltreatment, by parent |
|  |  | U3P2.00 | 1817381000006116 | [X]Maltreatment, by acquaintance or friend |
|  |  | 14XE.00 | 2203601000000111 | History of being victim of domestic violence |
|  |  | 13IZ000 | 2224061000000114 | Lives with adoptive parents |
|  |  | 13IZ100 | 2224301000000113 | Lives with biological parent and step parent |
|  |  | 14XF.00 | 2233011000000112 | Victim of human trafficking |
|  |  | 14XG.00 | 2270041000000118 | Victim of domestic abuse |
|  |  | ^ESCTSE518051 | 5180511000006110 | Sexual assault and rape |
|  |  | ^ESCT1245707 | 12457071000006116 | [V]Child abuse |
| Tics |  | E272200 | 13896014 | Chronic motor tic disorder |
|  |  | E273100 | 73289018 | Head-banging |
|  |  | E272000 | 295399017 | Tic disorder unspecified |
|  |  | E272100 | 295400012 | Transient childhood tic |
|  |  | E272z00 | 295401011 | Tic NOS |
|  |  | E273z00 | 295403014 | Stereotyped repetitive movements NOS |
|  |  | E272.00 | 1491776015 | Tics |
|  |  | E273.00 | 126831000006114 | Repetitive routines |
|  |  | E273200 | 132641000006118 | Spasmus nutans - nodding spasm |
|  |  | E273000 | 519361000006119 | Body rocking |
|  |  | E272300 | 802231000006113 | Gilles de la Tourette's syndrome |
|  |  | E273.99 | 882601000006118 | Stereotyped repetitive movmts. |
