# Supplementary material for: Pre‐Diagnostic Features of Multiple Sclerosis in a Diverse UK Cohort: A Nested Case–Control Study
Source: Ann Clin Transl Neurol. 2025 Sep 24;13(1):71–84. doi: 10.1002/acn3.70175 (PMC12790163; doi:10.1002/acn3.70175)
Supplement: Supplementary file 4 — Table S3: (a) Numbers of MS cases and controls by detailed ethnicity label; (b) Demographic and clinical characteristics of patients by MS status according to duration of retrospective data availability. [file ACN3-13-71-s003.docx]

**Supplementary table 3a:** Numbers of MS cases and controls by detailed ethnicity label

| Ethnicity grouping | | Cases (n=15,029) | Controls (n=81,027) |
| --- | --- | --- | --- |
| Final category | **HES ethnicity** | **N (%)** | **N** |
| White | White | 13288 (88.4) | 48971 (60.4) |
| White | NA | 348 (2.3) | 4139 (5.1) |
| Black | Black African | 77 (0.5) | 828 (1.0) |
| Black | Black Carib | 171 (1.1) | 644 (0.8) |
| Black | Black Other | 84 (0.6) | 448 (0.6) |
| Black | NA | <20 (<0.13)* | 344 (0.4) |
| Asian | Bangladeshi | <20 (<0.13)* | 201 (0.2) |
| Asian | Indian | 176 (1.2) | 1050 (1.3) |
| Asian | Other Asian | 59 (0.4) | 611 (0.8) |
| Asian | Pakistani | 82 (0.5) | 524 (0.6) |
| Asian | NA | <20 (<0.13)* | 500 (0.6) |
| Mixed/Other | Chinese | <20 (<0.13)* | 256 (0.3) |
| Mixed/Other | Mixed | 108 (0.7) | 474 (0.6) |
| Mixed/Other | Other | 146 (1.0) | 1051 (1.3) |
| Mixed/Other | NA | <20 (<0.13)* | 354 (0.4) |
| Unknown | NA | 436 (2.9) | 20632 (25.5) |

*Counts supressed with n<20 in line with CPRD permissions to avoid potential identification

**Supplementary table 3b:** Demographic and Clinical characteristics of patients by MS status according to duration of retrospective data availability

| \|  \| ≥5 years of retrospective data \| \| ≥10 years of retrospective data \| \| \| --- \| --- \| --- \| --- \| --- \| \| Case  N = 15029 \| Control  N = 81027 \| Case  N = 10544 \| Control  N = 50813 \| \| N (%) \| N (%) \| N (%) \| N (%) \| \| Age at Registration (mean (SD)) \| 26.9 (14.7) \| 29.0 (14.1) \| 23.5 (14.6) \| 24.8 (13.8) \| \| Age at Index date (mean (SD)) \| 44.6 (12.3) \| 44.6 (11.6) \| 45.6 (12.5) \| 45.2 (11.7) \| \| Time prior Index date (median [IQR]) \| 14.7 [9.1-23.2] \| 12.4 [8.1-19.5] \| 19.2 [13.9-27.2] \| 17.2 [13.0-24.2] \| \| Gender^1^ \|  \|  \|  \|  \| \| Female \| 10572 (70.3) \| 40651 (50.2) \| 7371 (69.9) \| 25504 (50.2) \| \| Male \| 4457 (29.7) \| 40376 (49.8) \| 3173 (30.1) \| 25309 (49.8) \| \| Ethnicity^1^ \|  \|  \|  \|  \| \| White \| 13636 (90.7) \| 53110 (65.5) \| 9659 (91.6) \| 33048 (65.0) \| \| Black \| 338 ( 2.2) \| 2264 ( 2.8) \| 209 ( 2.0) \| 1065 ( 2.1) \| \| Asian \| 349 ( 2.3) \| 2886 ( 3.6) \| 215 ( 2.0) \| 1435 ( 2.8) \| \| Mixed/Other \| 270 ( 1.8) \| 2135 ( 2.6) \| 161 ( 1.5) \| 985 ( 1.9) \| \| Unknown \| 436 ( 2.9) \| 20632 (25.5) \| 300 ( 2.8) \| 14280 (28.1) \| \| IMD (Patient level)^1^ \|  \|  \|  \|  \| \| 1 \| 3486 (23.2) \| 17432 (21.5) \| 2450 (23.2) \| 11099 (21.8) \| \| 2 \| 3454 (23.0) \| 16813 (20.7) \| 2382 (22.6) \| 10620 (20.9) \| \| 3 \| 3017 (20.1) \| 16033 (19.8) \| 2126 (20.2) \| 10028 (19.7) \| \| 4 \| 2792 (18.6) \| 16350 (20.2) \| 1968 (18.7) \| 10051 (19.8) \| \| 5 \| 2280 (15.2) \| 14399 (17.8) \| 1618 (15.3) \| 9015 (17.7) \| \| IMD (Practice level)^2^ \|  \|  \|  \|  \| \| 1 \| 2699 (18.0) \| 14155 (17.5) \| 1870 (17.7) \| 8808 (17.3) \| \| 2 \| 2704 (18.0) \| 13803 (17.0) \| 1899 (18.0) \| 8588 (16.9) \| \| 3 \| 3307 (22.0) \| 17252 (21.3) \| 2279 (21.6) \| 10743 (21.1) \| \| 4 \| 3153 (21.0) \| 18300 (22.6) \| 2208 (20.9) \| 11416 (22.5) \| \| 5 \| 3166 (21.1) \| 17517 (21.6) \| 2288 (21.7) \| 11258 (22.2) \| \| Location^1^ \|  \|  \|  \|  \| \| Urban \| 12263 (81.6) \| 68408 (84.4) \| 8564 (81.2) \| 42692 (84.0) \| \| Rural \| 2766 (18.4) \| 12619 (15.6) \| 1980 (18.8) \| 8121 (16.0) \| \| Autonomic symptoms^3^ \| 5751 (38.3) \| 16672 (20.6) \| 4244 (40.3) \| 10462 (20.6) \| \| Cognitive symptoms^3^ \| 130 (0.9) \| 244 (0.3) \| 98 ( 0.9) \| 145 ( 0.3) \| \| Neurological symptoms^3^ \| 6664 (44.3) \| 6096 (7.5) \| 4820 (45.7) \| 3799 ( 7.5) \| \| Pain symptoms^3^ \| 6515 (43.3) \| 17102 (21.1) \| 4731 (44.9) \| 10584 (20.8) \| \| Psychiatric symptoms^3^ \| 5176 (34.4) \| 14617 (18.0) \| 3702 (35.1) \| 8661 (17.0) \| |
| --- | --- | --- | --- | --- | --- | --- | --- | --- | --- | --- | --- | --- | --- | --- | --- | --- | --- | --- | --- | --- | --- | --- | --- | --- | --- | --- | --- | --- | --- | --- | --- | --- | --- | --- | --- | --- | --- | --- | --- | --- | --- | --- | --- | --- | --- | --- | --- | --- | --- | --- | --- | --- | --- | --- | --- | --- | --- | --- | --- | --- | --- | --- | --- | --- | --- | --- | --- | --- | --- | --- | --- | --- | --- | --- | --- | --- | --- | --- | --- | --- | --- | --- | --- | --- | --- | --- | --- | --- | --- | --- | --- | --- | --- | --- | --- | --- | --- | --- | --- | --- | --- | --- | --- | --- | --- | --- | --- | --- | --- | --- | --- | --- | --- | --- | --- | --- | --- | --- | --- | --- | --- | --- | --- | --- | --- | --- | --- | --- | --- | --- | --- | --- | --- | --- | --- | --- | --- | --- | --- | --- | --- | --- | --- | --- | --- | --- | --- | --- | --- | --- | --- | --- | --- | --- | --- | --- | --- | --- | --- | --- | --- | --- | --- | --- | --- | --- | --- | --- | --- | --- | --- | --- | --- |

*Continuous variables are presented as mean ± SD for normally distributed variables and as median (interquartile range) for skewed variables. Categorical data was presented as counts and percentages (%).*
