# Supplementary material for: Pre‐Diagnostic Features of Multiple Sclerosis in a Diverse UK Cohort: A Nested Case–Control Study
Source: Ann Clin Transl Neurol. 2025 Sep 24;13(1):71–84. doi: 10.1002/acn3.70175 (PMC12790163; doi:10.1002/acn3.70175)
Supplement: Supplementary file 5 — Table S4: Missing data table, characteristics for those who have at least 10 years vs. those who only have 5 years prior index date. Table S5: Results from a multivariable logistic regression (adjusted) model examining the interaction between pre‐diagnostic symptoms and demographic subgroups on the risk. Table S6: Comparing the interaction between demographic backgrounds and pre‐diagnostic symptoms by periods of different MS diagnosis criteria. Table S7: Prodromal symptoms according to codes used to define MS. [file ACN3-13-71-s001.docx]

**Supplementary table 4:** Missing data table, Characteristics for those who have at least 10 years vs those who only have 5 years prior index date.

|  | Present | Missing |
| --- | --- | --- |
|  | ≥10 years pre-diagnostic years | 5 pre-diagnostic years |
| N | 65226 | 36388 |
| Age at Registration (mean (SD)) | 24.48 (13.96) | 35.91 (11.37) |
| Age at index date (mean (SD)) | 45.20 (11.85) | 43.28 (11.29) |
| Follow Up time from index date (mean (SD)) | 20.67 (10.19) | 7.33 (1.44) |
| Status = Control (%) | 54420 (83.4) | 31795 (87.4) |
| Gender: Female/ Male (%) | 34968/30258 (53.6/46.4) | 19263/17125 (52.9/47.1) |
| Ethnicity (%) |  |  |
| White | 43259 (66.3) | 24429 (67.1) |
| Black | 1303 ( 2.0) | 1355 ( 3.7) |
| Asian | 1686 ( 2.6) | 1622 ( 4.5) |
| Mixed/Other | 1153 ( 1.8) | 1276 ( 3.5) |
| Unknown | 17825 (27.3) | 7706 (21.2) |
| Dep quantile (%) |  |  |
| 1 | 13549 (20.8) | 7369 (20.3) |
| 2 | 13002 (19.9) | 7265 (20.0) |
| 3 | 12154 (18.6) | 6896 (19.0) |
| 4 | 12019 (18.4) | 7123 (19.6) |
| 5 | 10633 (16.3) | 6046 (16.6) |
| Unknown | 3869 ( 5.9) | 1689 ( 4.6) |
| Location (%) |  |  |
| Urban | 51256 (78.6) | 29415 (80.8) |
| Rural | 10101 (15.5) | 5284 (14.5) |
| Unknown | 3869 ( 5.9) | 1689 ( 4.6) |
| Autonomic symptoms (%) | 8664(13.3) | 5104(14.0) |
| Cognitive symptoms (%) | 146(0.2) | 81(0.2) |
| Neurological symptoms (%) | 5958(9.1) | 3019(8.3) |
| Pain symptoms (%) | 8574(13.1) | 5337(14.7) |
| Psychiatric symptoms (%) | 6350(9.7) | 4163(11.4) |

**Supplementary table 5:** Results from a multivariable logistic regression (adjusted) model examining the interaction between pre-diagnostic symptoms and demographic subgroups on the risk.

Data was modelled for symptoms present at 0-2, 2-5, 5-10, and all pre-diagnostic years adjusting for matching variable age and gender, ethnicity, IMD(patient level) and location.

Model: MS status ~ Age + Gender + Ethnicity + IMD(patient level) + Location + Symptom x demographic variable (adjusted model).Odd ratios(OR) and 95% confidence intervals(CI) are reported. ORs interpreted as the odds of developing subsequent MS with the given demographic background and symptom compared to the absence of that symptom at x years prior diagnosis. P-values and Bonferroni corrected p-values are reported for symptom*demographic variable interaction p-values. *P-values considered statistically significant. – Counts too low to measure OR. IMD = index level of multiple deprivation.

|  |  | All pre-diagnostic years | | | | 0-2 yrs prior diagnosis | | | | 2-5 yrs prior diagnosis | | | | 5-10 yrs prior diagnosis | | | |
| --- | --- | --- | --- | --- | --- | --- | --- | --- | --- | --- | --- | --- | --- | --- | --- | --- | --- |
|  |  | Case : Control | Adjusted OR (95%CI) | P-int | Bonferroni corrected P-int | Case : Control | Adjusted OR (95%CI) | P-int | Bonferroni corrected P-int | Case : Control | Adjusted OR (95%CI) | P-int | Bonferroni corrected P-int | Case : Control | Adjusted OR (95%CI) | P-int | Bonferroni corrected P-int |
| **Symptoms** | **Gender** | |  |  |  |  |  |  |  |  |  |  |  |  |  |  |  |
| Autonomic symptoms | Female | 4194 : 9429 | 1.81 (1.73-1.89)* | 0.0095* | 0.1895 | 1791 : 3008 | 2.14 (2.01-2.28)* | 0.0024* | 0.1459 | 1780 : 3650 | 1.71 (1.60-1.82)* | 0.1898 | ~1 | 1812 : 3990 | 1.77 (1.64-1.91)* | 0.9385 | ~1 |
|  | Male | 1557 : 7243 | 2.02 (1.88-2.16)* |  |  | 718 : 2366 | 2.54 (2.32-2.79)* |  |  | 642 : 2802 | 1.84 (1.67-2.02)* |  |  | 589 : 2972 | 1.76 (1.57-1.97)* |  |  |
| Cognitive symptoms | Female | 89 : 121 | 2.57 (1.94-3.39)* | 0.9951 | ~1 | 48 : 40 | 4.34 (2.82-6.69)* | 0.3135 | ~1 | 19 : 34 | 1.87 (1.06-3.30)* | 0.7409 | ~1 | 17 : 30 | 2.12 (1.00-4.49)* | 0.6570 | ~1 |
|  | Male | 41 : 123 | 2.56 (1.79-3.67)* |  |  | 26 : 32 | 6.18 (3.64-10.49)* |  |  | 7 : 35 | 1.58 (0.70-3.60) |  |  | 6 : 38 | 1.60 (0.59-4.34) |  |  |
| Neurological symptoms | Female | 4690 : 3528 | 7.05 ( 6.69-7.44)* | <0.0001* | <0.0001* | 3323 : 961 | 15.92 (14.74-17.21)* | <0.0001* | <0.0001* | 1235 : 1190 | 3.68 ( 3.38-4.00)* | 0.0019* | 0.1168* | 1043 : 1261 | 3.11 ( 2.80-3.45)* | 0.0007* | 0.0419* |
|  | Male | 1974 : 2568 | 9.84 ( 9.15-10.58)* |  |  | 1425 : 688 | 22.54 (20.38-24.92)* |  |  | 472 : 826 | 4.63 ( 4.11-5.22)* |  |  | 390 : 823 | 4.27 ( 3.67-4.97)* |  |  |
| Pain symptoms | Female | 4754 : 9564 | 2.17 (2.07-2.27)* | 0.1786 | ~1 | 2266 : 2743 | 3.10 (2.92-3.30)* | 0.0451* | ~1 | 2108 : 3533 | 2.15 (2.03-2.28)* | 0.6886 | ~1 | 2073 : 4232 | 1.96 (1.83-2.10)* | 0.5802 | ~1 |
|  | Male | 1761 : 7538 | 2.29 (2.14-2.45)* |  |  | 792 : 1897 | 3.47 (3.17-3.80)* |  |  | 683 : 2492 | 2.20 (2.01-2.41)* |  |  | 675 : 2994 | 2.03 (1.83-2.27)* |  |  |
| Psychiatric symptoms | Female | 3998 : 9005 | 1.73 (1.65-1.81)* | 0.2753 | ~1 | 1730 : 2356 | 2.53 (2.36-2.70)* | 0.0360* | ~1 | 1807 : 3042 | 2.07 (1.94-2.20)* | 0.6149 | ~1 | 1768 : 3913 | 1.78 (1.65-1.92)* | 0.4731 | ~1 |
|  | Male | 1178 : 5612 | 1.82 (1.69-1.95)* |  |  | 489 : 1307 | 2.90 (2.60-3.24)* |  |  | 476 : 1718 | 2.13 (1.92-2.38)* |  |  | 459 : 2248 | 1.88 (1.65-2.15)* |  |  |
| **Symptoms** | **Ethnicity** | |  |  |  |  |  |  |  |  |  |  |  |  |  |  |  |
| Autonomic symptoms | White | 5233 : 13395 | 1.81 (1.74-1.89)* | <0.0001* | 0.0002* | 2291 : 4584 | 2.18 (2.06-2.30)* | <0.0001* | <0.0001* | 2214 : 5384 | 1.70 (1.61-1.80)* | 0.0005* | 0.0295* | 2181 : 5566 | 1.74 (1.63-1.85)* | 0.0097* | 0.5817 |
|  | Black | 172 : 628 | 2.57 (2.03-3.25)* |  |  | 68 : 196 | 2.54 (1.87-3.45)* |  |  | 74 : 243 | 2.26 (1.68-3.03)* |  |  | 85 : 323 | 2.51 (1.74-3.61)* |  |  |
|  | Asian | 154 : 735 | 2.24 (1.78-2.82)* |  |  | 74 : 229 | 3.13 (2.33-4.20)* |  |  | 58 : 308 | 1.64 (1.21-2.24)* |  |  | 59 : 347 | 1.31 (0.89-1.94) |  |  |
|  | Mixed / Other | 119 : 443 | 2.94 (2.26-3.83)* |  |  | 42 : 117 | 3.16 (2.16-4.64)* |  |  | 49 : 164 | 2.55 (1.79-3.62)* |  |  | 53 : 207 | 3.07 (1.99-4.74)* |  |  |
|  | Unknown | 73 : 1471 | 2.60 (2.01-3.37)* |  |  | 34 : 248 | 7.69 (5.28-11.19)* |  |  | 27 : 353 | 3.83 (2.55-5.75)* |  |  | 23 : 519 | 2.44 (1.45-4.09)* |  |  |
| Cognitive symptoms | White | 114 : 195 | 2.45 (1.93-3.1)* | 0.1807 | ~1 | 62 : 59 | 4.40 (3.05-6.33)* | 0.1117 | ~1 | 25 : 54 | 1.90 (1.17-3.07)* | 0.5152 | ~1 | 22 : 53 | 2.07 (1.11-3.87)* | 0.5670 | ~1 |
|  | Black | 7 : 7 | 5.89 (2.03-17.07)* |  |  | 4 : 3 | 8.07 (1.77-36.8)* |  |  | 1 : 2 | 2.68 (0.24-29.64) |  |  | 1 : 1 | 4.00 (0.25-64.18) |  |  |
|  | Asian | 4 : 16 | 1.95 (0.64-5.96) |  |  | 3 : 2 | 12.57 (2.03-77.82)* |  |  | 0 : 5 | – |  |  | 0 : 7 | – |  |  |
|  | Mixed / Other | 2 : 10 | 1.79 (0.38-8.37) |  |  | 2 : 4 | 3.45 (0.62-19.11) |  |  | 0 : 3 | – |  |  | 0 : 4 | – |  |  |
|  | Unknown | 3 : 16 | 9.97 (2.85-34.86)* |  |  | 3 : 4 | 37.45 (8.10-173.15)* |  |  | 0 : 5 | – |  |  | 0 : 3 | – |  |  |
| Neurological symptoms | White | 6079 : 5051 | 7.58 ( 7.24-7.93)* | <0.0001* | <0.0001* | 4324 : 1371 | 17.31 (16.21-18.49)* | <0.0001* | <0.0001* | 1551 : 1666 | 3.88 ( 3.61-4.18)* | 0.0099* | 0.5964 | 1323 : 1693 | 3.42 ( 3.13-3.75)* | 0.1334 | ~1 |
|  | Black | 172 : 196 | 10.49 ( 8.07-13.63)* |  |  | 121 : 56 | 20.70 (14.57-29.39)* |  |  | 50 : 67 | 5.28 ( 3.57-7.81)* |  |  | 42 : 77 | 3.94 ( 2.28-6.81)* |  |  |
|  | Asian | 175 : 296 | 8.56 ( 6.70-10.93)* |  |  | 130 : 107 | 15.23 (11.33-20.46)* |  |  | 51 : 133 | 3.37 ( 2.38-4.78)* |  |  | 27 : 107 | 2.16 ( 1.25-3.73)* |  |  |
|  | Mixed / Other | 146 : 167 | 13.98 (10.46-18.70)* |  |  | 105 : 37 | 37.30 (24.68-56.37)* |  |  | 39 : 71 | 4.92 ( 3.23-7.50)* |  |  | 29 : 70 | 3.97 ( 2.29-6.88)* |  |  |
|  | Unknown | 92 : 386 | 14.82 (11.50-19.11)* |  |  | 68 : 78 | 51.44 (36.33-72.85)* |  |  | 16 : 79 | 10.11 ( 5.82-17.57)* |  |  | 12 : 137 | 6.74 ( 3.54-12.86)* |  |  |
| Pain symptoms | White | 5927 : 13618 | 2.14 (2.06-2.23)* | <0.0001* | 0.0006* | 2776 : 3889 | 3.10 (2.94-3.27)* | <0.0001* | <0.0001* | 2525 : 4948 | 2.10 (1.99-2.21)* | <0.0001* | 0.0032* | 2476 : 5719 | 1.94 (1.82-2.06)* | 0.0189* | ~1 |
|  | Black | 180 : 614 | 2.97 (2.35-3.76)* |  |  | 91 : 166 | 4.45 (3.32-5.95)* |  |  | 81 : 249 | 2.48 (1.86-3.30)* |  |  | 80 : 301 | 2.21 (1.52-3.23)* |  |  |
|  | Asian | 189 : 835 | 2.71 (2.16-3.40)* |  |  | 98 : 280 | 3.38 (2.59-4.42)* |  |  | 99 : 365 | 2.59 (2.00-3.36)* |  |  | 96 : 400 | 1.96 (1.40-2.73)* |  |  |
|  | Mixed / Other | 138 : 480 | 3.48 (2.68-4.52)* |  |  | 56 : 113 | 4.45 (3.12-6.34)* |  |  | 53 : 168 | 2.73 (1.94-3.85)* |  |  | 71 : 232 | 3.37 (2.27-5.01)* |  |  |
|  | Unknown | 81 : 1555 | 2.76 (2.15-3.53)* |  |  | 37 : 192 | 9.66 (6.68-13.97)* |  |  | 33 : 295 | 5.49 (3.77-7.99)* |  |  | 25 : 574 | 3.36 (2.10-5.37)* |  |  |
| Psychiatric symptoms | White | 4778 : 12036 | 1.70 (1.63-1.77)* | <0.0001* | <0.0001* | 2050 : 3249 | 2.49 (2.34-2.64)* | <0.0001* | <0.0001* | 2099 : 4096 | 1.99 (1.88-2.11)* | <0.0001* | 0.0002* | 2051 : 5133 | 1.75 (1.63-1.87)* | 0.0101* | 0.6079 |
|  | Black | 125 : 406 | 2.52 (1.96-3.23)* |  |  | 52 : 85 | 4.30 (2.97-6.23)* |  |  | 62 : 132 | 3.52 (2.53-4.90)* |  |  | 61 : 177 | 2.58 (1.69-3.93)* |  |  |
|  | Asian | 115 : 426 | 2.61 (2.04-3.34)* |  |  | 47 : 99 | 4.14 (2.85-6.00)* |  |  | 55 : 145 | 3.18 (2.27-4.46)* |  |  | 51 : 184 | 2.36 (1.52-3.68)* |  |  |
|  | Mixed / Other | 94 : 354 | 2.49 (1.89-3.29)* |  |  | 43 : 73 | 5.23 (3.48-7.85)* |  |  | 42 : 108 | 3.28 (2.23-4.82)* |  |  | 47 : 164 | 3.57 (2.24-5.69)* |  |  |
|  | Unknown | 64 : 1395 | 2.27 (1.73-2.97)* |  |  | 27 : 157 | 8.33 (5.46-12.73)* |  |  | 25 : 279 | 4.05 (2.65-6.18)* |  |  | 17 : 503 | 2.32 (1.28-4.20)* |  |  |
| **Symptoms** | **Grouped IMD (patient level)** | | |  |  |  |  |  |  |  |  |  |  |  |  |  |  |
| Autonomic symptoms | IMD 1-2 | 2614 : 7046 | 1.81 (1.71-1.92)* | 0.3144 | ~1 | 1123 : 2306 | 2.12 (1.96-2.30)* | 0.0097* | 0.5824 | 1098 : 2772 | 1.67 (1.54-1.80)* | 0.2778 | ~1 | 1080 : 2878 | 1.66 (1.51-1.82)* | 0.1229 | ~1 |
|  | IMD 3 | 1164 : 3257 | 1.93 (1.77-2.10)* |  |  | 500 : 1096 | 2.15 (1.91-2.42)* |  |  | 497 : 1264 | 1.80 (1.61-2.02)* |  |  | 490 : 1354 | 1.97 (1.71-2.26)* |  |  |
|  | IMD 4-5 | 1973 : 6369 | 1.92 (1.80-2.05)* |  |  | 886 : 1972 | 2.53 (2.31-2.76)* |  |  | 827 : 2416 | 1.82 (1.67-1.99)* |  |  | 831 : 2730 | 1.80 (1.63-2.00)* |  |  |
| Cognitive symptoms | IMD 1-2 | 46 : 91 | 2.36 (1.63-3.41)* | 0.8038 | ~1 | 28 : 30 | 4.44 (2.60-7.58)* | 0.4516 | ~1 | 6 : 31 | 0.85 (0.35-2.08) | 0.0092* | 0.5495 | 11 : 26 | 2.21 (0.88-5.56) | 0.7249 | ~1 |
|  | IMD 3 | 28 : 48 | 2.50 (1.55-4.05)* |  |  | 21 : 13 | 7.58 (3.60-15.94)* |  |  | 2 : 12 | 0.70 (0.15-3.16) |  |  | 3 : 9 | 2.79 (0.61-12.77) |  |  |
|  | IMD 4-5 | 56 : 105 | 2.79 (1.99-3.90)* |  |  | 25 : 29 | 4.46 (2.57-7.76)* |  |  | 18 : 26 | 3.58 (1.92-6.69)* |  |  | 9 : 33 | 1.48 (0.60-3.65) |  |  |
| Neurological symptoms | IMD 1-2 | 3072 : 2602 | 7.68 ( 7.20-8.19)* | 0.4546 | ~1 | 2154 : 666 | 18.33 (16.67-20.16)* | 0.9841 | ~1 | 790 : 841 | 4.00 ( 3.60-4.44)* | 0.6002 | ~1 | 666 : 882 | 3.39 ( 2.98-3.86)* | 0.9484 | ~1 |
|  | IMD 3 | 1347 : 1202 | 8.18 ( 7.43-9.01)* |  |  | 964 : 329 | 18.13 (15.79-20.81)* |  |  | 340 : 419 | 3.70 ( 3.18-4.32)* |  |  | 280 : 407 | 3.53 ( 2.90-4.29)* |  |  |
|  | IMD 4-5 | 2245 : 2292 | 8.06 ( 7.50-8.67)* |  |  | 1630 : 654 | 18.11 (16.37-20.04)* |  |  | 577 : 756 | 4.08 ( 3.63-4.59)* |  |  | 487 : 795 | 3.44 ( 2.98-3.98)* |  |  |
| Pain symptoms | IMD 1-2 | 2933 : 7195 | 2.11 (1.99-2.23)* | 0.0766 | ~1 | 1324 : 1996 | 2.89 (2.67-3.12)* | 0.0012* | 0.0711 | 1250 : 2412 | 2.18 (2.02-2.35)* | 0.9437 | ~1 | 1186 : 2938 | 1.81 (1.65-1.98)* | 0.0196* | ~1 |
|  | IMD 3 | 1353 : 3405 | 2.35 (2.16-2.55)* |  |  | 635 : 858 | 3.56 (3.18-3.99)* |  |  | 560 : 1204 | 2.13 (1.90-2.38)* |  |  | 570 : 1439 | 2.21 (1.94-2.52)* |  |  |
|  | IMD 4-5 | 2229 : 6502 | 2.26 (2.12-2.40)* |  |  | 1099 : 1786 | 3.46 (3.18-3.77)* |  |  | 981 : 2409 | 2.17 (2.00-2.36)* |  |  | 992 : 2849 | 2.09 (1.89-2.30)* |  |  |
| Psychiatric symptoms | IMD 1-2 | 2241 : 5798 | 1.69 (1.59-1.79)* | 0.1462 | ~1 | 933 : 1469 | 2.43 (2.22-2.66)* | 0.0549 | ~1 | 948 : 1886 | 1.93 (1.78-2.11)* | 0.0743 | ~1 | 928 : 2354 | 1.67 (1.50-1.85)* | 0.0980 | ~1 |
|  | IMD 3 | 1056 : 2766 | 1.88 (1.72-2.05)* |  |  | 444 : 716 | 2.62 (2.31-2.99)* |  |  | 468 : 887 | 2.24 (1.98-2.53)* |  |  | 465 : 1163 | 2.01 (1.73-2.33)* |  |  |
|  | IMD 4-5 | 1879 : 6053 | 1.77 (1.65-1.89)* |  |  | 842 : 1478 | 2.85 (2.59-3.12)* |  |  | 867 : 1987 | 2.18 (1.99-2.38)* |  |  | 834 : 2644 | 1.86 (1.68-2.07)* |  |  |
| **Symptoms** | **Location (Urban/ Rural)** | | |  |  |  |  |  |  |  |  |  |  |  |  |  |  |
| Autonomic symptoms | Rural | 1031 : 2809 | 1.68 (1.53-1.83)* | 0.0080* | 0.1599 | 424 : 956 | 1.79 (1.58-2.04)* | <0.0001* | 0.0034* | 445 : 1122 | 1.60 (1.42-1.81)* | 0.1242 | ~1 | 427 : 1103 | 1.73 (1.50-2.01)* | 0.7771 | ~1 |
|  | Urban | 4720 : 13863 | 1.92 (1.84-2.00)* |  |  | 2085 : 4418 | 2.38 (2.24-2.52)* |  |  | 1977 : 5330 | 1.78 (1.68-1.88)* |  |  | 1974 : 5859 | 1.78 (1.66-1.90)* |  |  |
| Cognitive symptoms | Rural | 15 : 35 | 1.70 (0.92-3.15) | 0.1508 | ~1 | 6 : 15 | 1.50 (0.57-3.95) | 0.0056* | 0.3365 | 1 : 12 | 0.37 (0.05-2.85) | 0.0477* | ~1 | 4 : 8 | 2.61 (0.61-11.23) | 0.6538 | ~1 |
|  | Urban | 115 : 209 | 2.74 (2.16-3.47)* |  |  | 68 : 57 | 6.08 (4.21-8.78)* |  |  | 25 : 57 | 2.09 (1.29-3.40)* |  |  | 19 : 60 | 1.80 (0.94-3.44) |  |  |
| Neurological symptoms | Rural | 1215 : 1070 | 7.00 ( 6.33-7.75)* | 0.0088* | 0.1765 | 877 : 276 | 17.40 (15.00-20.19)* | 0.5137 | ~1 | 301 : 355 | 3.34 ( 2.84-3.94)* | 0.0239* | ~1 | 261 : 340 | 3.27 ( 2.66-4.03)* | 0.6149 | ~1 |
|  | Urban | 5449 : 5026 | 8.13 ( 7.75-8.52)* |  |  | 3871 : 1373 | 18.38 (17.17-19.68)* |  |  | 1406 : 1661 | 4.12 ( 3.81-4.44)* |  |  | 1172 : 1744 | 3.47 ( 3.16-3.82)* |  |  |
| Pain symptoms | Rural | 1146 : 2801 | 1.94 (1.78-2.12)* | 0.0018* | 0.0364* | 519 : 753 | 2.80 (2.47-3.17)* | 0.0157* | 0.9403 | 476 : 970 | 1.89 (1.68-2.14)* | 0.0170* | ~1 | 482 : 1137 | 1.74 (1.51-2.01)* | 0.0524 | ~1 |
|  | Urban | 5369 : 14301 | 2.27 (2.18-2.36)* |  |  | 2539 : 3887 | 3.31 (3.13-3.50)* |  |  | 2315 : 5055 | 2.23 (2.11-2.35)* |  |  | 2266 : 6089 | 2.04 (1.91-2.17)* |  |  |
| Psychiatric symptoms | Rural | 917 : 2381 | 1.59 (1.45-1.75)* | 0.0255* | 0.5106 | 378 : 636 | 2.16 (1.88-2.48)* | 0.0026* | 0.1534 | 367 : 740 | 1.81 (1.58-2.08)* | 0.0275* | ~1 | 403 : 940 | 1.83 (1.56-2.14)* | 0.8591 | ~1 |
|  | Urban | 4259 : 12236 | 1.79 (1.71-1.87)* |  |  | 1841 : 3027 | 2.73 (2.56-2.91)* |  |  | 1916 : 4020 | 2.14 (2.02-2.27)* |  |  | 1824 : 5221 | 1.80 (1.67-1.94)* |  |  |

**Supplementary table 6:** Comparing the interaction between demographic backgrounds and pre-diagnostic symptoms by periods of different MS diagnosis criteria.

| Data was modelled for symptoms present at all pre-diagnostic years adjusting for matching variable age and gender from a univariate logistic regression. Model: MS status ~ Age + Gender + Symptom x demographic variable (unadjusted model). Odd ratios(OR) and 95% confidence intervals(CI) are reported. P-values and Bonferroni corrected p-values are reported for symptom*demographic variable interaction p-values. *P-values considered statistically significant. – Counts too low to measure OR. | | | | | | | | | | | | | |
| --- | --- | --- | --- | --- | --- | --- | --- | --- | --- | --- | --- | --- | --- |
|  |  | Jan-1990 - Dec-2000 | | | Jan-2001 - Dec-2010 | | | Jan-2011 - Mar-2022 | | | All cases and controls | | |
| Grouped symptoms | Sub category | Unadjusted OR (95%CI) | P-int | Bonferroni corrected P-int | Unadjusted OR (95%CI) | P-int | Bonferroni corrected P-int | Unadjusted OR (95%CI) | P-int | Bonferroni corrected P-int | Unadjusted OR (95%CI) | P-int | Bonferroni corrected P-int |
| **Gender** |  |  |  |  |  |  |  |  |  |  |  |  |  |
| Autonomic symptoms | Male | 1.63 (1.38-1.91) | 0.0156* | ~1 | 2.44 (2.19-2.72) | 0.0852 | ~1 | 3.19 (2.87-3.56) | 0.3542 | ~1 | 2.51 (2.35-2.69) | 0.0019* | 0.0387* |
|  | Female | 1.28 (1.15-1.43) |  |  | 2.18 (2.02-2.34) |  |  | 3.40 (3.15-3.68) |  |  | 2.21 (2.11-2.31) |  |  |
| Cognitive symptoms | Male | 6.37 (2.63-15.42) | 0.3193 | ~1 | 2.05 (1.10-3.83) | 0.9769 | ~1 | 2.74 (1.65-4.56) | 0.7821 | ~1 | 3.02 (2.12-4.31) | 0.7740 | ~1 |
|  | Female | 3.45 (1.54-7.73) |  |  | 2.02 (1.25-3.28) |  |  | 3.00 (2.06-4.37) |  |  | 2.83 (2.15-3.73) |  |  |
| Neurological symptoms | Male | 9.89 ( 8.42-11.60) | 0.0002* | 0.0093* | 11.65 (10.38-13.07) | 0.0001* | 0.0067* | 15.15 (13.44-17.09) | <0.0001* | 0.0049* | 12.01 (11.18-12.90) | <0.0001* | <0.0001* |
|  | Female | 6.73 ( 5.98-7.57) |  |  | 8.82 ( 8.12-9.58) |  |  | 11.26 (10.31-12.30) |  |  | 8.52 ( 8.09-8.97) |  |  |
| Pain symptoms | Male | 1.93 (1.65-2.26) | 0.0491* | ~1 | 2.56 (2.30-2.85) | 0.6047 | ~1 | 3.93 (3.52-4.38) | 0.2768 | ~1 | 2.85 (2.67-3.04) | 0.0857 | ~1 |
|  | Female | 1.60 (1.44-1.76) |  |  | 2.65 (2.46-2.84) |  |  | 4.23 (3.91-4.59) |  |  | 2.66 (2.54-2.78) |  |  |
| Psychiatric symptoms | Male | 1.86 (1.57-2.21) | 0.0098* | 0.5885 | 2.20 (1.95-2.48) | 0.9432 | ~1 | 2.32 (2.08-2.60) | 0.0060* | 0.3585 | 2.23 (2.07-2.40) | 0.3504 | ~1 |
|  | Female | 1.43 (1.29-1.58) |  |  | 2.19 (2.03-2.35) |  |  | 2.81 (2.60-3.04) |  |  | 2.14 (2.04-2.24) |  |  |
| **Ethnicity** |  |  |  |  |  |  |  |  |  |  |  |  |  |
| Autonomic symptoms | White | 1.18 (1.07-1.29) | 0.0535 | ~1 | 1.68 ( 1.57-1.79) | 0.0007* | 0.0369* | 2.61 (2.43-2.80) | 0.0771 | ~1 | 1.81 (1.74-1.88) | <0.0001* | 0.0002* |
|  | Black | 1.69 (0.74-3.88) |  |  | 2.00 ( 1.33-3.00) |  |  | 3.19 (2.32-4.39) |  |  | 2.56 (2.02-3.23) |  |  |
|  | Asian | 1.19 (0.40-3.6) |  |  | 1.46 ( 0.97-2.20) |  |  | 2.68 (1.99-3.62) |  |  | 2.23 (1.78-2.81) |  |  |
|  | Mixed/ Other | 1.88 (0.78-4.52) |  |  | 2.01 ( 1.25-3.25) |  |  | 3.94 (2.76-5.61) |  |  | 2.92 (2.24-3.80) |  |  |
|  | Unknown | 2.01 (1.42-2.83) |  |  | 5.73 ( 3.42-9.62) |  |  | 4.64 (2.30-9.38) |  |  | 2.61 (2.01-3.37) |  |  |
| Cognitive symptoms | White | 4.09 (2.10-7.97) | 0.4330 | ~1 | 1.60 (1.07-2.4) | 0.4904 | ~1 | 2.47 (1.77-3.46) | 0.2775 | ~1 | 2.40 (1.90-3.05) | 0.1768 | ~1 |
|  | Black | - |  |  | 1431221.60 (0.00-Inf) |  |  | 4.44 (1.46-13.53) |  |  | 5.80 (2.00-16.81) |  |  |
|  | Asian | 0.00 (0.00-Inf) |  |  | 2.38 (0.48-11.87) |  |  | 1.51 (0.31-7.28) |  |  | 1.98 (0.65-6.03) |  |  |
|  | Mixed/ Other | - |  |  | 0.00 (0.00-Inf) |  |  | 1.70 (0.36-8.01) |  |  | 1.72 (0.37-8.05) |  |  |
|  | Unknown | 10.45 (1.98-55.27) |  |  | 0.00 (0.00-Inf) |  |  | 44.45 (3.76-525.94) |  |  | 9.93 (2.85-34.62) |  |  |
| Neurological symptoms | White | 6.36 ( 5.74-7.05) | 0.0245* | ~1 | 7.29 ( 6.79-7.83) | 0.0024* | 0.1423 | 10.01 ( 9.26-10.83) | <0.0001* | <0.0001* | 7.59 ( 7.25-7.94) | <0.0001* | <0.0001* |
|  | Black | 6.92 ( 2.72-17.59) |  |  | 8.35 ( 5.30-13.16) |  |  | 13.15 ( 9.25-18.69) |  |  | 10.48 ( 8.07-13.62) |  |  |
|  | Asian | 12.53 (3.83-41.02) |  |  | 8.18 ( 5.40-12.40) |  |  | 7.98 ( 5.80-10.99) |  |  | 8.55 ( 6.69-10.92) |  |  |
|  | Mixed/ Other | 11.43 (4.23-30.87) |  |  | 15.15 ( 9.06-25.33) |  |  | 14.20 ( 9.65-20.89) |  |  | 13.82 (10.34-18.48) |  |  |
|  | Unknown | 11.44 (7.99-16.39) |  |  | 17.90 (10.25-31.28) |  |  | 87.13 (44.60-170.22) |  |  | 14.92 (11.58-19.23) |  |  |
| Pain symptoms | White | 1.43 (1.31-1.56) | 0.4783 | ~1 | 1.96 (1.83-2.08) | 0.0036* | 0.2166 | 3.20 ( 2.98-3.44) | 0.0014* | 0.0861 | 2.14 (2.06-2.23) | <0.0001* | 0.0007* |
|  | Black | 1.45 (0.55-3.81) |  |  | 2.50 (1.65-3.78) |  |  | 3.78 ( 2.72-5.26) |  |  | 2.96 (2.34-3.75) |  |  |
|  | Asian | 1.80 (0.69-4.72) |  |  | 1.73 (1.17-2.55) |  |  | 3.36 ( 2.46-4.57) |  |  | 2.71 (2.16-3.40) |  |  |
|  | Mixed/ Other | 1.89 (0.78-4.56) |  |  | 2.73 (1.72-4.33) |  |  | 4.63 ( 3.23-6.65) |  |  | 3.43 (2.64-4.46) |  |  |
|  | Unknown | 1.99 (1.40-2.83) |  |  | 5.19 (3.12-8.63) |  |  | 10.71 ( 5.72-20.07) |  |  | 2.77 (2.17-3.55) |  |  |
| Psychiatric symptoms | White | 1.29 (1.18-1.42) | 0.6260 | ~1 | 1.64 (1.53-1.75) | 0.0046* | 0.2778 | 1.99 (1.86-2.13) | 0.0004* | 0.0253* | 1.68 (1.61-1.75) | <0.0001* | <0.0001* |
|  | Black | 1.13 (0.40-3.14) |  |  | 2.16 (1.39-3.37) |  |  | 2.97 (2.15-4.10) |  |  | 2.51 (1.96-3.21) |  |  |
|  | Asian | 1.32 (0.37-4.64) |  |  | 2.43 (1.59-3.73) |  |  | 2.58 (1.87-3.55) |  |  | 2.61 (2.03-3.34) |  |  |
|  | Mixed/ Other | 1.05 (0.34-3.25) |  |  | 2.88 (1.77-4.68) |  |  | 2.61 (1.82-3.76) |  |  | 2.49 (1.88-3.28) |  |  |
|  | Unknown | 1.76 (1.21-2.55) |  |  | 3.55 (2.02-6.24) |  |  | 6.89 (3.56-13.33) |  |  | 2.26 (1.72-2.96) |  |  |
| **IMD (patient level)** | |  |  |  |  |  |  |  |  |  |  |  |  |
| Autonomic symptoms | 1-2 | 1.32 (1.16-1.50) | 0.6435 | ~1 | 2.25 (2.05-2.46) | 0.6091 | ~1 | 3.22 (2.92-3.54) | 0.5956 | ~1 | 2.26 (2.14-2.39) | 0.5133 | ~1 |
|  | 3 | 1.47 (1.20-1.79) |  |  | 2.39 (2.09-2.74) |  |  | 3.40 (2.95-3.91) |  |  | 2.40 (2.20-2.61) |  |  |
|  | 4-5 | 1.40 (1.20-1.63) |  |  | 2.19 (1.97-2.44) |  |  | 3.46 (3.11-3.84) |  |  | 2.32 (2.18-2.48) |  |  |
| Cognitive symptoms | 1-2 | 2.89 (0.99-8.43) | 0.5712 | ~1 | 1.46 (0.77-2.79) | 0.3890 | ~1 | 3.60 (2.16-6.00) | 0.5086 | ~1 | 2.69 (1.87-3.87) | 0.7799 | ~1 |
|  | 3 | 6.10 (2.07-17.94) |  |  | 2.45 (1.09-5.53) |  |  | 2.15 (1.05-4.40) |  |  | 2.94 (1.82-4.73) |  |  |
|  | 4-5 | 5.47 (1.87-15.99) |  |  | 2.59 (1.44-4.66) |  |  | 2.96 (1.90-4.61) |  |  | 3.21 (2.30-4.47) |  |  |
| Neurological symptoms | 1-2 | 7.89 ( 6.86-9.06) | 0.3966 | ~1 | 9.27 ( 8.39-10.24) | 0.3913 | ~1 | 12.30 (11.02-13.72) | 0.7765 | ~1 | 9.44 ( 8.86-10.06) | 0.7402 | ~1 |
|  | 3 | 8.44 ( 6.77-10.51) |  |  | 9.57 ( 8.25-11.11) |  |  | 13.17 (11.23-15.44) |  |  | 9.86 ( 8.98-10.84) |  |  |
|  | 4-5 | 7.06 ( 5.98-8.33) |  |  | 10.30 ( 9.18-11.55) |  |  | 12.46 (11.09-14.00) |  |  | 9.64 ( 8.98-10.35) |  |  |
| Pain symptoms | 1-2 | 1.69 (1.50-1.92) | 0.6916 | ~1 | 2.51 (2.30-2.74) | 0.2248 | ~1 | 3.83 (3.47-4.22) | 0.1094 | ~1 | 2.63 (2.49-2.78) | 0.1584 | ~1 |
|  | 3 | 1.77 (1.47-2.13) |  |  | 2.89 (2.53-3.30) |  |  | 4.29 (3.71-4.96) |  |  | 2.88 (2.65-3.13) |  |  |
|  | 4-5 | 1.60 (1.37-1.86) |  |  | 2.61 (2.36-2.90) |  |  | 4.44 (3.99-4.95) |  |  | 2.77 (2.60-2.94) |  |  |
| Psychiatric symptoms | 1-2 | 1.53 (1.34-1.74) | 0.3301 | ~1 | 2.14 (1.95-2.35) | 0.5008 | ~1 | 2.42 (2.19-2.66) | 0.0216* | ~1 | 2.09 (1.98-2.22) | 0.0808 | ~1 |
|  | 3 | 1.70 (1.41-2.06) |  |  | 2.37 (2.06-2.73) |  |  | 2.89 (2.51-3.33) |  |  | 2.36 (2.16-2.57) |  |  |
|  | 4-5 | 1.41 (1.21-1.65) |  |  | 2.21 (1.99-2.45) |  |  | 2.90 (2.61-3.22) |  |  | 2.21 (2.07-2.36) |  |  |
| **Location** |  |  |  |  |  |  |  |  |  |  |  |  |  |
| Autonomic symptoms | Rural | 1.34 (1.10-1.63) | 0.7775 | ~1 | 1.97 (1.70-2.27) | 0.0430* | ~1 | 2.66 (2.28-3.10) | 0.0018* | 0.1060 | 2.02 (1.84-2.20) | 0.0014* | 0.0273* |
|  | Urban | 1.38 (1.25-1.53) |  |  | 2.32 (2.16-2.48) |  |  | 3.48 (3.24-3.73) |  |  | 2.37 (2.27-2.47) |  |  |
| Cognitive symptoms | Rural | 0.00 (0.00-Inf) | 0.0198* | ~1 | 1.79 (0.64-5.03) | 0.7842 | ~1 | 2.13 (0.95-4.82) | 0.4011 | ~1 | 2.03 (1.09-3.76) | 0.1997 | ~1 |
|  | Urban | 5.58 (2.96-10.51) |  |  | 2.09 (1.38-3.15) |  |  | 3.10 (2.24-4.29) |  |  | 3.10 (2.45-3.91) |  |  |
| Neurological symptoms | Rural | 7.30 ( 5.88-9.05) | 0.5907 | ~1 | 7.59 (6.47-8.90) | 0.0010* | 0.0595 | 10.84 ( 9.12-12.87) | 0.0831 | ~1 | 8.23 ( 7.45-9.09) | 0.0010* | 0.0197* |
|  | Urban | 7.79 ( 7.00-8.68) |  |  | 10.19 (9.46-10.98) |  |  | 12.81 (11.84-13.86) |  |  | 9.90 ( 9.45-10.37) |  |  |
| Pain symptoms | Rural | 1.51 (1.23-1.84) | 0.2268 | ~1 | 2.27 (1.96-2.62) | 0.0324* | ~1 | 3.24 (2.78-3.79) | 0.0011* | 0.0665 | 2.35 (2.15-2.57) | 0.0003* | 0.0067* |
|  | Urban | 1.73 (1.57-1.90) |  |  | 2.69 (2.52-2.88) |  |  | 4.31 (4.02-4.63) |  |  | 2.80 (2.69-2.92) |  |  |
| Psychiatric symptoms | Rural | 1.33 (1.09-1.62) | 0.1382 | ~1 | 1.92 (1.65-2.23) | 0.0598 | ~1 | 2.29 (1.96-2.67) | 0.0489* | ~1 | 1.92 (1.75-2.10) | 0.0041* | 0.0830 |
|  | Urban | 1.57 (1.42-1.73) |  |  | 2.25 (2.10-2.41) |  |  | 2.72 (2.53-2.91) |  |  | 2.22 (2.13-2.32) |  |  |

**Supplementary table 7:** Prodromal symptoms according to codes used to define MS

| Table X: Comparison by MS diagnosis type | | | | | | | | |
| --- | --- | --- | --- | --- | --- | --- | --- | --- |
|  |  |  | MS Diagnosis only | | | MS diagnosis and Neurology referral | | |
| Grouped symptoms | Years prior MS diagnosis | Ethnicity | adj_or_ci | P-int | Bonferroni corrected P-int | adj_or_ci | P-int | Bonferroni corrected P-int |
| Autonomic symptoms | All pre-diagnostic years | White | 1.97 (1.89-2.04) | <0.0001* | <0.0001* | 1.81 (1.74-1.89) | <0.0001* | 0.0002* |
|  |  | Black | 2.85 (2.27-3.58) |  |  | 2.57 (2.03-3.25) |  |  |
|  |  | Asian | 2.50 (2.01-3.10) |  |  | 2.24 (1.78-2.82) |  |  |
|  |  | Mixed/ Other | 2.97 (2.31-3.83) |  |  | 2.94 (2.26-3.83) |  |  |
|  |  | Unknown | 2.87 (2.24-3.66) |  |  | 2.60 (2.01-3.37) |  |  |
|  | 0-2 yrs | White | 2.37 (2.25-2.50) | <0.0001* | <0.0001* | 2.18 (2.06-2.30) | <0.0001* | <0.0001* |
|  |  | Black | 2.92 (2.19-3.89) |  |  | 2.54 (1.87-3.45) |  |  |
|  |  | Asian | 3.05 (2.32-4.01) |  |  | 3.13 (2.33-4.20) |  |  |
|  |  | Mixed/ Other | 3.77 (2.64-5.38) |  |  | 3.16 (2.16-4.64) |  |  |
|  |  | Unknown | 8.60 (6.06-12.19) |  |  | 7.69 (5.28-11.19) |  |  |
|  | 2-5 yrs | White | 1.89 (1.80-1.99) | 0.0002* | 0.0035* | 1.70 (1.61-1.80) | 0.0005* | 0.0098* |
|  |  | Black | 2.39 (1.82-3.14) |  |  | 2.26 (1.68-3.03) |  |  |
|  |  | Asian | 2.13 (1.62-2.79) |  |  | 1.64 (1.21-2.24) |  |  |
|  |  | Mixed/ Other | 3.03 (2.18-4.21) |  |  | 2.55 (1.79-3.62) |  |  |
|  |  | Unknown | 4.14 (2.80-6.13) |  |  | 3.83 (2.55-5.75) |  |  |
|  | 5-10 yrs | White | 1.91 (1.80-2.03) | 0.0289* | 0.5788 | 1.74 (1.63-1.85) | 0.0097* | 0.1939 |
|  |  | Black | 2.67 (1.92-3.71) |  |  | 2.51 (1.74-3.61) |  |  |
|  |  | Asian | 1.90 (1.37-2.63) |  |  | 1.31 (0.89-1.94) |  |  |
|  |  | Mixed/ Other | 3.26 (2.19-4.86) |  |  | 3.07 (1.99-4.74) |  |  |
|  |  | Unknown | 2.47 (1.47-4.14) |  |  | 2.44 (1.45-4.09) |  |  |
| Cognitive symptoms | All pre-diagnostic years | White | 3.18 (2.60-3.89) | 0.0870 | ~1 | 2.45 (1.93-3.1) | 0.1807 | ~1 |
|  |  | Black | 8.74 (3.77-20.28) |  |  | 5.89 (2.03-17.07) |  |  |
|  |  | Asian | 2.67 (1.04-6.86) |  |  | 1.95 (0.64-5.96) |  |  |
|  |  | Mixed/ Other | 2.53 (0.70-9.18) |  |  | 1.79 (0.38-8.37) |  |  |
|  |  | Unknown | 10.32 (2.95-36.1) |  |  | 9.97 (2.85-34.86) |  |  |
|  | 0-2 yrs | White | 5.34 (3.88-7.36) | 0.1267 | ~1 | 4.40 (3.05-6.33) | 0.1117 | ~1 |
|  |  | Black | 13.20 (3.90-44.66) |  |  | 8.07 (1.77-36.8) |  |  |
|  |  | Asian | 7.67 (1.05-55.88) |  |  | 12.57 (2.03-77.82) |  |  |
|  |  | Mixed/ Other | 8.77 (1.71-44.85) |  |  | 3.45 (0.62-19.11) |  |  |
|  |  | Unknown | 38.42 (8.31-177.65) |  |  | 37.45 (8.10-173.15) |  |  |
|  | 2-5 yrs | White | 2.68 (1.83-3.94) | 0.5178 | ~1 | 1.90 (1.17-3.07) | 0.5152 | ~1 |
|  |  | Black | 4.98 (1.10-22.57) |  |  | 2.68 (0.24-29.64) |  |  |
|  |  | Asian | - |  |  | - |  |  |
|  |  | Mixed/ Other | - |  |  | - |  |  |
|  |  | Unknown | - |  |  | - |  |  |
|  | 5-10 yrs | White | 3.14 (1.84-5.36) | 0.6257 | ~1 | 2.07 (1.11-3.87) | 0.5670 | ~1 |
|  |  | Black | 8.10 (0.73-89.83) |  |  | 4.00 (0.25-64.18) |  |  |
|  |  | Asian | - |  |  | - |  |  |
|  |  | Mixed/ Other | - |  |  | - |  |  |
|  |  | Unknown | - |  |  | - |  |  |
| Neurological symptoms | All pre-diagnostic years | White | 8.47 ( 8.11-8.84) | <0.0001* | <0.0001* | 7.58 ( 7.24-7.93) | <0.0001* | <0.0001* |
|  |  | Black | 11.56 ( 9.01-14.84) |  |  | 10.49 ( 8.07-13.63) |  |  |
|  |  | Asian | 10.18 ( 8.08-12.84) |  |  | 8.56 ( 6.70-10.93) |  |  |
|  |  | Mixed/ Other | 16.01 (12.12-21.15) |  |  | 13.98 (10.46-18.70) |  |  |
|  |  | Unknown | 15.38 (12.04-19.65) |  |  | 14.82 (11.50-19.11) |  |  |
|  | 0-2 yrs | White | 18.30 (17.20-19.48) | <0.0001* | <0.0001* | 17.31 (16.21-18.49) | <0.0001* | <0.0001* |
|  |  | Black | 20.10 (14.48-27.91) |  |  | 20.70 (14.57-29.39) |  |  |
|  |  | Asian | 17.60 (13.32-23.25) |  |  | 15.23 (11.33-20.46) |  |  |
|  |  | Mixed/ Other | 34.72 (24.04-50.14) |  |  | 37.30 (24.68-56.37) |  |  |
|  |  | Unknown | 51.18 (36.39-71.99) |  |  | 51.44 (36.33-72.85) |  |  |
|  | 2-5 yrs | White | 5.07 ( 4.74-5.41) | 0.0007* | 0.0140* | 3.88 ( 3.61-4.18) | 0.0099* | 0.1988 |
|  |  | Black | 6.88 ( 4.88-9.70) |  |  | 5.28 ( 3.57-7.81) |  |  |
|  |  | Asian | 4.64 ( 3.44-6.26) |  |  | 3.37 ( 2.38-4.78) |  |  |
|  |  | Mixed/ Other | 6.07 ( 4.17-8.82) |  |  | 4.92 ( 3.23-7.50) |  |  |
|  |  | Unknown | 14.27 ( 8.93-22.79) |  |  | 10.11 ( 5.82-17.57) |  |  |
|  | 5-10 yrs | White | 4.22 ( 3.89-4.57) | 0.1740 | ~1 | 3.42 ( 3.13-3.75) | 0.1334 | ~1 |
|  |  | Black | 5.05 ( 3.23-7.91) |  |  | 3.94 ( 2.28-6.81) |  |  |
|  |  | Asian | 3.57 ( 2.39-5.34) |  |  | 2.16 ( 1.25-3.73) |  |  |
|  |  | Mixed/ Other | 5.41 ( 3.31-8.85) |  |  | 3.97 ( 2.29-6.88) |  |  |
|  |  | Unknown | 8.31 ( 4.56-15.16) |  |  | 6.74 ( 3.54-12.86) |  |  |
| Pain symptoms | All pre-diagnostic years | White | 2.30 (2.21-2.39) | <0.0001* | <0.0001* | 2.14 (2.06-2.23) | <0.0001* | 0.0006* |
|  |  | Black | 3.35 (2.66-4.21) |  |  | 2.97 (2.35-3.76) |  |  |
|  |  | Asian | 2.96 (2.38-3.68) |  |  | 2.71 (2.16-3.40) |  |  |
|  |  | Mixed/ Other | 3.98 (3.09-5.13) |  |  | 3.48 (2.68-4.52) |  |  |
|  |  | Unknown | 2.87 (2.25-3.64) |  |  | 2.76 (2.15-3.53) |  |  |
|  | 0-2 yrs | White | 3.30 (3.13-3.47) | <0.0001* | <0.0001* | 3.10 (2.94-3.27) | <0.0001* | <0.0001* |
|  |  | Black | 5.45 (4.16-7.14) |  |  | 4.45 (3.32-5.95) |  |  |
|  |  | Asian | 3.44 (2.68-4.42) |  |  | 3.38 (2.59-4.42) |  |  |
|  |  | Mixed/ Other | 4.43 (3.17-6.18) |  |  | 4.45 (3.12-6.34) |  |  |
|  |  | Unknown | 9.88 (6.81-14.35) |  |  | 9.66 (6.68-13.97) |  |  |
|  | 2-5 yrs | White | 2.36 (2.25-2.48) | <0.0001* | <0.0001* | 2.10 (1.99-2.21) | <0.0001* | 0.0011* |
|  |  | Black | 3.12 (2.39-4.06) |  |  | 2.48 (1.86-3.30) |  |  |
|  |  | Asian | 2.94 (2.31-3.74) |  |  | 2.59 (2.00-3.36) |  |  |
|  |  | Mixed/ Other | 3.50 (2.58-4.75) |  |  | 2.73 (1.94-3.85) |  |  |
|  |  | Unknown | 6.14 (4.32-8.74) |  |  | 5.49 (3.77-7.99) |  |  |
|  | 5-10 yrs | White | 2.21 (2.08-2.33) | 0.0121* | 0.2410 | 1.94 (1.82-2.06) | 0.0189* | 0.3783 |
|  |  | Black | 2.52 (1.81-3.49) |  |  | 2.21 (1.52-3.23) |  |  |
|  |  | Asian | 2.34 (1.73-3.15) |  |  | 1.96 (1.40-2.73) |  |  |
|  |  | Mixed/ Other | 3.66 (2.54-5.28) |  |  | 3.37 (2.27-5.01) |  |  |
|  |  | Unknown | 3.91 (2.51-6.07) |  |  | 3.36 (2.10-5.37) |  |  |
| Psychiatric symptoms | All pre-diagnostic years | White | 1.84 (1.76-1.91) | <0.0001* | <0.0001* | 1.70 (1.63-1.77) | <0.0001* | 0.0001* |
|  |  | Black | 2.91 (2.30-3.68) |  |  | 2.52 (1.96-3.23) |  |  |
|  |  | Asian | 2.89 (2.30-3.64) |  |  | 2.61 (2.04-3.34) |  |  |
|  |  | Mixed/ Other | 2.82 (2.17-3.67) |  |  | 2.49 (1.89-3.29) |  |  |
|  |  | Unknown | 2.39 (1.84-3.10) |  |  | 2.27 (1.73-2.97) |  |  |
|  | 0-2 yrs | White | 2.79 (2.64-2.96) | <0.0001* | <0.0001* | 2.49 (2.34-2.64) | <0.0001* | <0.0001* |
|  |  | Black | 5.64 (4.01-7.94) |  |  | 4.30 (2.97-6.23) |  |  |
|  |  | Asian | 4.65 (3.30-6.56) |  |  | 4.14 (2.85-6.00) |  |  |
|  |  | Mixed/ Other | 6.00 (4.08-8.81) |  |  | 5.23 (3.48-7.85) |  |  |
|  |  | Unknown | 8.79 (5.84-13.23) |  |  | 8.33 (5.46-12.73) |  |  |
|  | 2-5 yrs | White | 2.13 (2.02-2.25) | <0.0001* | <0.0001* | 1.99 (1.88-2.11) | <0.0001* | <0.0001* |
|  |  | Black | 3.60 (2.63-4.93) |  |  | 3.52 (2.53-4.90) |  |  |
|  |  | Asian | 3.22 (2.34-4.44) |  |  | 3.18 (2.27-4.46) |  |  |
|  |  | Mixed/ Other | 3.87 (2.70-5.54) |  |  | 3.28 (2.23-4.82) |  |  |
|  |  | Unknown | 4.56 (3.03-6.86) |  |  | 4.05 (2.65-6.18) |  |  |
|  | 5-10 yrs | White | 1.96 (1.84-2.09) | 0.0028* | 0.0570 | 1.75 (1.63-1.87) | 0.0101* | 0.2026 |
|  |  | Black | 2.58 (1.76-3.79) |  |  | 2.58 (1.69-3.93) |  |  |
|  |  | Asian | 2.78 (1.91-4.04) |  |  | 2.36 (1.52-3.68) |  |  |
|  |  | Mixed/ Other | 4.18 (2.73-6.41) |  |  | 3.57 (2.24-5.69) |  |  |
|  |  | Unknown | 2.46 (1.39-4.36) |  |  | 2.32 (1.28-4.20) |  |  |
